# Identification of novel conserved functional motifs across most Influenza A viral strains

**DOI:** 10.1186/1743-422X-8-44

**Published:** 2011-01-27

**Authors:** Mahmoud ElHefnawi, Osama AlAidi, Nafisa Mohamed, Mona Kamar, Iman El-Azab, Suher Zada, Rania Siam

**Affiliations:** 1 Informatics and Systems Department and Biomedical Informatics and chemo informatics group, Division of Engineering Research and Centre of Excellence for Advanced Sciences, National Research Centre, Tahrir Street, 12311 Cairo, Egypt; 2 Yousef Jameel Science and technology Research Center, The American University in Cairo, New Cairo, Egypt; 3 Department of Biochemistry, University of Saskatchewan, Canada; 4 Faculty of Computers & Information, Cairo University, Ahmed Zowail Street, Cairo, Egypt; 5 Biology Department, American University in Cairo, Cairo, Egypt

## Abstract

**Background:**

Influenza A virus poses a continuous threat to global public health. Design of novel universal drugs and vaccine requires a careful analysis of different strains of Influenza A viral genome from diverse hosts and subtypes. We performed a systematic *in silico *analysis of Influenza A viral segments of all available Influenza A viral strains and subtypes and grouped them based on host, subtype, and years isolated, and through multiple sequence alignments we extrapolated conserved regions, motifs, and accessible regions for functional mapping and annotation.

**Results:**

Across all species and strains 87 highly conserved regions 	(conservation percentage > = 90%) and 19 functional motifs (conservation percentage = 100%) were found in PB2, PB1, PA, NP, M, and NS segments. The conservation percentage of these segments ranged between 94 - 98% in human strains (the most conserved), 85 - 93% in swine strains (the most variable), and 91 - 94% in avian strains. The most conserved segment was different in each host (PB1 for human strains, NS for avian strains, and M for swine strains). Target accessibility prediction yielded 324 accessible regions, with a single stranded probability > 0.5, of which 78 coincided with conserved regions. Some of the interesting annotations in these regions included sites for protein-protein interactions, the RNA binding groove, and the proton ion channel.

**Conclusions:**

The influenza virus has evolved to adapt to its host through variations in the GC content and conservation percentage of the conserved regions. Nineteen universal conserved functional motifs were discovered, of which some were accessible regions with interesting biological functions. These regions will serve as a foundation for universal drug targets as well as universal vaccine design.

## Background

The influenza A virus is a major threat to world health and economy. The polymerase of this RNA virus lacks proof reading activity [1], which gives rise to considerable viral variability culminating in the 3 different types A, B and C, in addition to many subtypes based on variations in the hemagglutinin (HA) and the neuraminidase (NA) surface proteins [2]. The influenza genome consists of 8 RNA segments and encodes 10 proteins including the internal structural proteins, nucleocapsid protein (NP), and the two matrix proteins (M1 & M2) [3,4].

The surface proteins neuraminidase (NA) and hemagglutinin (HA) have been studied extensively and the antigenic variations in the these surface glycoproteins are used to subtype influenza A. Additionally, three of the influenza polypeptides are associated with RNA polymerase activity (PA, PB1, PB2). The RNA binding non-structural protein (NS) contributes to viral pathogenicity and plays a central role in the prevention of interferon mediated antiviral response [3,4].

Genetic reassortment of the Influenza A virus within different hosts (including avian and swine), and antigenic shifts and drifts in the HA and NA proteins, are the cause of widespread pandemics in immunologically unfamiliar populations. These have resulted in serious outbreaks and pandemics, such as those of 1918, 1957, 1968, and 2009 [5]. This change in genetic and antigenic composition, presents an ever-present challenge for the development of influenza vaccines and antiviral medications.

Bioinformatics has played a major role in several aspect of virology research; these include predicting viral RNA structure [6], the structural and functional analysis of viral proteins [7], and immunoinformatics to predict epitopes and reverse vaccinology [8]. Such studies have assisted the development of biomarkers for the diagnosis, staging, and prognosis [9] of viruses (for a review see [10]). Additionally, computer-aided drug designs have led to the identification and validation of drugs [11] for many major viruses, such as HIV, influenza and HCV [12], helping the world face the challenges of such major viral diseases with a huge medical care burden [13,14]. Molecular modelling studies have in addition provided mechanistic explanations for such questions like drug modes of action, virus-receptor interaction, and virus-host interactions. In these lines of research, conserved regions found in viruses, extrapolated from multiple sequence alignments of different strains, were essential in functional prediction through the identification of epitopes and motifs [15-17].

Several studies have addressed different aspects of the influenza virus, its evolution, structure, and function analysis, to delineate the molecular mechanisms of pathogenicity and continuous resistance to immune response. Several previous studies performed phylogenic analysis and addressed the evolution of one or more Influenza A viral segments [18]. Additionally, methodical analysis of the whole genome has identified co-occurrence of mutation networks and other properties, such as relative codon usage (rscu) and codon usage patterns (cup), as features of Influenza evolution [19]. Motif prediction in the HA influenza genes and proteins has been previously conducted [17].

Our study is a comprehensive systematic comparative nucleotide genomic analysis that complements prior analyses and utilizes complete influenza viral segments isolated from different hosts such as humans, avians, swine, and a fourth group for all other hosts, that belong to different HA and NA subtypes, and from different geographic regions and years. The main theme of the current study is genome conservation among different strains. This is achieved by the utilization of all available complete segment sequences from the NCBI's Influenza Virus Resource database in order to achieve a reasonable comparative analysis between the main three hosts: human, swine, and avian, to highlight regions that could serve as targets for universal drug and vaccine design. The need for high sequence conservation as a prerequisite of efficient siRNA design for the Influenza A virus has been highlighted previously [20]. The identification of conserved regions in the influenza M gene has been previously reported[21].

In the current study, meta-analysis of the Influenza A viral genome segments from different hosts, different subtypes, and different geographic regions is performed. Genomic conserved regions across all diverse strains and hosts are extracted by multiple sequence alignments and the conservation percentage is calculated. An analysis of inter- and intra- host strains segmental genomic variability of Influenza A viral segments for human, avian, and swine hosts, and the GC percentage of the segments in the different hosts, is also conducted. Completely conserved genomic functional motifs are identified and analysed through functional annotation. This work will not only provide understanding of the natural selection of the Influenza A virus, but will serve as a foundation for gene therapy, and novel Influenza A universal drug and vaccine design to target highly conserved regions with crucial functions. Moreover, the bioinformatics sequence analysis workflow that is presented and applied could be used for research into the understanding of the evolution of viruses and the design of universal drug targets.

## Results & Discussion

Preprocessing and alignment of Influenza sequences.

More than twenty-two thousand complete segment sequences of the Influenza A virus were downloaded from the NCBI's Influenza Virus Resource [22]. The sequences downloaded for each segment from the NCBI website for six out of the eight positive-sense RNA influenza segments are illustrated in Table 1. Sequences of swine, avian, and human strains were grouped based on the host; and avian and human strains were sub-classified based on the variations in hemagglutinin proteins (illustrated in the sequence grouping in the material section). The entire nucleotide sequences, in addition to each core coding sequence for each segment (PB2, PB1, PA, NP, M, NS, HA, and NA), were compared. The MUSCLE 3.6 program [23] was used for sequential alignment of human and swine strains, and this was followed by avian strains sequence alignment. (The multiple sequence alignment files of all hosts for each of the studied segments are available in additional files 1, 2, 3, 4, 5, and 6.)

**Table 1:** Number of sequences downloaded and utilized in this study for each of the influenza viral segments and their conserved regions, the longest conserved region of each segment and the conserved regions with highest conservation percentage are recorded

Segment	Number of sequences	Number of conserved regions	Longest conserved region in each segment	Conserved regions with highest conservation percentage	
				
				Conserved region	Percentage
**1-PB2**	3538	13	Region 12 (from nt 2165 to 2317)	PB2 - 12: Position 2165 to 2317	96.73%

**2-PB1**	3319	25	Region 2 (from nt 230 to 493)	PB1 - 22: Position 2012 to 2064	98.06%

**3-PA**	3720	18	Region 7 (from nt 690 to 677)	PA-6: Position 621 to 677	98.16%

**5-NP**	3776	14	Region 1 (from nt 62 to 161)	NP-13: Position 1447 to 1486	97.08%

**7-M**	3605	8	Region 8 (from nt 733 to 1000)	M-6: Position 599 to 663	97.22%

**8-NS**	4125	9	Region 6 (from nt 492 to 700)	NS-1: Position 59 to 137	98.96%

***TOTAL***	22083	87			

**Figure 1:**
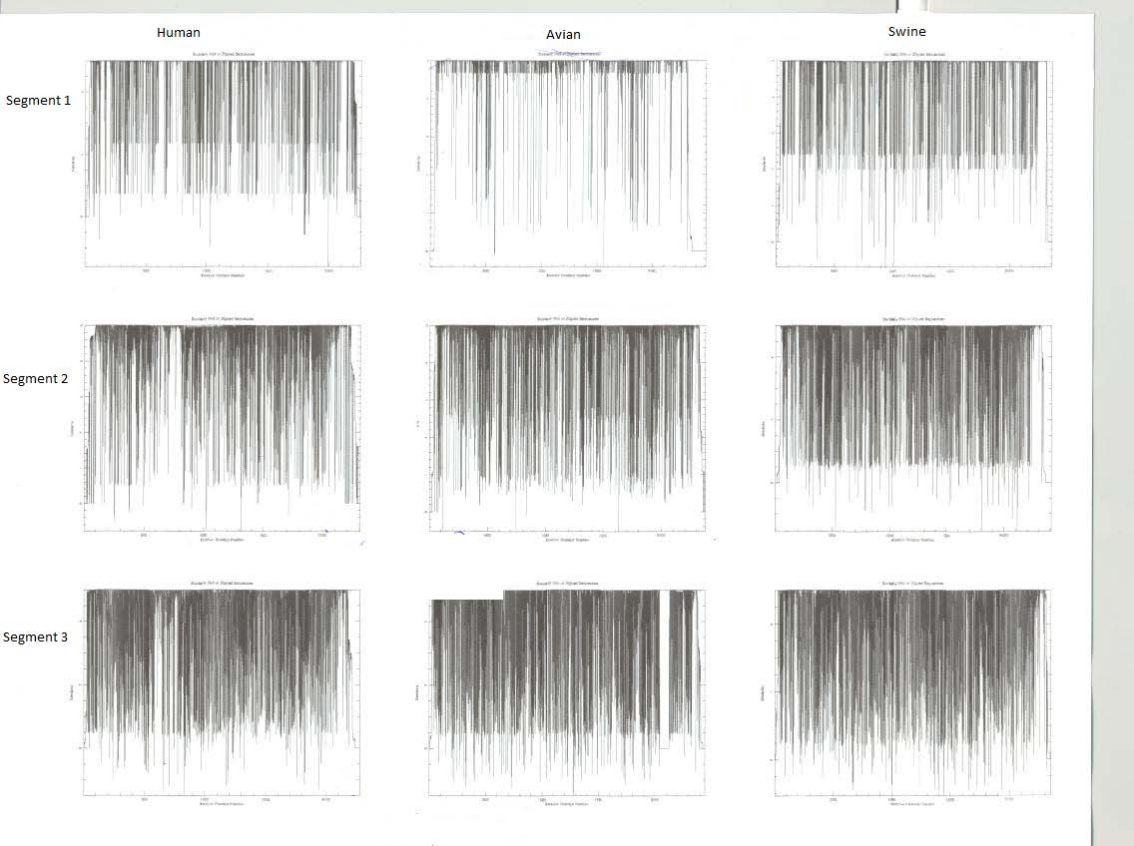
**Influenza A viral PB2, PB1, and PA segment conservation plots in human, avian, and swine strains**. The name of each segment is shown horizontally and name of host shown vertically. The figures were generated with the PLOTCONS tool from the EMBOSS package. Insights into different host evolution and conservation in the different segments can be inferred from the figure. The plots show that swine strains are the most variable.

**Figure 2:**
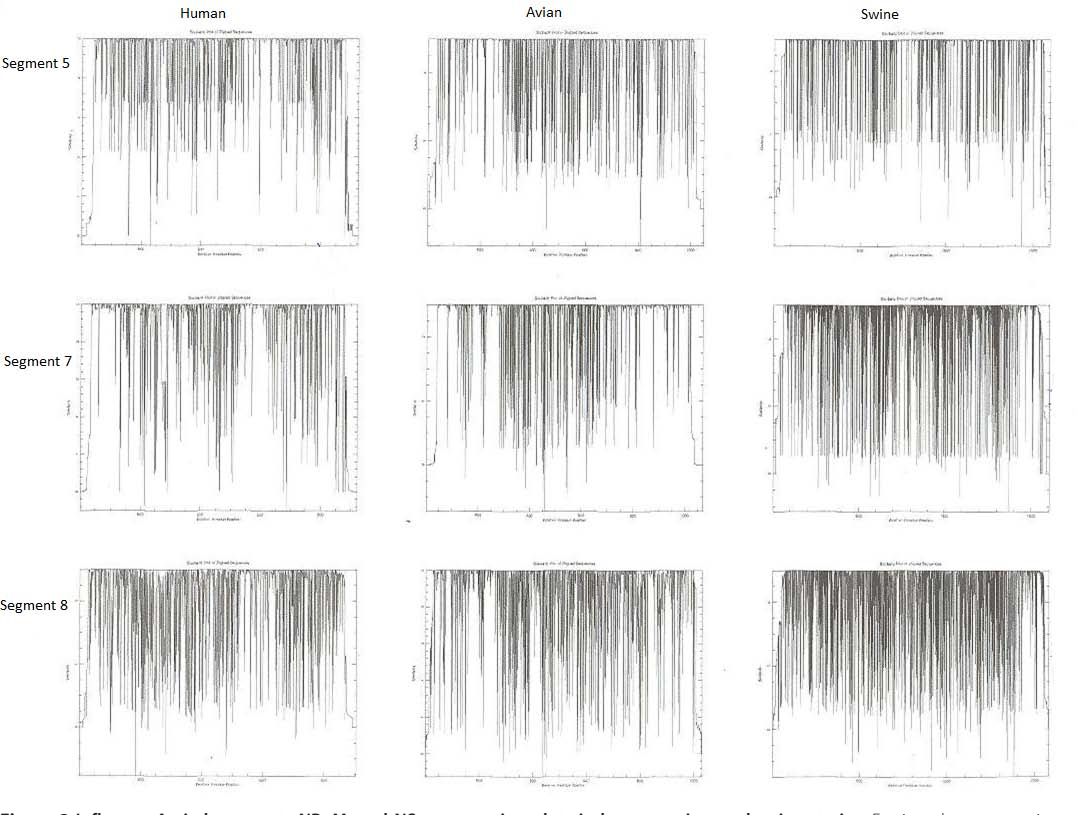
**Influenza A viral segments NP, M, and NS conservation plots in human, avian, and swine strains**. For intra-host comparisons, the PB1 segment is the most conserved in human strains. This is followed by PB2, NP, PA, NS, and finally M. In avian strains, NS is most conserved, followed by M, PB1, NP, PA, and finally PB2. In swine strains, M is most conserved followed by NP, PB2, PA, and finally PB1.

This enabled a comparison between human, swine, and avian strains and a conservation profile of these studied segments is illustrated in figures 1 and 2. Conserved nucleotide regions in each of the studied segments were extracted using the Bioedit program; and the consensus sequences derived from the alignments of Influenza A viral segments were calculated.

### Inter- and Intra- host strains conservation and variability analysis of the influenza segments

Our approach in grouping the sequences according to species infected/ host isolated from enabled an analysis of inter- and intra- species conservation and variability. A comparison of the inter- and intra- host alignments of the influenza segments using the Plotcon and Infoalign tools results shows that swine strains are the most variable (similarity plots illustrated in figures 1 and 2). This result was expected, since the swine strains can mix with both avian and human influenza strains. The human strains are the most conserved except in segment PB2, where the avian is more conserved. The conservation percentage of the segments ranged between 94 - 98% in human strains (the most conserved), 91 - 94% in avian strains and 85 - 93% in swine strains (the most variable). On the other hand, intra-segmental comparisons reveal that the PB1 segment is the most conserved in human strains (98.1%), followed by PB2, NP, PA, NS, and finally the M segment. For the avian host strains, the NS and M segments show the most conservation (94.5%), followed by the PB1, NP, PA, and finally the PB2 segment. In the swine strains the M segment shows the most conservation followed by the segments NP, PB2, PA, and finally PB1.

### Identification of 87 conserved regions in the influenza genome

**Figure 3:**
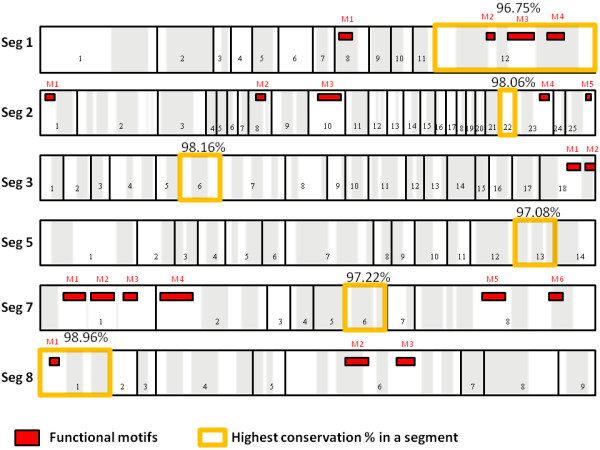
**Schematic representation of the mapping of conserved regions, functional motifs, and accessible regions to conserved regions on the six studied Influenza A viruses**. The functional motifs, referred to as(M), are highlighted in red and numbered in each segment (m1, m2, etc), the accessible regions are highlighted in grey, and the highly conserved regions are framed in orange. The location, length and conservation percentage of each segment are shown in additional file 7. The location length and sequence of each functional motif is shown in table 3. The location and length of each accessible region is shown in additional file 9.

Conserved regions were extracted by entropy calculation; these were regions with at least 21 nucleotides in length and a maximum of 2 mismatches. We identified several conserved regions in the PB1, PB2, PA, NP, M, and NS segments. This approach has not identified any conserved regions present in HA and NA segments. Additional file 7 represents each segment and the position and size (length) of the conserved region. The mapping and position of each conserved region to each influenza genome segment is illustrated in figure 3. We found 13 conserved regions in segment 1, 25 conserved regions in segment 2, 18 conserved regions in segment 3, 14 conserved regions in segment 5, 8 conserved regions in segment 7, and 9 conserved regions in segment 8. We have correlated the biological functions of these conserved regions in the Influenza A virus life cycle through the annotation of conserved regions and motifs for functional motif identification (discussed below). Logo bars of the conserved regions are shown in additional file 8. These logo bars are useful visualization tools that signify conservation of each position in the conserved regions. The logo bars are ordered sequentially by segment and conserved region.

### GC Percentage

The GC percent in the different Influenza A virus segments and in the different hosts are illustrated in Table 2. The average GC percent in PB2 was 44%, 42% in PB1, 44% in PA, 47% in NP, 48% in M, and 43% in NS. The GC percent was highest in avian strains, followed by human strains, which may reflect the adaptation of influenza virus to the warmer host temperature of avians as compared to swine and humans.

**Table 2:** The influenza genome segments conservation and GC percentage in the different hosts

	Conservation % & GC% in Segments					
**Species**	**PB2**	**PB1**	**PA**	**NP**	**M**	**NS**

**Human**	96.47 0.42	98.16 0.42	95.09 0.42	95.92 0.46	94.34 0.47	95.01 0.43

**Swine**	90.44 0.43	85.36 0.41	89.57 0.42	91.27 0.45	93.42 0.46	90.68 0.44

**Avian**	91.06 0.45	93.93 0.42	93.34 0.44	93.35 0.47	94.51 0.48	94.67 0.44

**HSAO**	89.32 0.44	89.32 0.42	89.56 0.44	90.48 0.47	93.47 0.48	90.49 0.43

**Others**	92.23 0.44	91.87 0.43	90.79 0.43	90.05 0.46	94.07 0.48	85.63 0.43

* The second number is the GC %.

The conservation percentage for each species is shown and was calculated using Infoalign from EMBOSS

### Identification of functional motifs and annotation of conserved regions and motifs

In our analysis, sixor more nucleotide blocks that are conserved completely (100%) in different Influenza a viral strains, and across multiple hosts, were identified as motifs. Our identified motifs are tabulated in Table 3. The perfect conservation of these motifs suggests biological significance and a potential critical role in the influenza viral life cycle.

**Table 3:** Evolutionary highly conserved motifs in Influenza A virus

Segment	Motif	Length	start position (consensus sequence)	End Position (Consensus Sequence)	H5N1 Start Position	H5N1 End Position	Sequence	Repeated Positions On H5N1	Mapping on Conserved Regions
**1 (PB2)**	1	9	1652	1660	1631	1639	TGATGTGGG		R8

**1 (PB2)**	2	6	2254	2259	2232	2237	GAAACG	(2238 - 2243)	R12

**1 (PB2)**	3	11	2273	2283	2251	2261	AGCATACTTAC		R12

**1 (PB2)**	4	9	2291	2299	2269	2277	CAGACAGCG		R12

**1 (PB2)**	1	6	914	919	889	894	ATGATG	(1243 - 1248) (1246 - 1251) (1624 - 1629) (1994 - 1999)	R8

**2 (PB1)**	2	11	1268	1278	1243	1253	ATGATGATGGG		R10

**2 (PB1)**	3	6	2354	2359	2248	2253	GAGATC	(101 to 106)	R23, R25

**2 (PB1)**	4	6	2391	2396	2285	2290	GACGGC		R24, R25

**3 (PA)**	1	8	2140	2147	2095	2102	GAGGAGTG		R18

**3 (PA)**	2	6	2150	2155	2105	2110	TGATTA		R18

**7 (M)**	1	9	93	101	77	85	GGCCCCCTC		R1

**7 (M)**	2	11	180	190	164	174	AAGACAAGACC		R1

**7 (M)**	3	8	196	203	180	187	TGTCACCT		R1

**7(M)**	4	17	237	253	221	237	CTCACCGTGCCCAGTGA		R2

**7 (M)**	5	7	873	879	855	861	TTCAAAT		R8

**7 (M)**	6	8	945	952	927	934	ATGAGGGA		R8

**8 (NS)**	1	7	69	75	43	49	AGGTAGA		R1

**8 (NS)**	2	10	574	583	529	538	AGGATGTCAA		R6

**8 (NS)**	3	6	616	621	571	576	AATGGA	( 368- 373) (383- 388)	R6

The evolutionary conserved motifs found in four of the eight influenza segments, whose criteria are at least 6 consecutive nucleotides that are 100% conserved in all the studied strains and the location of each motif on H5N1 Refseq. Sequences and length are shown

We found 4 motifs in the PB2 segment; motif 2 (GAAACG) is repeated twice in the H5N1 reference sequence; and motifs 2, 3, and 4 were previously identified as a conserved region involved in RNA packaging [24]. Interestingly, motif 3 also partially overlaps the nuclear localization signal (NLS). In segment PB1, four motifs were found; motif 1 (ATGATG) is repeated five times and motif 3 (GAGATC) is repeated twice on the H5N1 reference sequence. In PA, two motifs were identified that overlap with RNA packaging annotations [24]. Segment M contains six motifs; motif 4 is the longest (CTCACCGTGCCCAGTGA). In segment NS, three motifs were found; and motif 3 (AATGGA) is repeated three times on the H5N1 reference sequence.

Functional annotation of the conserved regions and motifs was also performed by mapping of the regions and motifs on the 3D structure. Structural mapping of these conserved regions on the available influenza domains from PDB revealed many interesting functions, explaining their selection for conservation.

**Figure 4:**
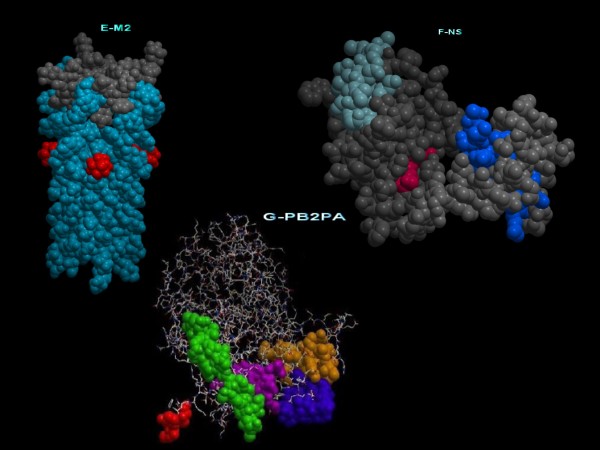
**Functional annotations deduced by mapping of some of the conserved regions on influenza viral protein 3D structures**. The conserved regions translated to amino acids and then mapped on the 3D structure of proteins are presented. Several tools and databases including Swiss Prot and PDB sum were used for annotation.

Three of the functional motifs in PB2 lie in conserved region 12 and are expressed mostly on the surface of the PB2 protein. In segment PA two functional motifs, with genomic sequences (GAGGAGUG, UGAUUA), are mapped to conserved region 18. They are mostly accessible on the surface amino acids of the domain which interact with the PB2 protein [25]. In the M2 protein, four functional motifs with genome sequences (GGCCCCCUC, AAGACAAGACC, UGUCACCU, CUCACCGUGCCCAGUGA) are mapped to conserved region 1, which encompasses the proton ion channel as illustrated in figure 4a[26].

Analysis of many conserved regions in PB2 and PA revealed that they are mostly on the surface and are involved in protein-protein interactions. The same applies to the NP protein. Interestingly, conserved regions 5, 6, and 7 together form the RNA binding groove (ElHefnawi et. al., submitted).

There are three large conserved regions (Cr2, Cr6, Cr9) found on the NS1 protein [27] (PDB id: 3F5T) that is expressed by segment 8 of the virus genome (represented in figure 4b). They lie mainly on the surface of the protein and may play an important roles in the binding of different molecules and ligands that contribute to the promiscuity of the NS1 in its immune counterattack mechanisms. Clefts found in these conserved regions could bind to different immune system components (Figure 4c). Cr6 contains four functional motifs with sequences of (AGGTAGA, AGGATGTCAA and three motifs of the sequence AATGGA). The immune system interception functions of the NS1 protein are quite similar to those of the NS5A protein of Hepatitis C virus that was shown previously to have different immune system counterattack mechanisms[28]. This is an interesting property of many viruses that deserves further analysis.

### Predictions of accessible target regions and mapping to conserved regions

We have also assessed accessible regions and mapped them to conserved regions to infer their potential use as drug targets. Understanding accessible regions is a critical factor; for example, at least half of the siRNA target region needs to be accessible preferentially in the terminal ends. Therefore, the accessibility of the segments was calculated using the SFOLD server. We located 324 regions on six segments and mapped them to conserved regions (Figure 3). In PB2, ten accessible regions mapped to conserved regions, in PB1, 24; in PA, 16; in NP, 14; in M, 7; and in NS, 7. The accessible regions, which overlap with functional motifs, are presented in figure 3 and additional file 9.

## Conclusions

This *in silico *study analyzed Influenza A virus genome segments available in the Influenza A virus resource at NCBI and grouped them according to host, strain, and year to determine conserved regions across all species studied. The higher variability in the influenza sequences isolated from swine host suggests greater hazards in future pandemics. The higher GC percentage of Influenza sequences infecting avian hosts indicates adaptation to the higher host temperature. The evolution of the influenza virus is driven by adaptation mechanisms to its host. Identification of highly-conserved functional motifs and accessible regions of all sequences was obtained. Eighty-seven conserved regions, nineteen functional motifs, and many potentially accessible regions were identified. These data on the Influenza A virus segments were utilized in the optimal design of universal therapeutic small interfering RNA molecules. The complete workflow including the siRNA design and selection figure will be presented in the next publication(ElHefnawi, submitted) and can help in other future drug and vaccine design.

## Methods

### Collection of sequences

Complete sequences for all segments of Influenza A virus were downloaded in groups using the advanced database search at the NCBI's Influenza Virus Resource [22]. We utilized both the entire nucleotide sequences, in addition to coding sequences for single segments encoding the following proteins; segments PB2, PB1, PA, NP, M, NS, HA, and NA. We utilized approximately 30,000 influenza sequences for the eight segments. The number of sequences utilized from each segment is represented in Table 1.

### Grouping of sequences

To facilitate the analysis process we divided each segment based on the infected host as follows:

(A) Swine strain sequences

(B) Avian strain sequences were sub divided into the following groups:

1) H9 and Mixed strains

2) H8, H7

3) H6

4) H4, H3

5) H1, H2

6) H10, H11, H12, H13, H14, H15, H16

(C) Human strain sequences were divided into the following groups:

1) H9, H7, H5

2) H1 strains were further subdivided, based on the year of isolation, into the following two subcategories; H1 strains isolated between 1918 and 2000, and H1 strains isolated between 2001 and 2007.

3) H2

4) H3 strains were further subdivided based on year of isolation into the following three subcategories; H3 isolated between 1968 and 1998, between 1999 and 2002, and between 2003 and 2007.

D) Miscellaneous: all other strains infecting species other than avian, human and swine.

The above categorization of the sequences facilitated the management of the data, allowed the identification of diversity in the sequences based on the host and year isolated, and helped in the determination of conservations amongst strains. This categorization allowed us to conduct comparative mutational analysis in all segments followed by the calculation of conservation percentage. Such subtype classification according to the immunological nature of strains, and identification of the similarity of structural proteins across strains, combined with sub-categorization at the nucleotide level, will facilitate drug design as siRNA data mining.

### Alignment and conservation analysis

#### Multiple Sequence Alignments of whole Influenza segments

The program MUSCLE version 3.6 [23] was used to align primary sequence groups. The resulting aligned sequences were aligned by profile-profile alignment using the same MUSCLE 3.6 program.

First the alignments were performed by aligning strains isolated from the same host, as discussed above, where avian strains were aligned separately from human and swine strains. Second, human and swine strain sequences were aligned, and the resulting file was aligned with the avian sequence file, and then all other host strains. This order was followed because human and swine strains are generally more homologous than avian strains. For similar reasons, the avian strains were added before the other host species. Based on phylogenetic distances, such an order in the alignment sequences enhances conservation finding and facilitates the management of diversity in sequences.

### Emboss Analysis

The BIOPERL[29] modules were used for automating the analysis of the alignments using different tools from EMBOSS like GeeCee [30], Logobar [31], Infoalign [30], Cons [30] and Plotcon [30]. Scripts were written for each of these tools and run under the Biolinux operating environment [32]. These scripts are available upon request. The consensus sequence for each segment was calculated using the Cons tool from EMBOSS [30] and submitted to Genbank.

### Inter- and intra- host genomic conservation analysis

Conservation and variability across the eight IAV segments in the different hosts was studied by plotting the conservation of the alignments using the Plotcon tool from EMBOSS [30]. Additionally, the Infoalign tool from EMBOSS was used to calculate the conservation percentage of the segments in the different hosts in order to study inter-species and intra-host variability [30] (Table 2). The GC % for each segment was also calculated using the GeeCee tool from EMBOSS as shown in Table 2.

### Conserved region identification

Conserved nucleotide regions were extracted using the Bioedit program [33].

#### I- Entropy calculation

Mining for conserved sequences among the aligned sequences was performed by determining the entropy of regions with at least 21 nucleotides in length with a maximum of 2 mismatches. Therefore, we defined an area as conserved if 19 identical continuous nucleotides were detected in all strains with an additional 2-nucleotide mismatch (total 21 nucleotides).

#### II-Conservation mapping

Entropy calculation was followed by checking the number of mismatches in each of our identified conserved regions. The conserved regions were mapped to the 8 segments on the influenza virus as illustrated in figure 3 and additional file 7.

Logo bars for all conserved regions were generated using the logo bar tool (additional file 8). The conservation percent of every conserved region was calculated using Infoalign from EMBOSS [30] and tabulated in additional file 7.

### Functional motifs identification and annotation of conserved regions and motifs

One-hundred-percent conserved motifs of a minimum length of 6 bp in all IAV segments were extracted using the BIOEDIT program [33]. The motifs were mapped to the H5N1 reference genome, and to the conserved regions (Table 3). Also, the H5N1 avian flu reference sequence was checked for other occurrences of these motifs. The perfect conservation of these motifs suggests biological significance and a potential role in the Influenza life cycle.

Functional annotation of the conserved regions and functional motifs was performed after mapping them on the PDB 3D protein files of their segments, and using annotations available for these proteins from the PDB SUM server [34]. After downloading the relevant structure files we highlighted the conserved regions on the structure to show their positions and configuration. Then we used the annotation knowledge gained from the PDBsum for linking the regions with their correlated functions. The annotation at the genome level was performed using Rfam in order to search for conserved regions in RNA structures with specific annotations.

### Genomic accessibility forRNAi-based therapeutic design

The SFOLD tool was used to calculate the target accessibility of the Influenza segments using the consensus sequence for each segment calculated from the multiple sequence alignment [35]. A region was considered accessible if at least the average single stranded probability using Sfold was greater than 0.5 for 9 consecutive nucleotides. The results are tabulated in additional file 9 and the regions that map to conserved regions are highlighted in figure 3.

## Competing interests

The authors declare that they have no competing interests.

## Authors' contributions

The formulation of the study was by ME and OA. ME and OA also conceived the study and its design and experiments, and helped in the performance with NM and MK. Analysis of results by NM, MK, IE, RS, and SZ. Writing by ME, MK, NM, and IE. Revision by ME, RS, IE and SZ. Scripting by OA, MK and NM. All authors read and approved the final manuscript.

## Supplementary Material

Additional file 1 **Multiple sequence alignments of the PB2 segment**. The Sequence collection, grouping, ordering, and alignment were all performed as elaborated in the methods section, and conserved regions and functional motifs of PB2 extracted from the alignment. Also, Logo bars of the conserved regions, the conservation percent of each conserved region, and average GC% were all carried out as elaborated in methods. Separation of the alignments into the four main host categories (human, swine, avian, and others) was conducted to facilitate comparative host analysis as elaborated in figures 1 and 2. The same applies to additional files 2, 3, 4, 5, and 6. Click here for file

Additional file 2 **Multiple sequence alignments of the PB1 segment**. Click here for file

Additional file 3 **Multiple sequence alignments of the PA segment**. Click here for file

Additional file 4 **Multiple sequence alignments of the NP segment**. Click here for file

Additional file 5 **Multiple sequence alignments of the M segment**. Click here for file

Additional file 6 **Multiple sequence alignments of the NS segment**. Click here for file

Additional file 7 **Conserved regions in the Influenza A viral segments**. The position, length, conservation percent of each conserved region is shown. Click here for file

Additional file 8 **Logobars of conserved regions**. Eighty-seven logobars of conserved regions are shown sequentially. The columns with full information bits are the conserved ones, and those with partial information bits are variable. Click here for file

Additional file 9 **Accessible regions and their mappings to conserved regions**. All accessible regions that were generated using SFOLD (as elaborated in the methods) were tabulated in sheet 1, and their mapping on the conserved regions is tabulated in sheet 2. Click here for file
