# Supplementary material for: Identification of novel conserved functional motifs across most Influenza A viral strains
Source: Virol J. 2011 Jan 27;8:44. doi: 10.1186/1743-422X-8-44 (PMC3036627; doi:10.1186/1743-422X-8-44)
Supplement: Additional file 7 — Conserved regions in the Influenza A viral segments. The position, length, conservation percent of each conserved region is shown. [file 1743-422X-8-44-S7.DOCX]

**Conserved regions in the influenza genome.**

Each segment and the position and size (length) of the conserved region is represented. The conservation percentage of each conserved region is also shown.

| **Segment** | **Conserved Region** | **Length** | **Conservation percentage** |
| --- | --- | --- | --- |
| **PB2** | PB2-1: Position 75 to 160 | 86 | 95.44 |
|  | PB2-2: Position 183 to 238 | 56 | 93.93 |
|  | PB2-3: Position 496 to 518 | 23 | 93.35 |
|  | PB2-4: Position 655 to 675 | 21 | 93.84 |
|  | PB2-5: Position 923 to 952 | 30 | 94.59 |
|  | PB2-6: Position 960 to 994 | 35 | 96.29 |
|  | PB2-7: Position 1270 to 1290 | 21 | 95.46 |
|  | PB2-8: Position 1648 to 1685 | 38 | 93.87 |
|  | PB2-9: Position 1703 to 1723 | 21 | 94.23 |
|  | PB2-10: Position 1958 to 1980 | 23 | 94.46 |
|  | PB2-11: Position 1982 to 2005 | 24 | 92.96 |
|  | PB2-12: Position 2165 to 2317 | 153 | 96.73 |
| **PB1** | PB1-1: Position 101 to 216 | 116 | 96.41 |
|  | PB1-2: Position 230 to 493 | 264 | 95.10 |
|  | PB1-3: Position 521 to 628 | 108 | 94.15 |
|  | PB1-4: Position 630 to 654 | 25 | 94.14 |
|  | PB1-5: Position 659 to 681 | 23 | 92.73 |
|  | PB1-6: Position 722 to 744 | 23 | 93.58 |
|  | PB1-7: Position 770 to 820 | 51 | 96.19 |
|  | PB1-8: Position 881 to 946 | 66 | 95.48 |
|  | PB1-9: Position 1073 to 1188 | 116 | 92.49 |
|  | PB1-10: Position 1210 to 1326 | 117 | 93.73 |
|  | PB1-11: Position 1340 to 1398 | 59 | 95.44 |
|  | PB1-12: Position 1403 to 1455 | 53 | 93.87 |
|  | PB1-13: Position 1469 to 1506 | 38 | 93.78 |
|  | PB1-14: Position 1508 to 1566 | 59 | 94.59 |
|  | PB1-15: Position 1568 to 1599 | 32 | 92.72 |
|  | PB1-16: Position 1616 to 1669 | 54 | 94.39 |
|  | PB1-17: Position 1671 to 1698 | 28 | 93.05 |
|  | PB1-18: Position 1700 to 1725 | 26 | 93.29 |
|  | PB1-19: Position 1766 to 1791 | 26 | 94.09 |
|  | PB1-20: Position 1826 to 1869 | 44 | 95.10 |
|  | PB1-21: Position 1970 to 2007 | 38 | 92.15 |
|  | PB1-22: Position 2012 to 2064 | 53 | 98.06 |
|  | PB1-23: Position 2165 to 2280 | 116 | 94.84 |
|  | PB1-24: Position 2282 to 2307 | 26 | 94.12 |
|  | PB1-25: Position 2310 to 2400 | 91 | 96.67 |
| **PA** | PA-1: Position 110 to 141 | 32 | 95.00 |
|  | PA-2: Position 315 to 352 | 38 | 95.15 |
|  | PA-3: Position 406 to 433 | 28 | 95.18 |
|  | PA-4: Position 486 to 547 | 62 | 94.04 |
|  | PA-5: Position 576 to 610 | 35 | 94.28 |
|  | PA-6: Position 621 to 677 | 57 | 98.16 |
|  | PA-7: Position 690 to 779 | 90 | 97.18 |
|  | PA-8: Position 784 to 838 | 55 | 95.39 |
|  | PA-9: Position 978 to 1002 | 25 | 95.65 |
|  | PA-10: Position 1178 to 1213 | 36 | 92.70 |
|  | PA-11: Position 1377 to 1428 | 52 | 94.35 |
|  | PA-12: Position 1439 to 1461 | 23 | 96.73 |
|  | PA-13: Position 1522 to 1556 | 35 | 94.81 |
|  | PA-14: Position 1576 to 1613 | 38 | 94.81 |
|  | PA-15: Position 1750 to 1772 | 23 | 95.05 |
|  | PA-16: Position 1792 to 1824 | 33 | 94.01 |
|  | PA-17: Position 1876 to 1920 | 45 | 94.90 |
|  | PA-18: Position 2083 to 2155 | 73 | 95.84 |
| **NP** | NP-1: Position 62 to 161 | 100 | 93.64 |
|  | NP-2: Position 188 to 226 | 39 | 94.64 |
|  | NP-3: Position 245 to 270 | 26 | 93.95 |
|  | NP-4: Position 296 to 321 | 26 | 95.44 |
|  | NP-5: Position 537 to 580 | 44 | 94.80 |
|  | NP-6: Position 675 to 698 | 24 | 95.71 |
|  | NP-7: Position 719 to 804 | 86 | 94.09 |
|  | NP-8: Position 894 to 916 | 23 | 92.40 |
|  | NP-9: Position 1026 to 1046 | 21 | 94.39 |
|  | NP-10: Position 1053 to 1087 | 35 | 93.66 |
|  | NP-11: Position 1159 to 1184 | 26 | 94.81 |
|  | NP-12: Position 1399 to 1445 | 47 | 93.36 |
|  | NP-13: Position 1447 to 1486 | 40 | 97.08 |
|  | NP-14: Position 1488 to 1527 | 40 | 94.70 |
| **M** | M-1: Position 64 to 232 | 169 | 95.40 |
|  | M-2: Position 235 to 394 | 160 | 94.60 |
|  | M-3: Position 405 to 427 | 23 | 94.31 |
|  | M-4: Position 431 to 451 | 21 | 94.42 |
|  | M-5: Position 494 to 520 | 27 | 94.08 |
|  | M-6: Position 599 to 663 | 65 | 97.22 |
|  | M-7: Position 665 to 702 | 38 | 94.16 |
|  | M-8: Position 733 to 1000 | 268 | 90.49 |
| **NS** | NS-1: Position 59 to 137 | 79 | 98.96 |
|  | NS-2: Position 188 to 225 | 38 | 95.01 |
|  | NS-3: Position 251 to 276 | 26 | 93.27 |
|  | NS-4: Position 318 to 432 | 115 | 96.87 |
|  | NS-5: Position 440 to 473 | 34 | 95.28 |
|  | NS-6: Position 492 to 700 | 209 | 92.95 |
|  | NS-7: Position 713 to 740 | 28 | 96.65 |
|  | NS-8: Position 743 to 836 | 94 | 95.07 |
|  | NS-9: Position 840 to 874 | 35 | 97.57 |
