# Supplementary figures and images for: Identification of novel conserved functional motifs across most Influenza A viral strains
Source: Virol J. 2011 Jan 27;8:44. doi: 10.1186/1743-422X-8-44 (PMC3036627; doi:10.1186/1743-422X-8-44)

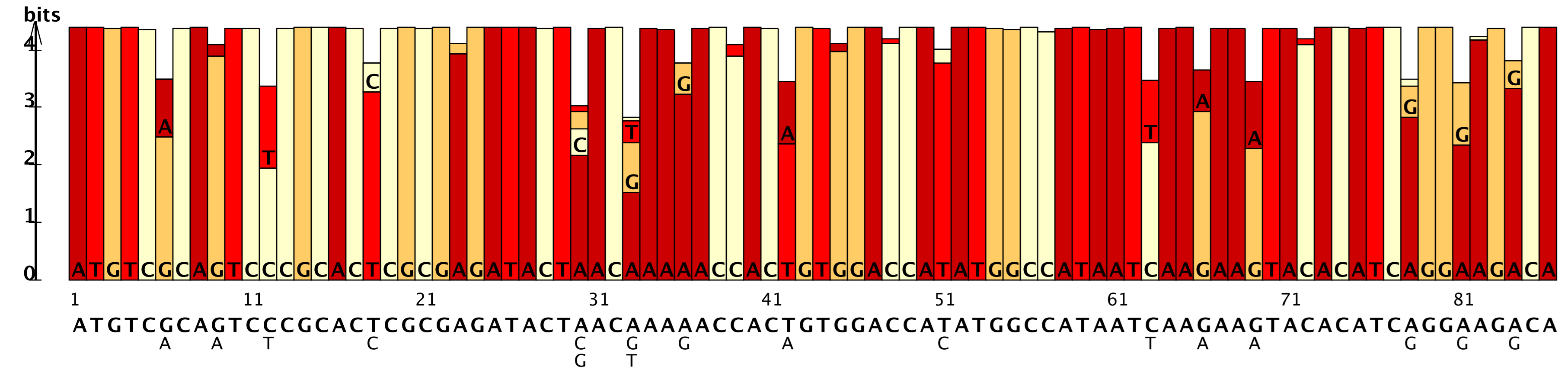

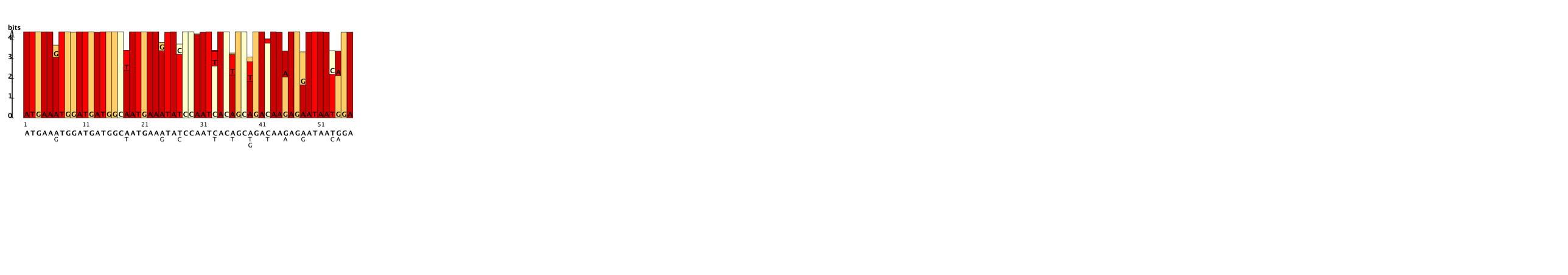

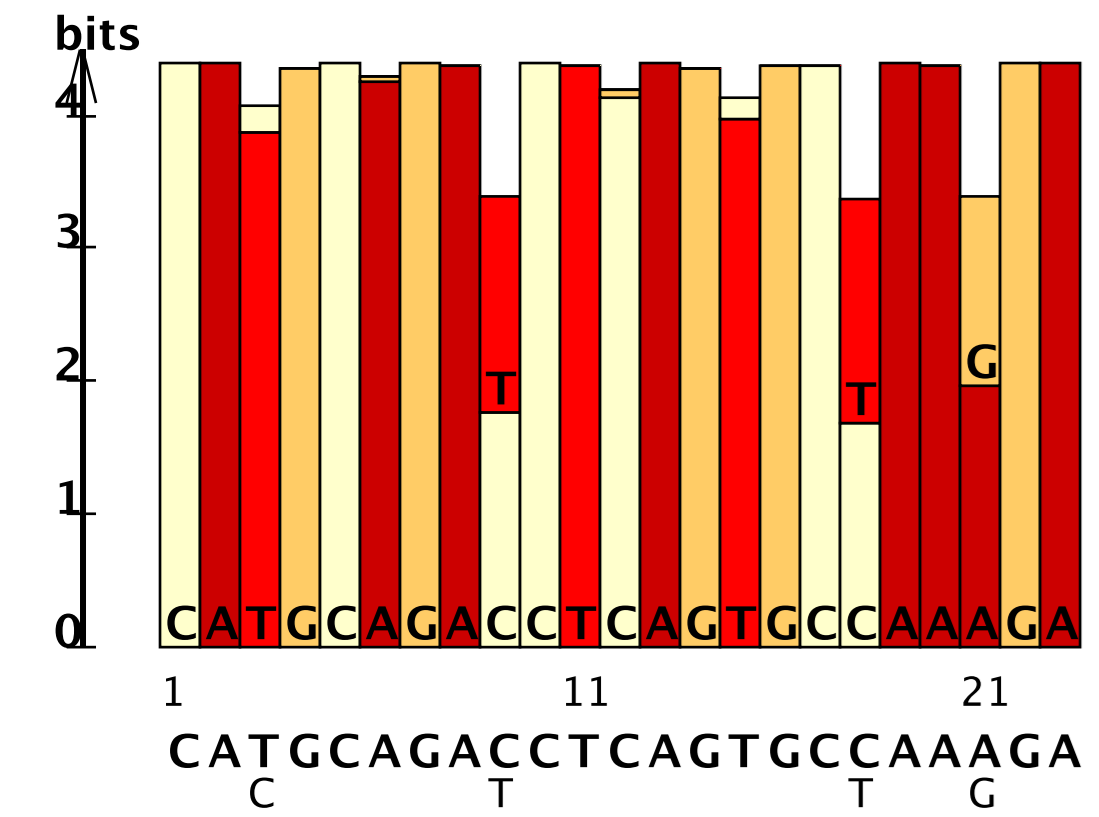

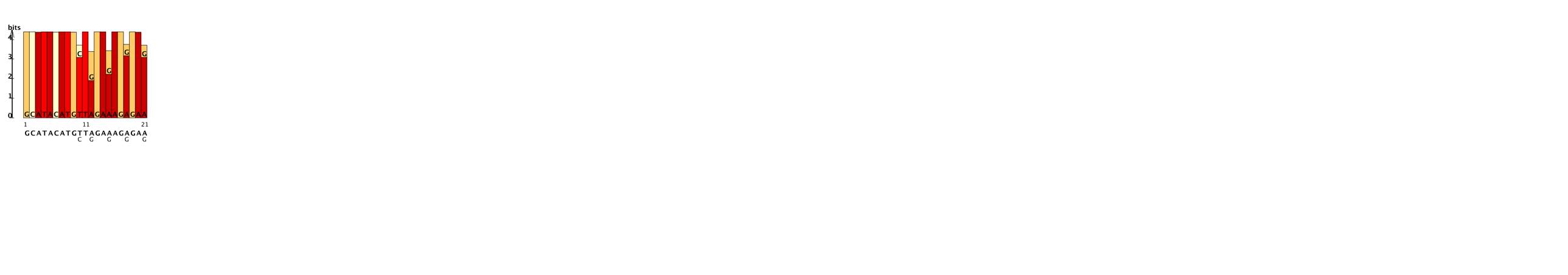

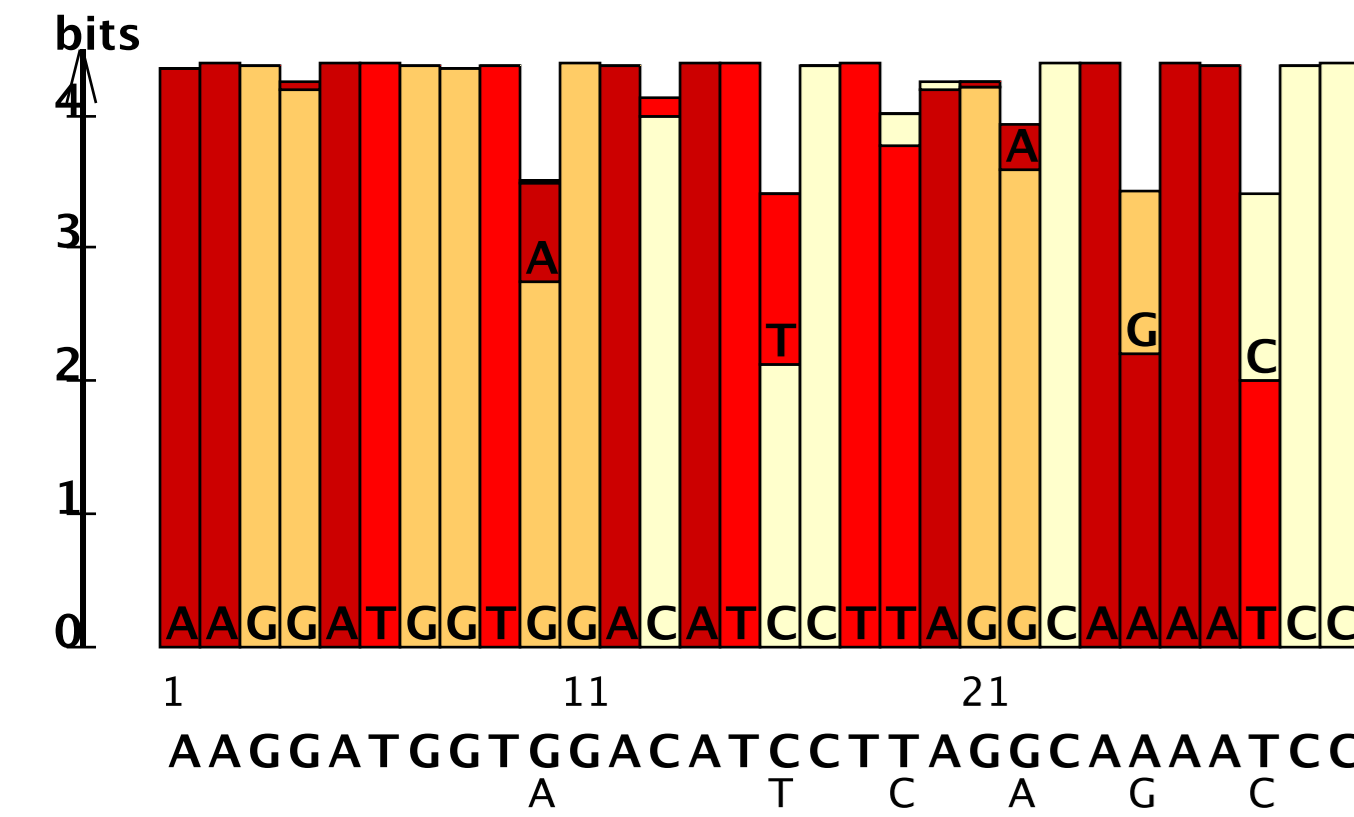

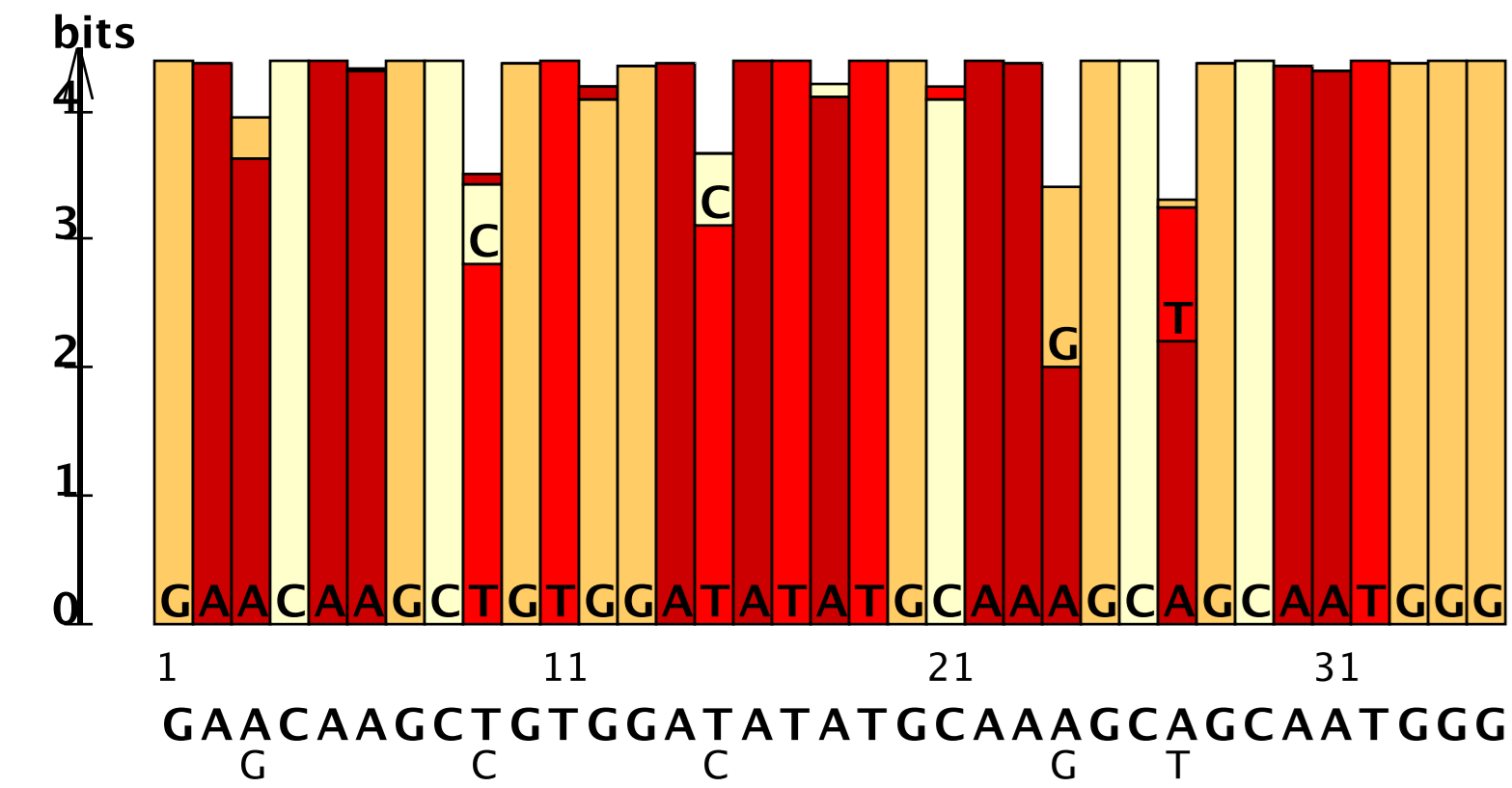

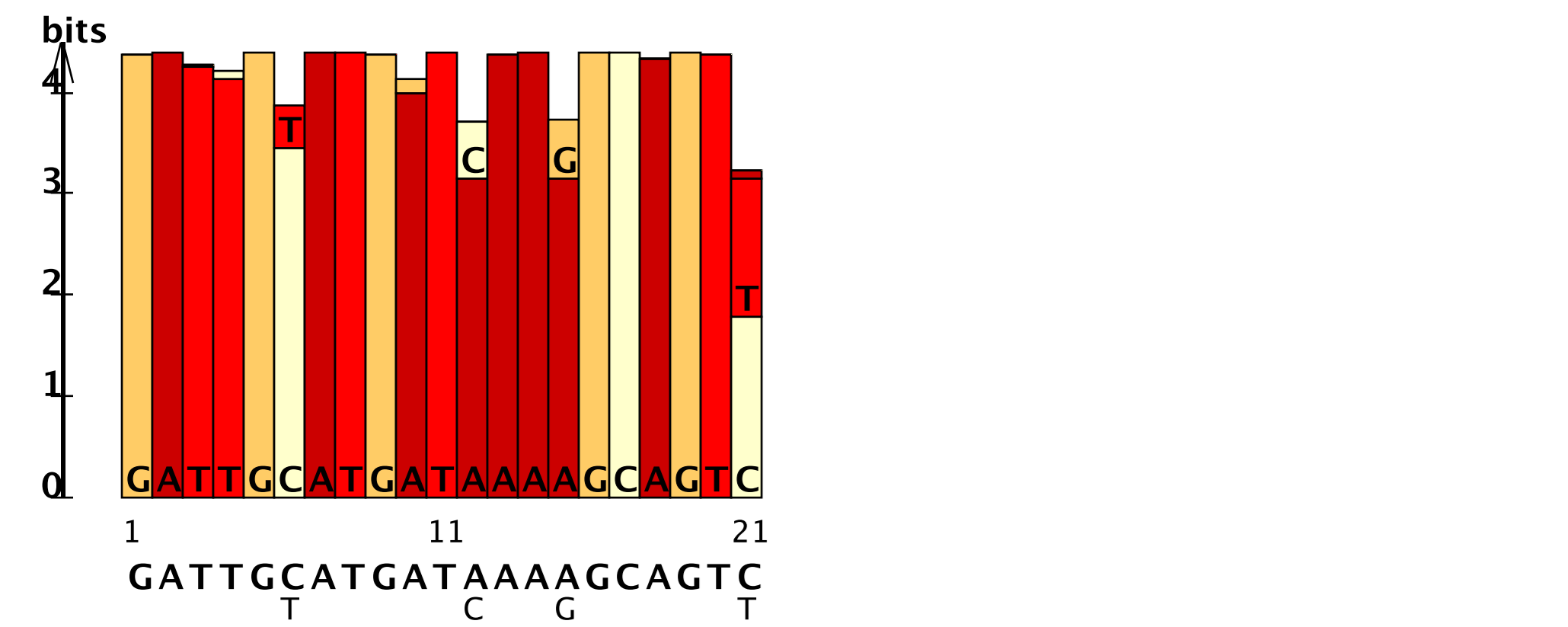

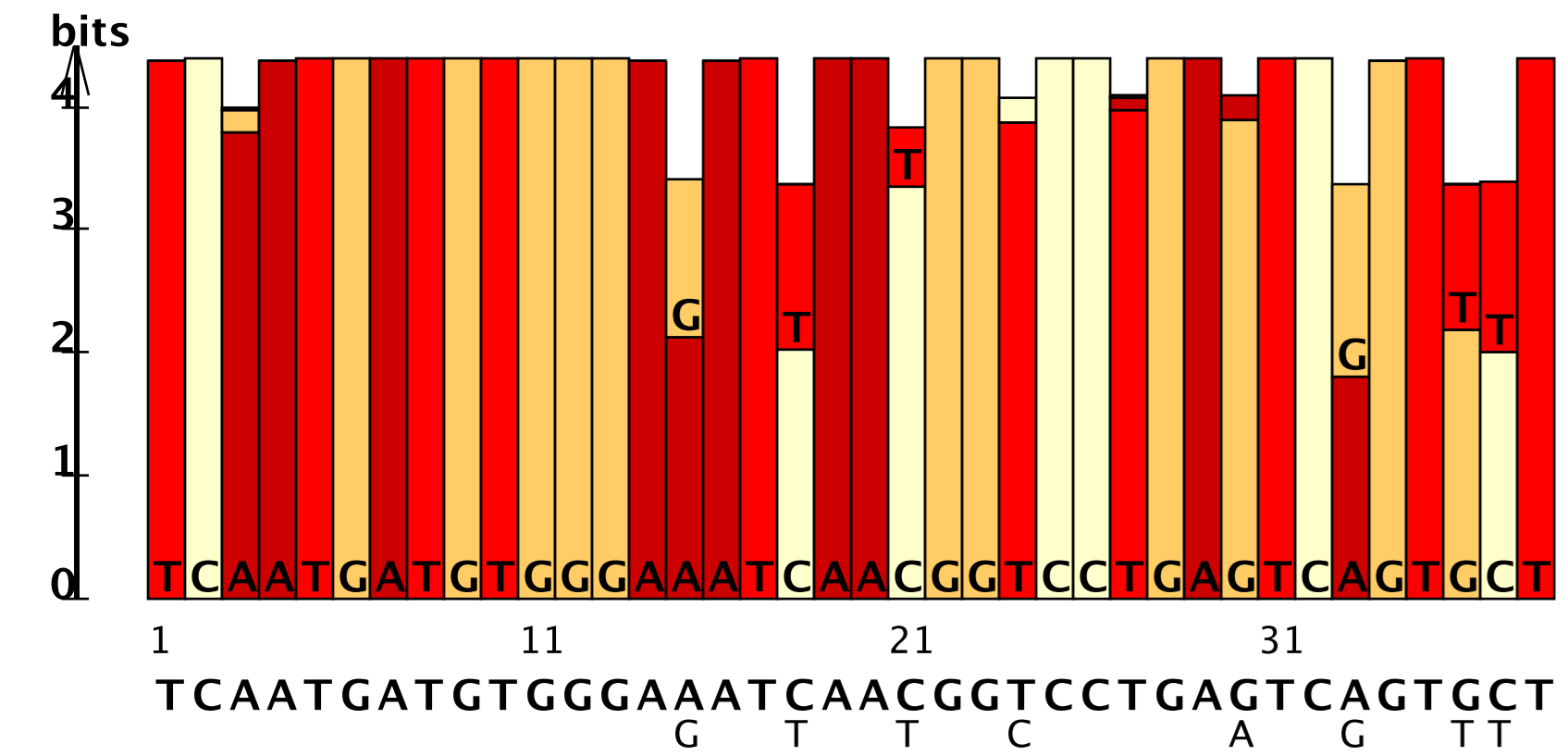

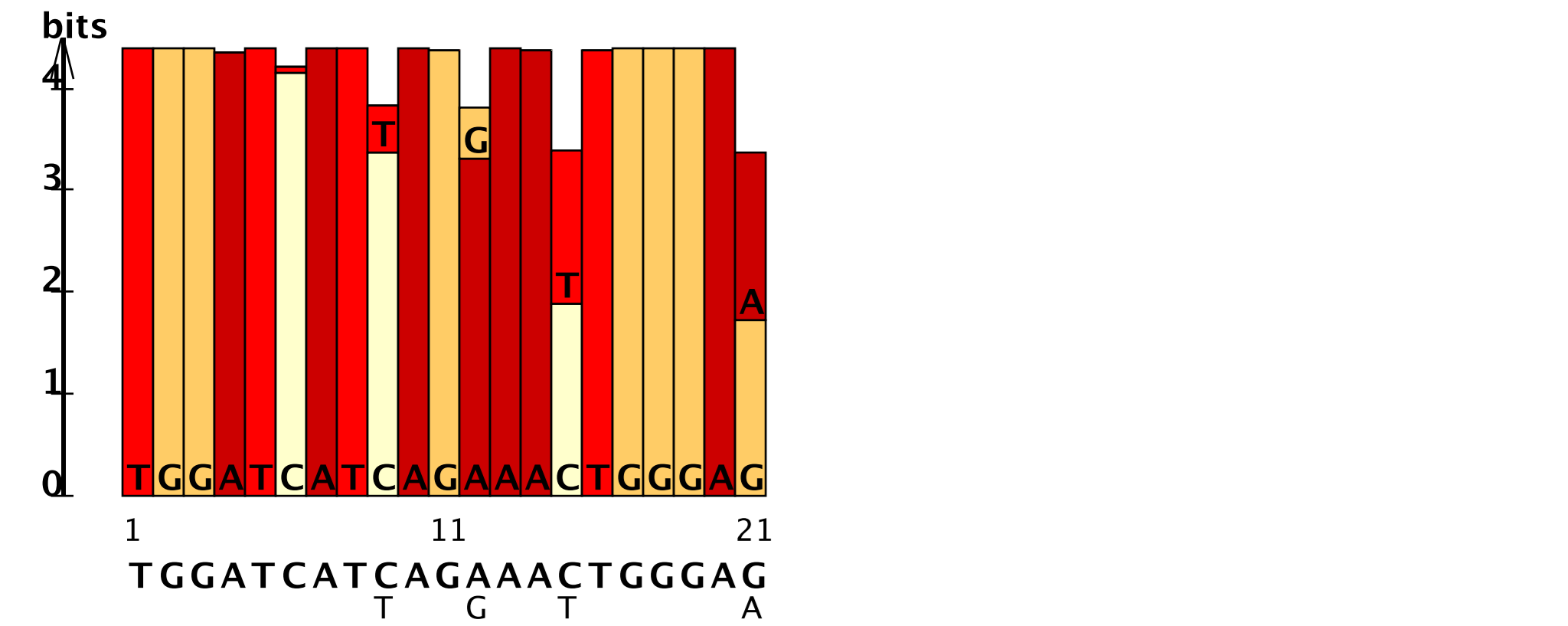

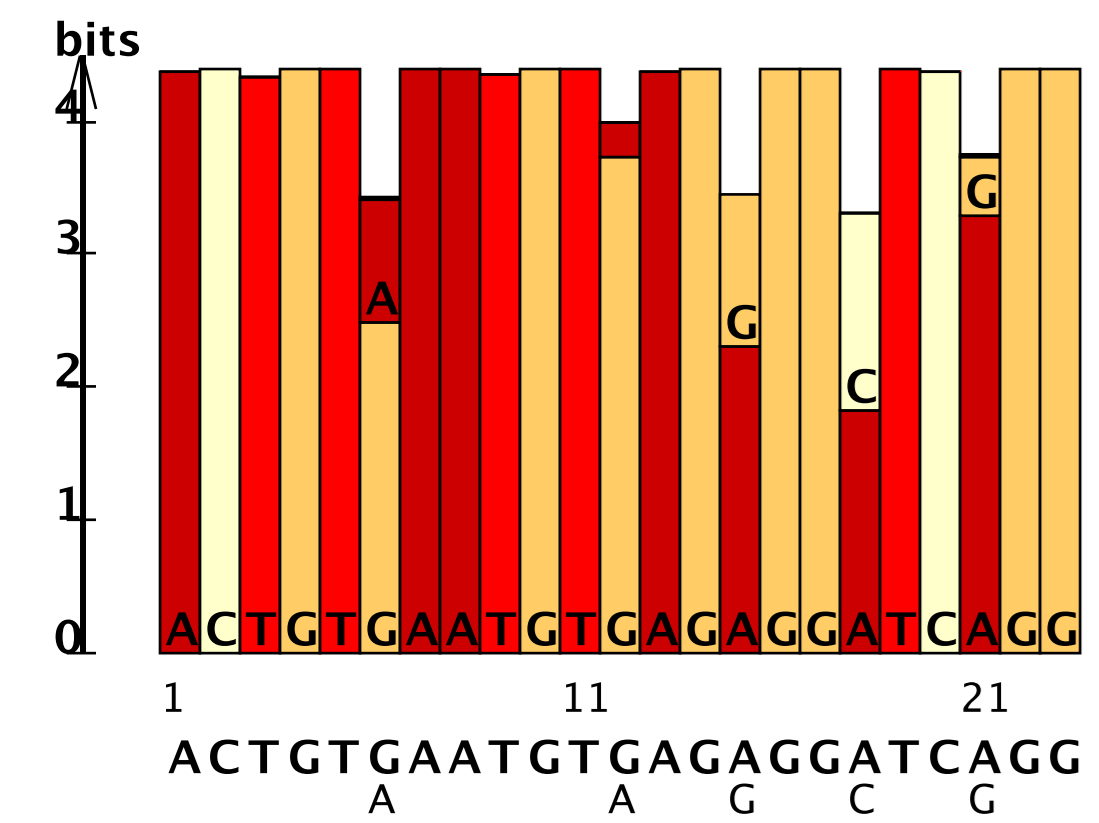

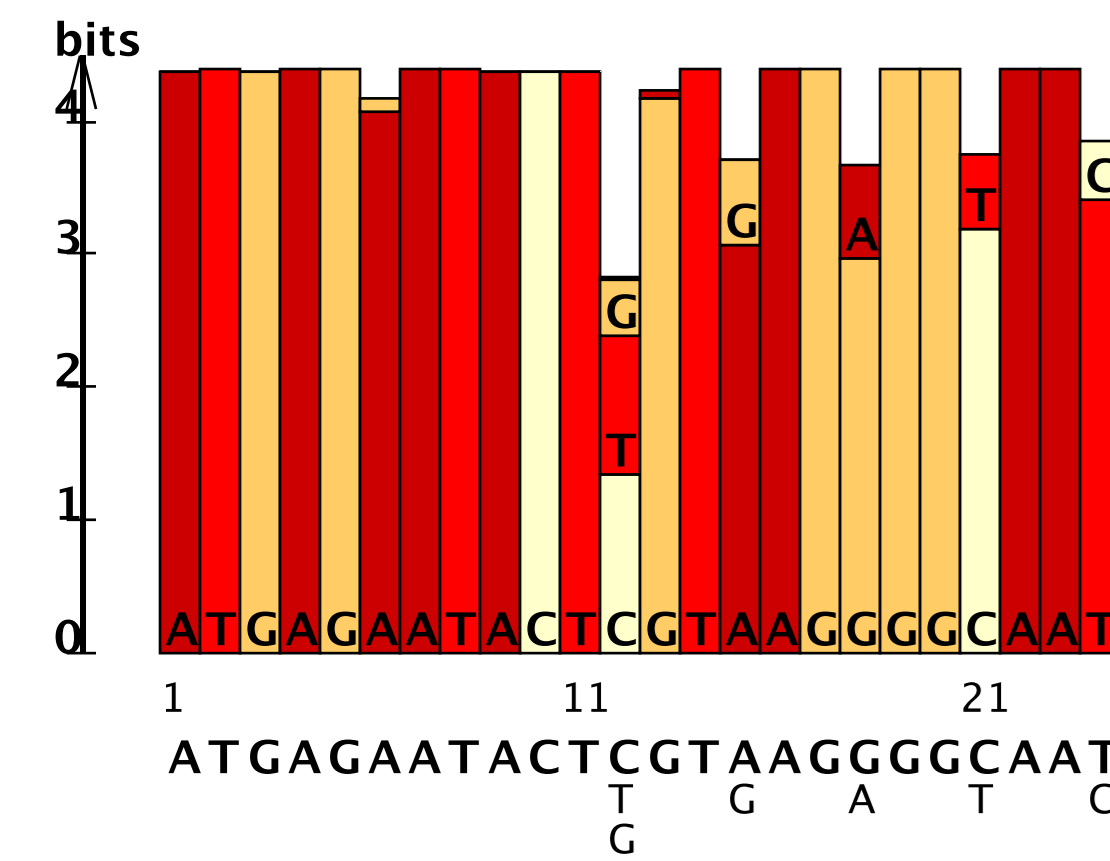

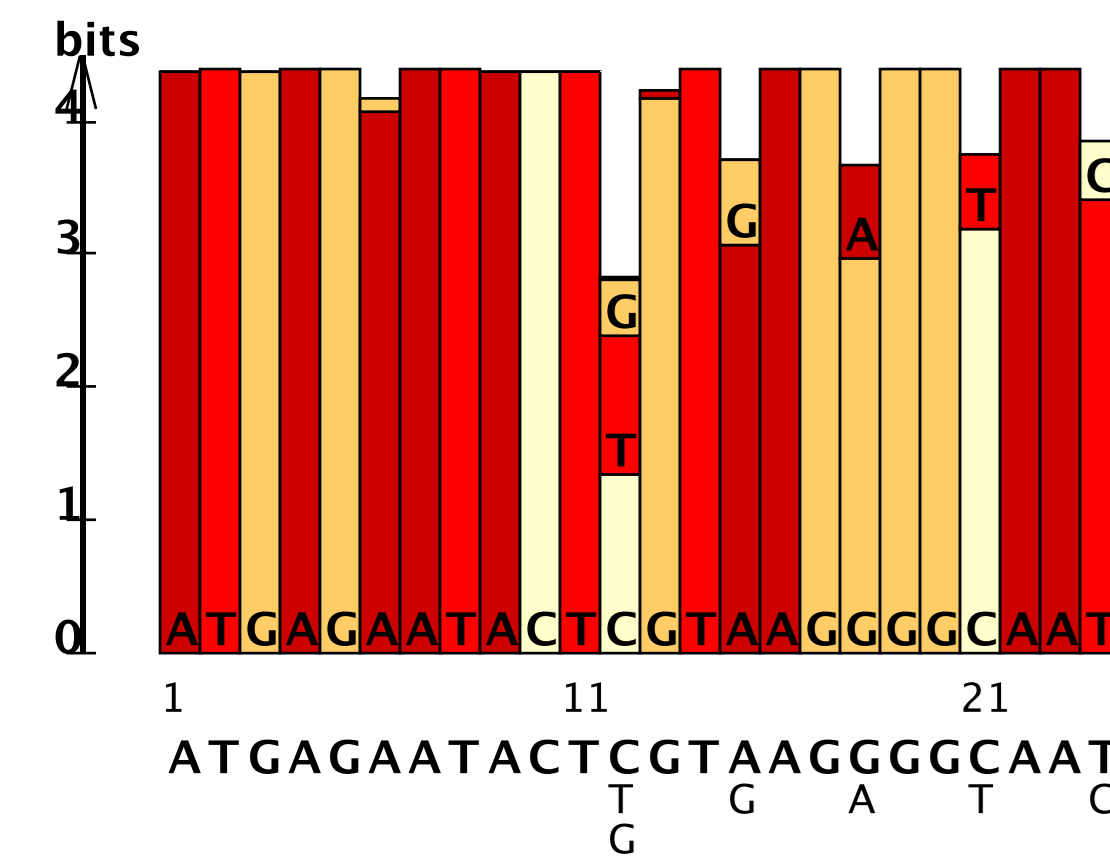

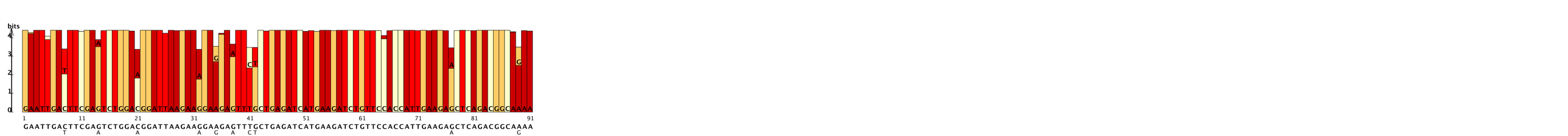

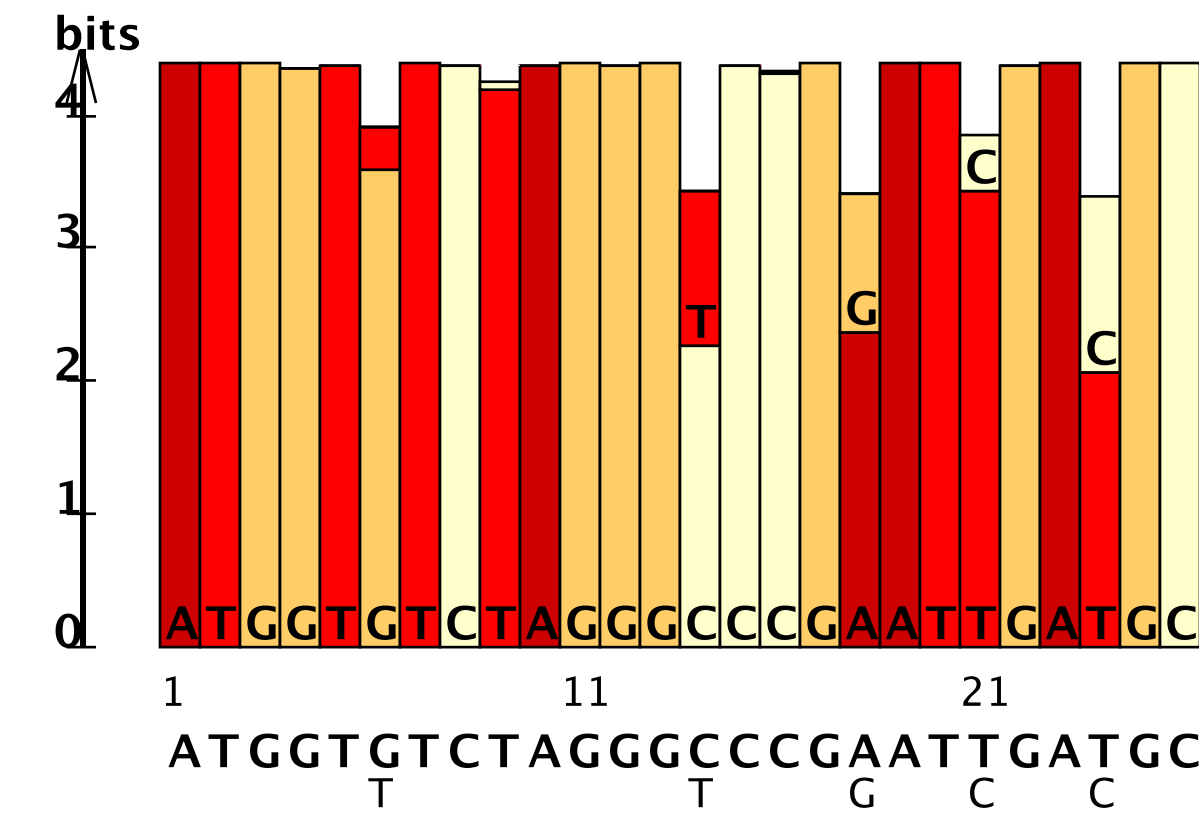

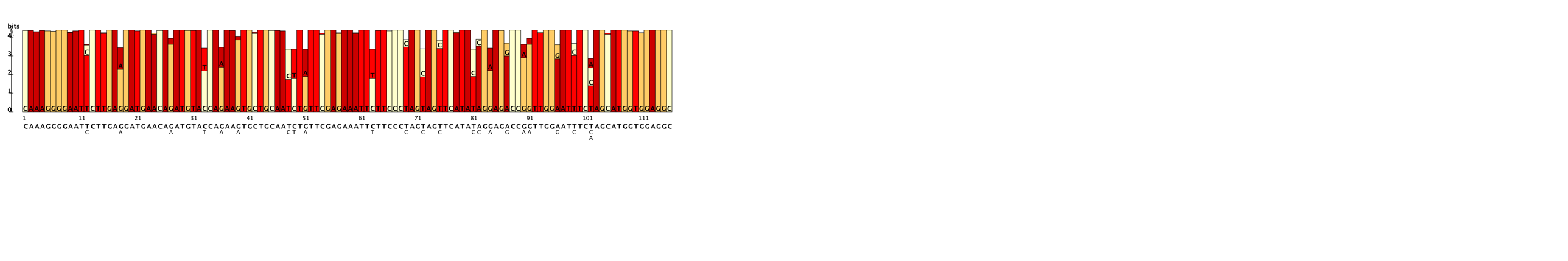

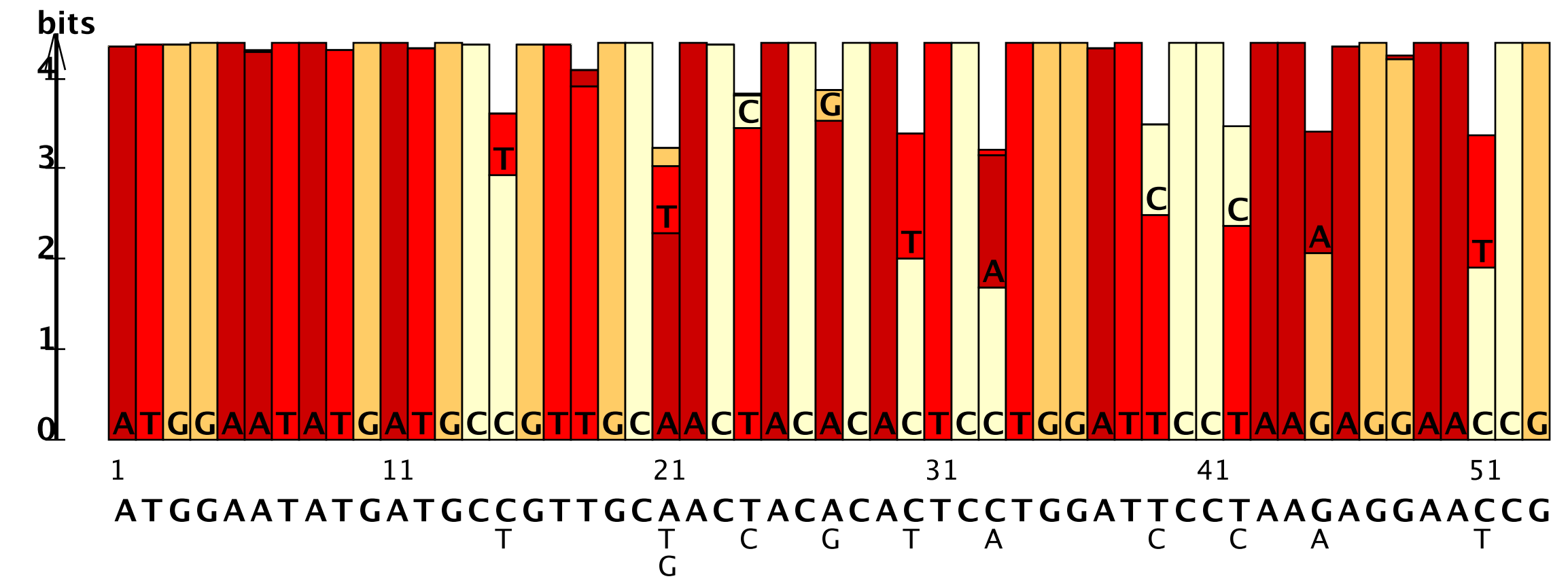

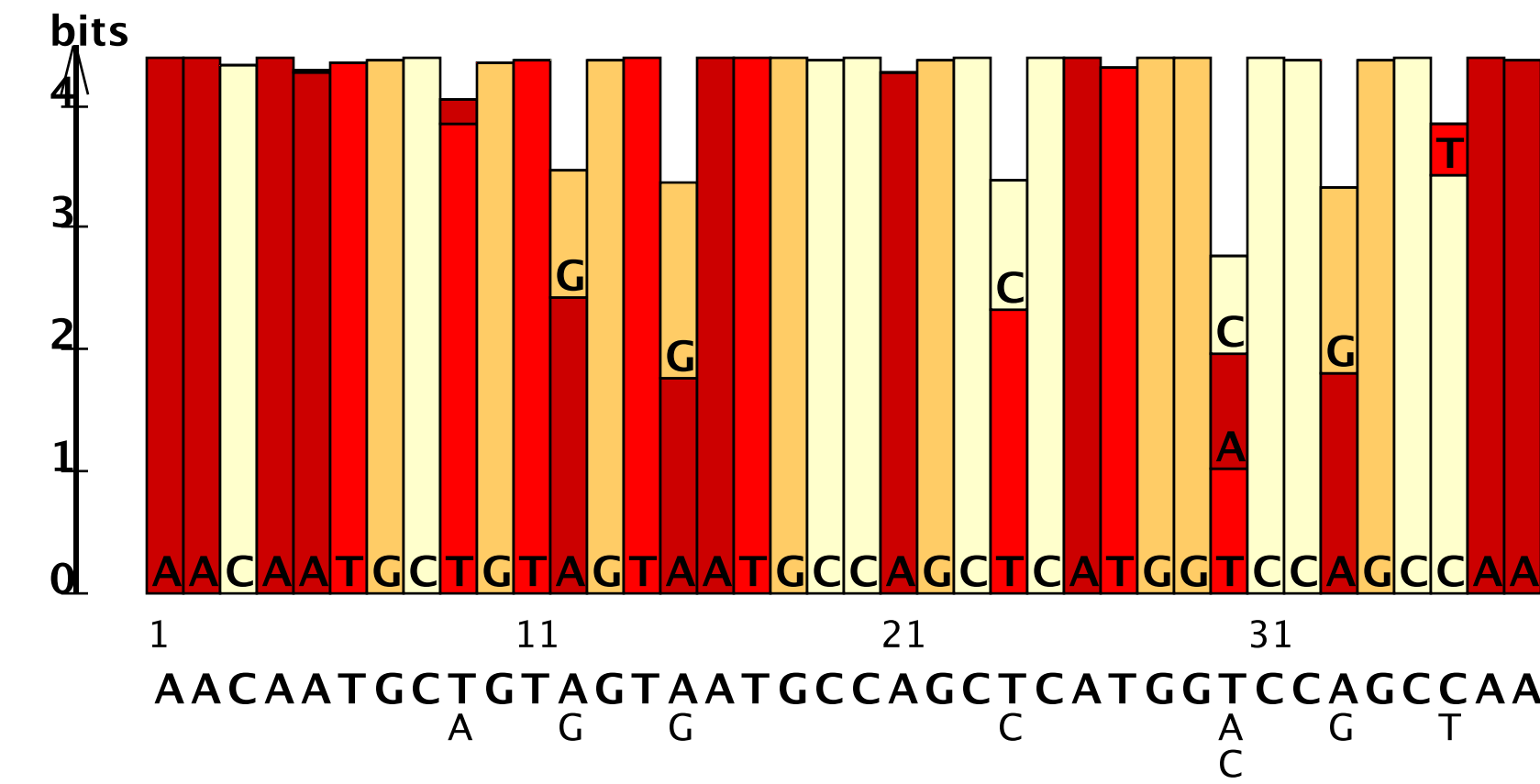

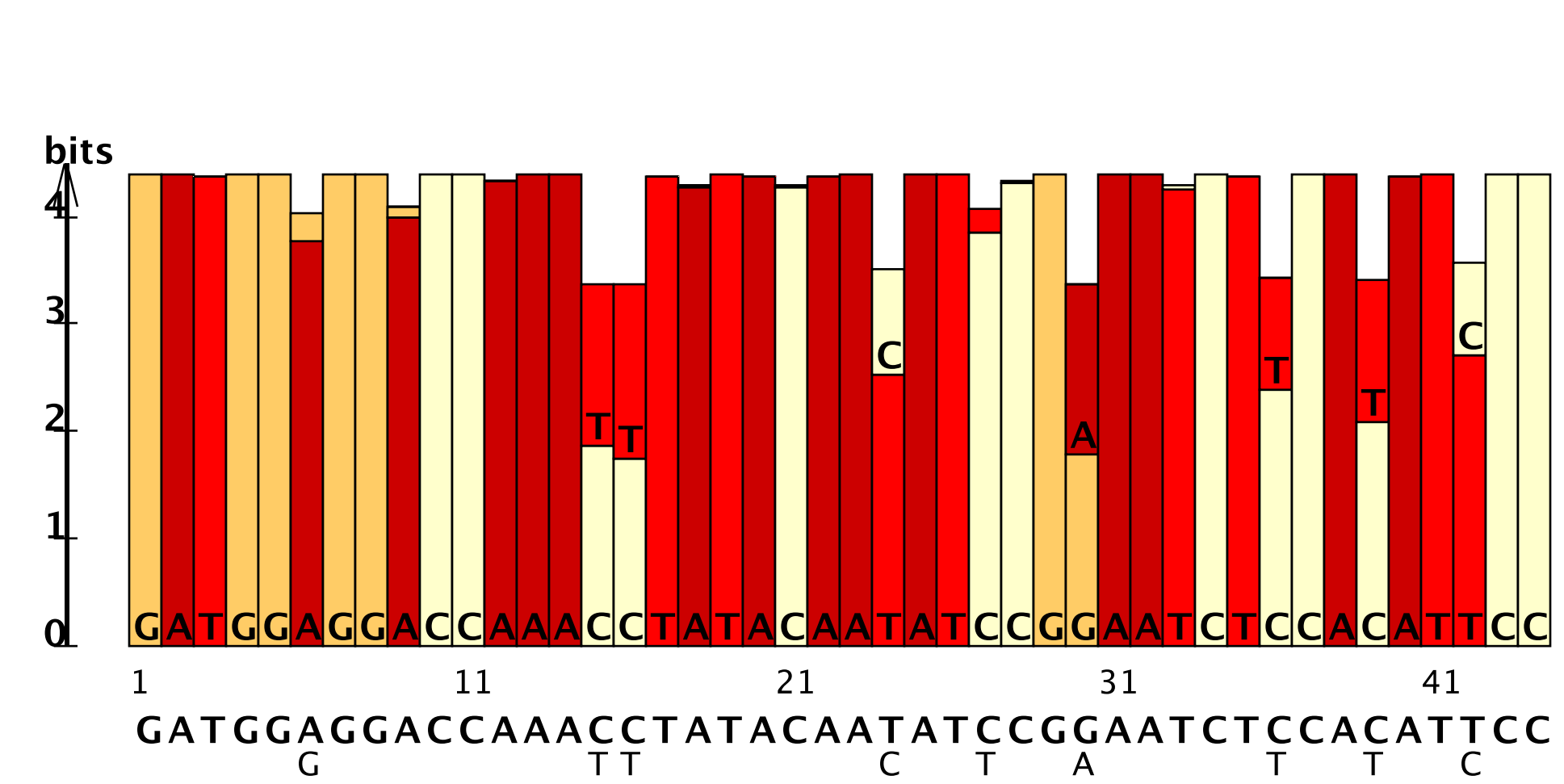

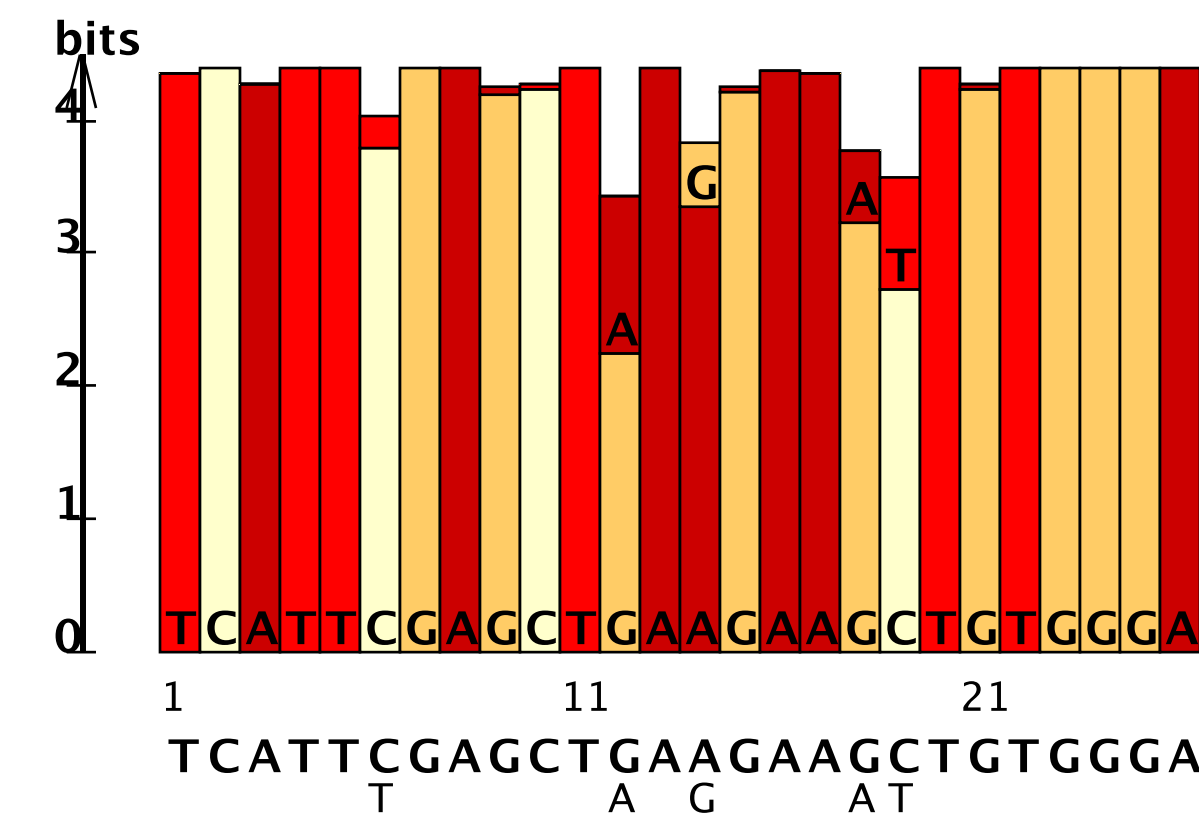

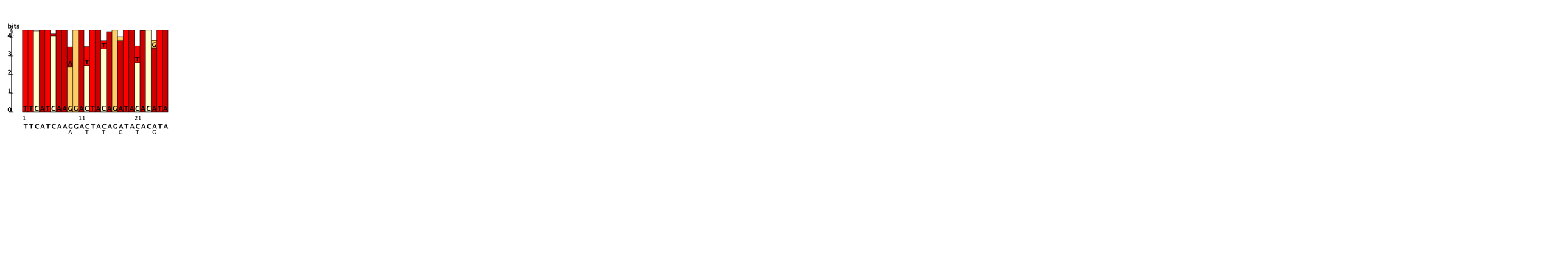

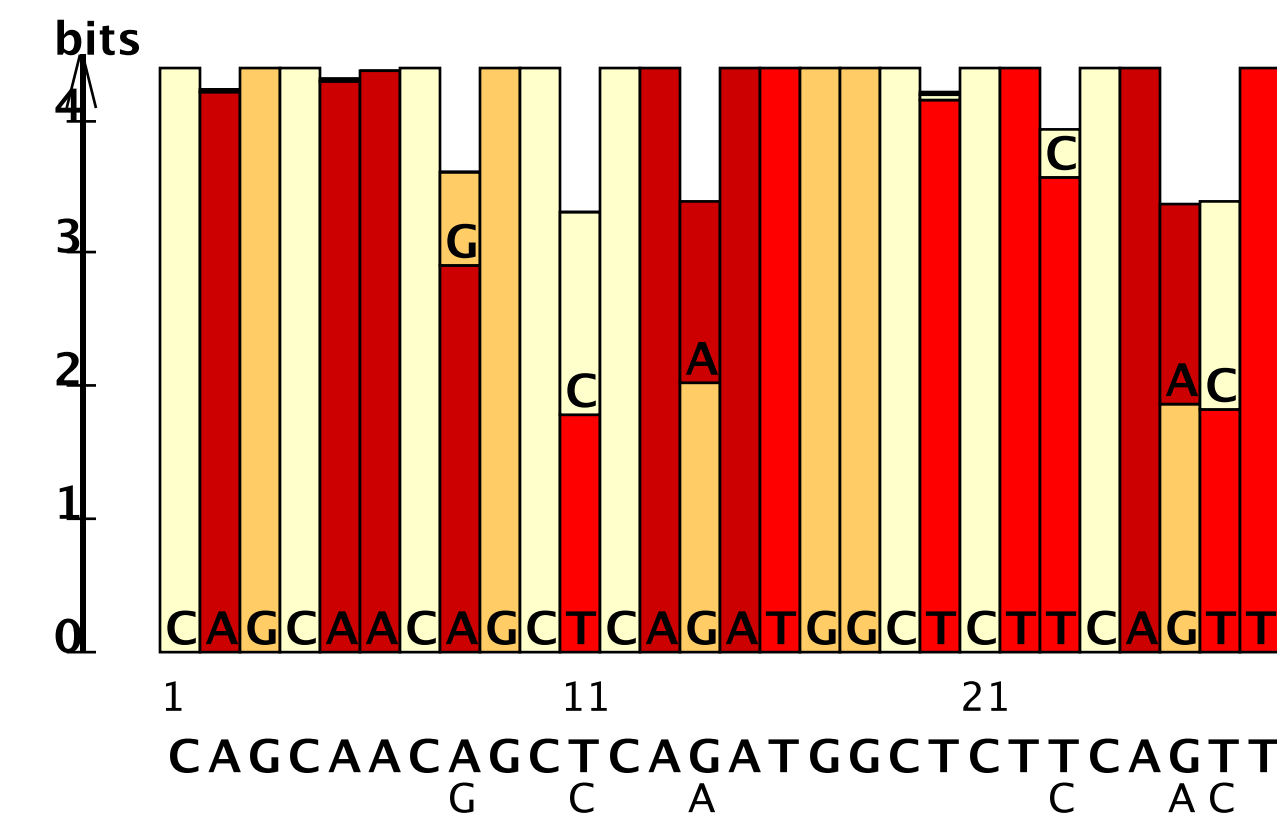

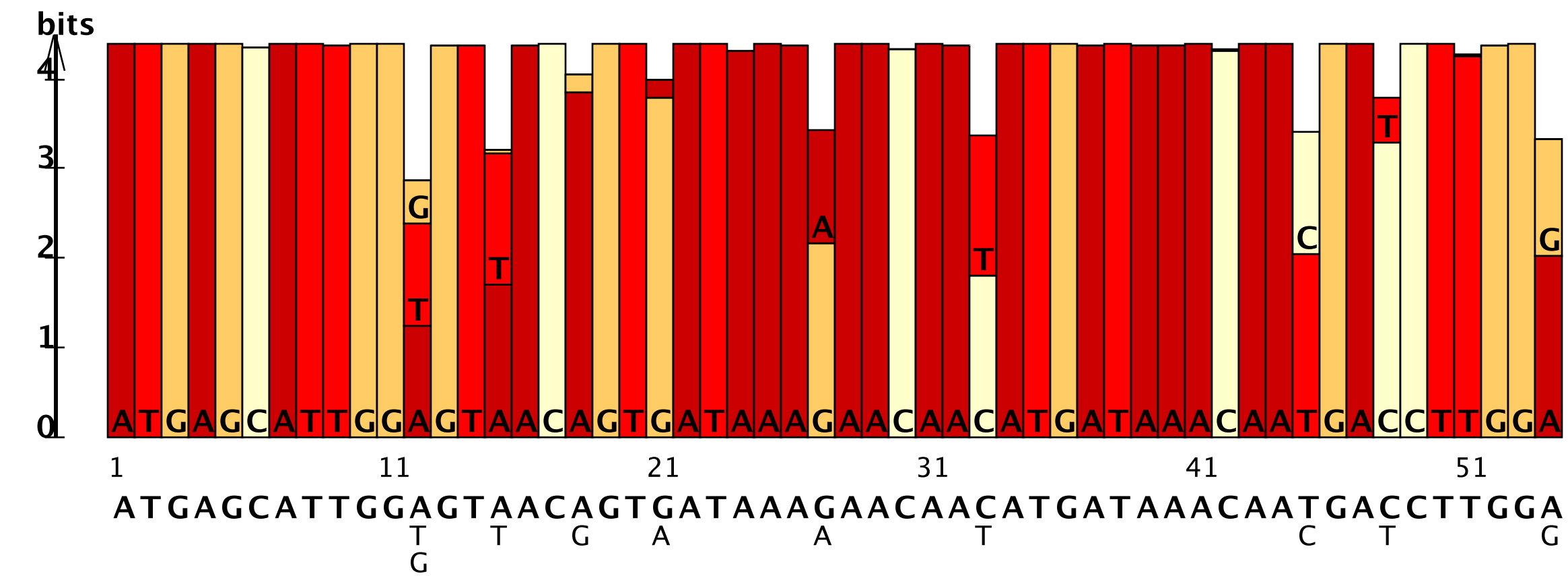

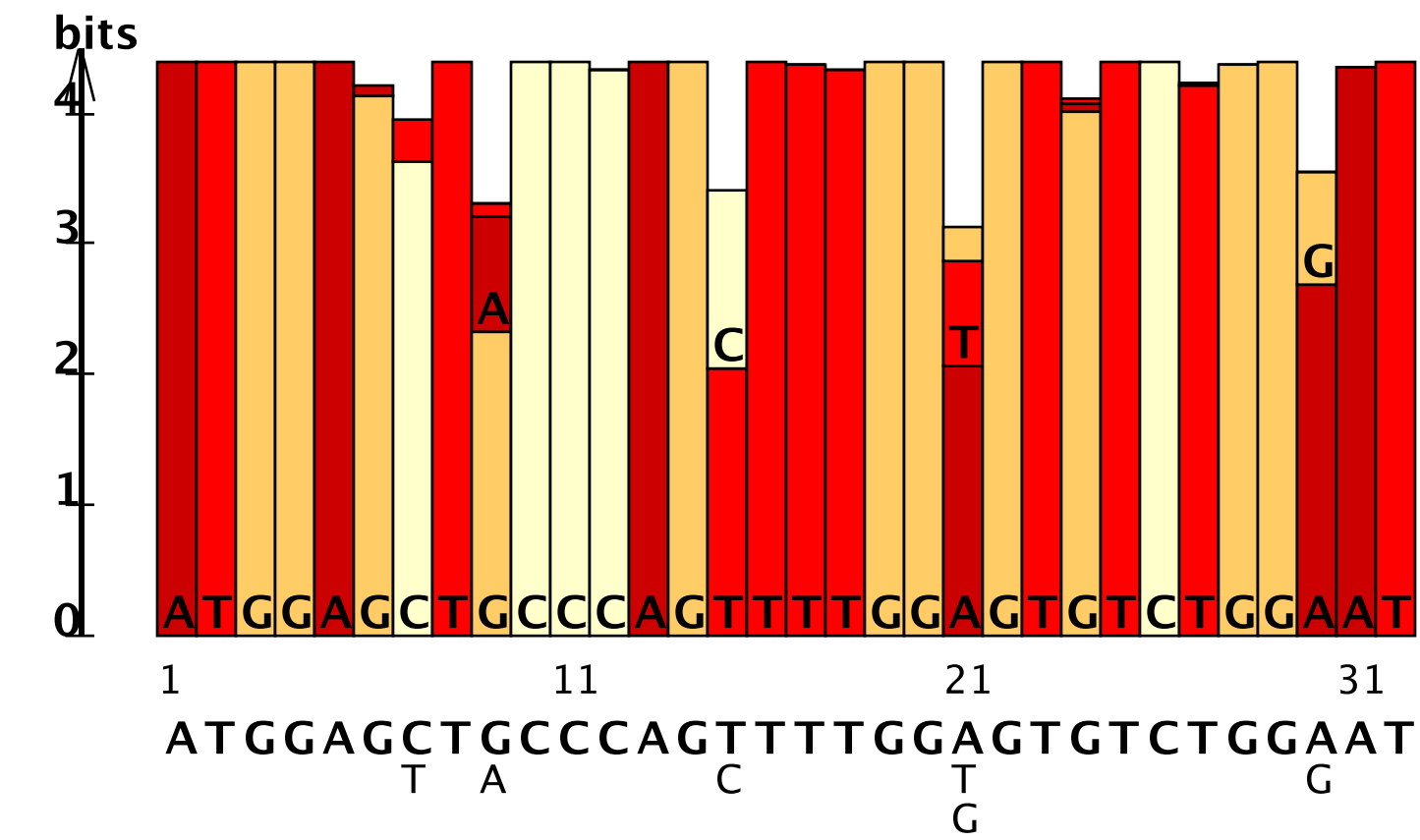

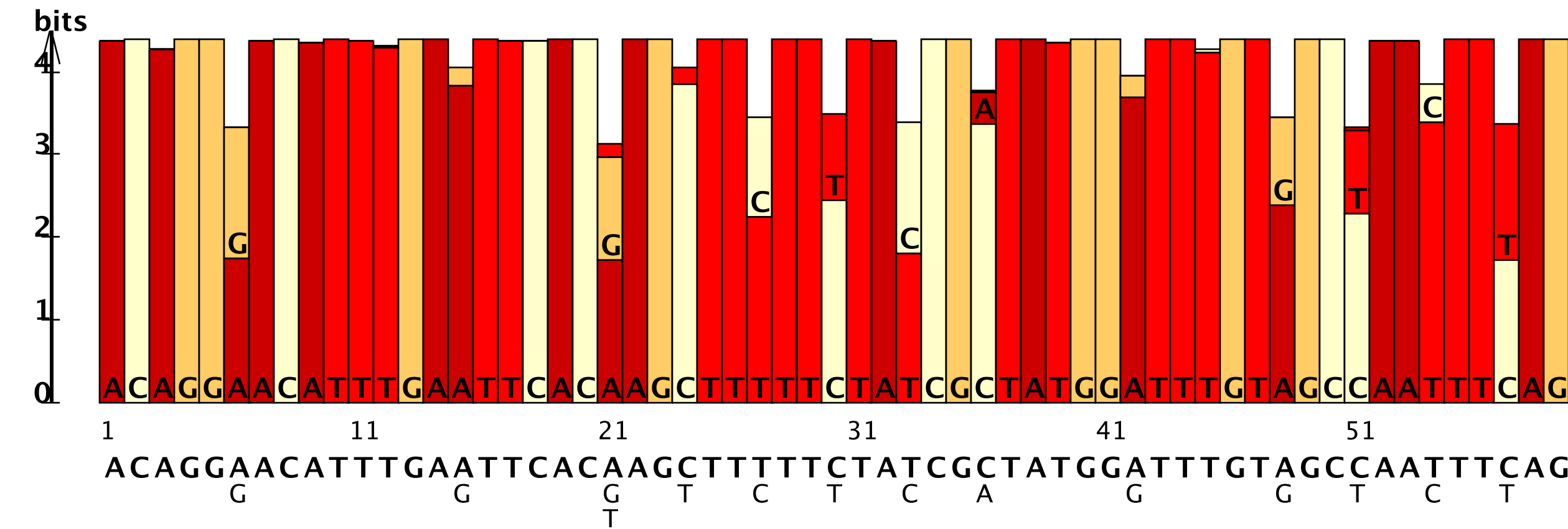

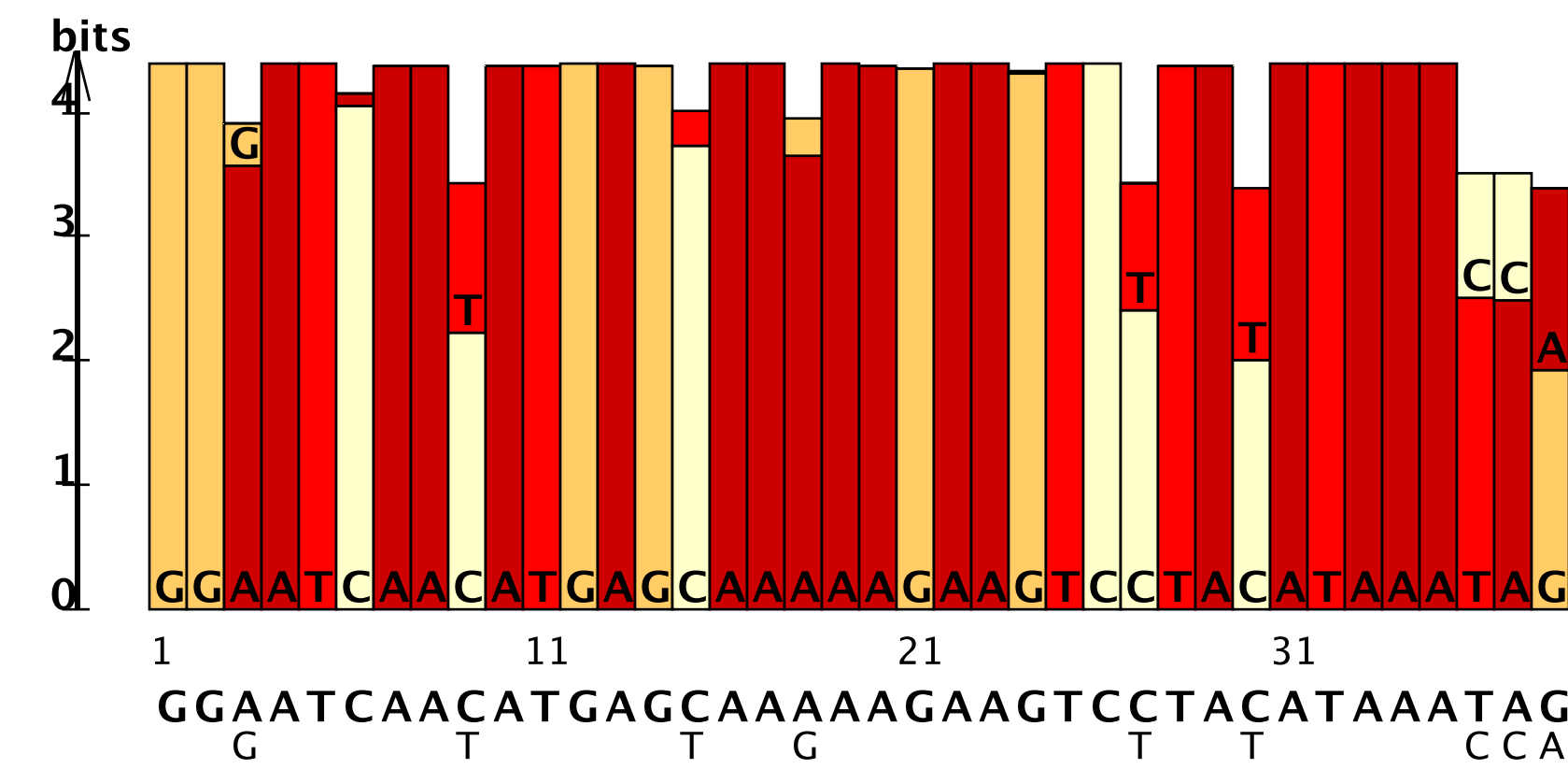

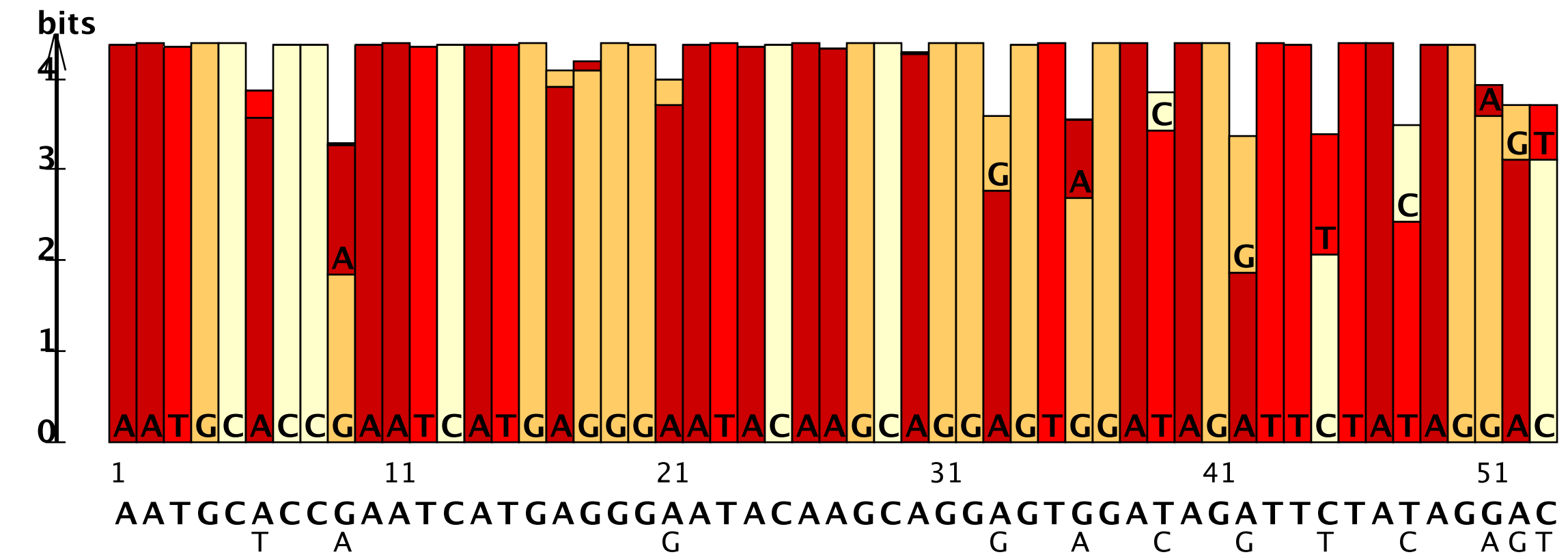

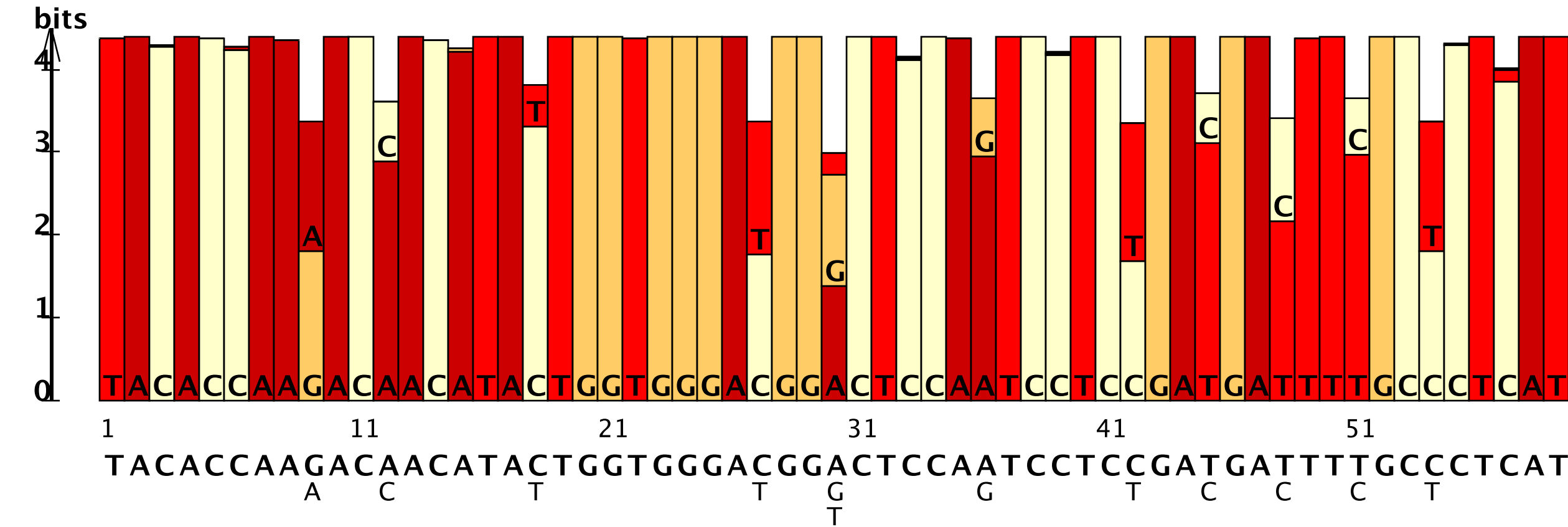

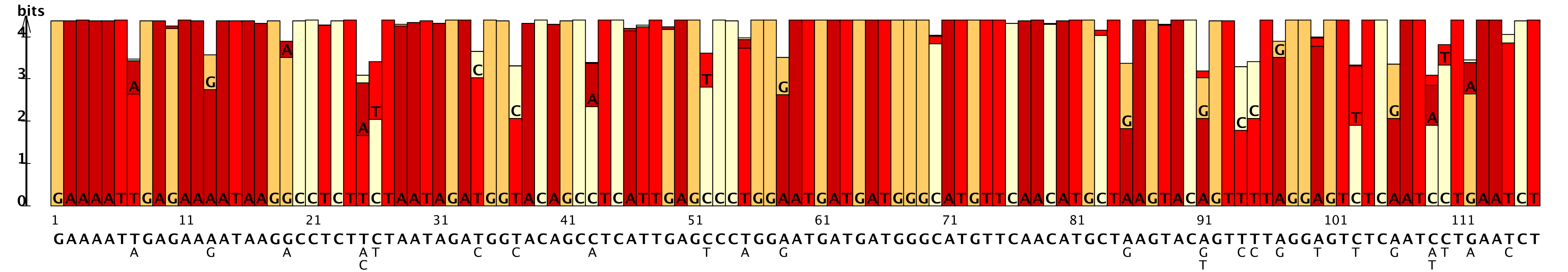

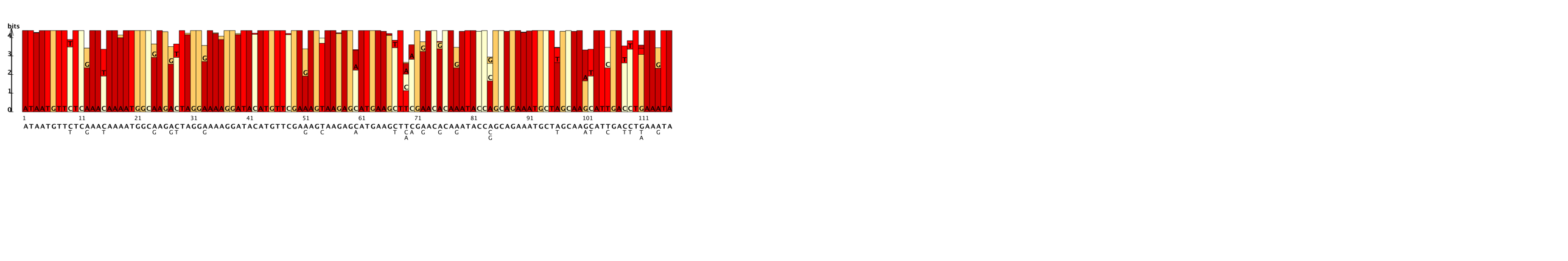

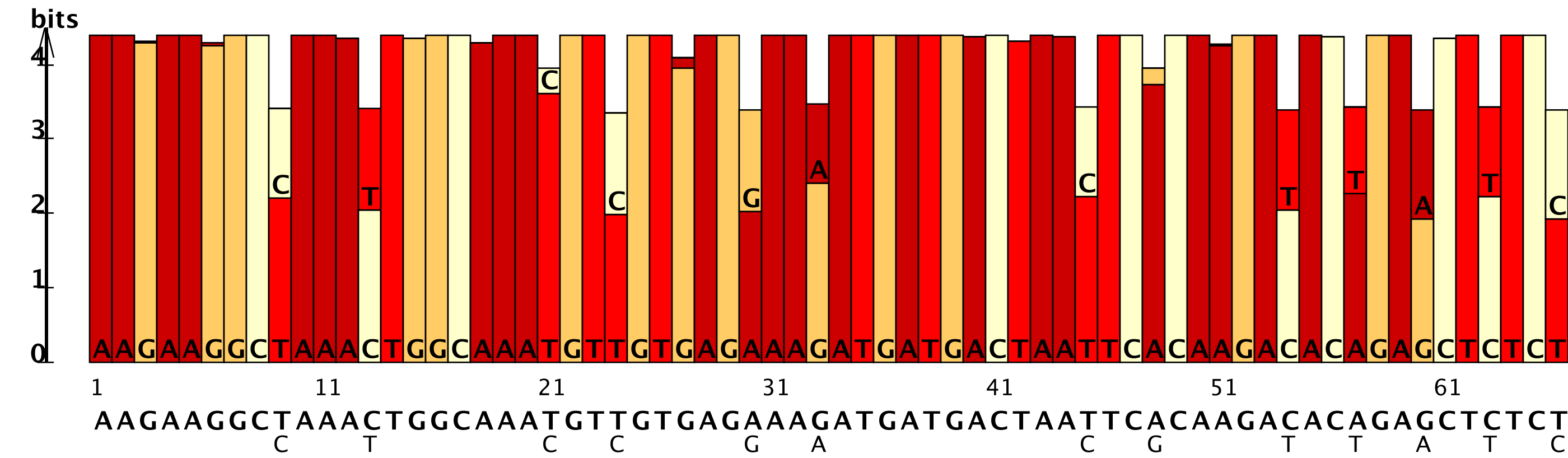

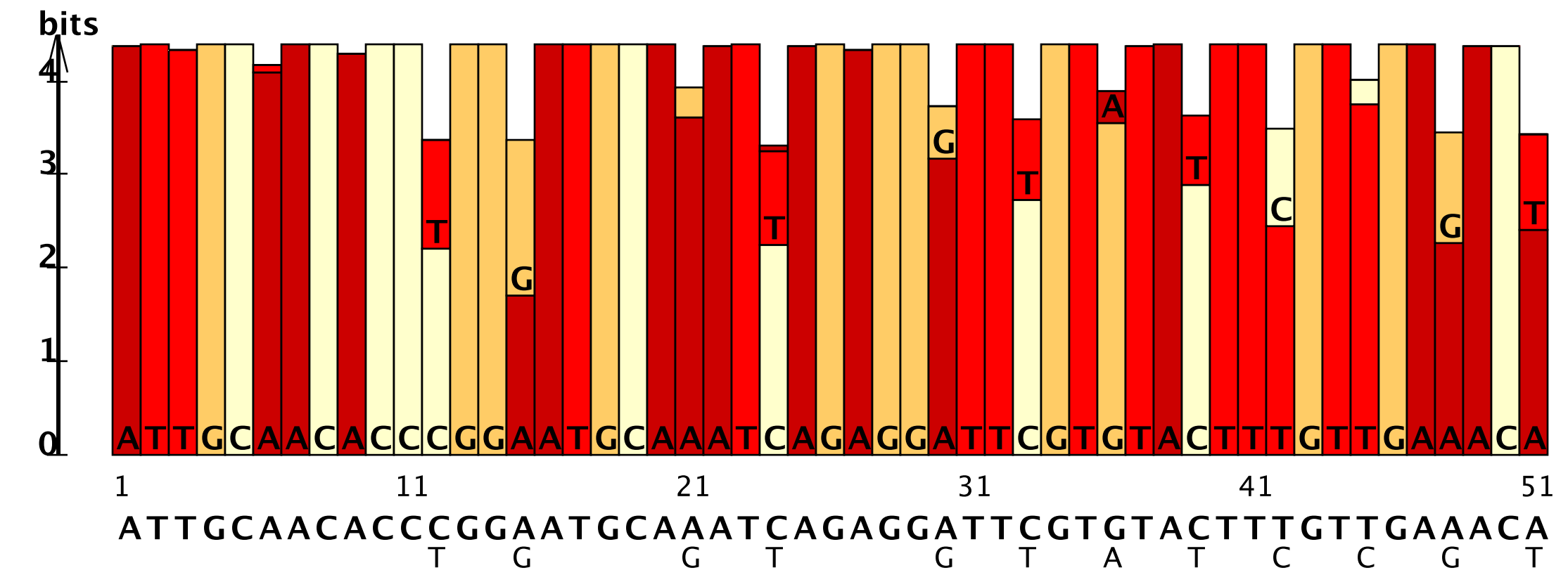

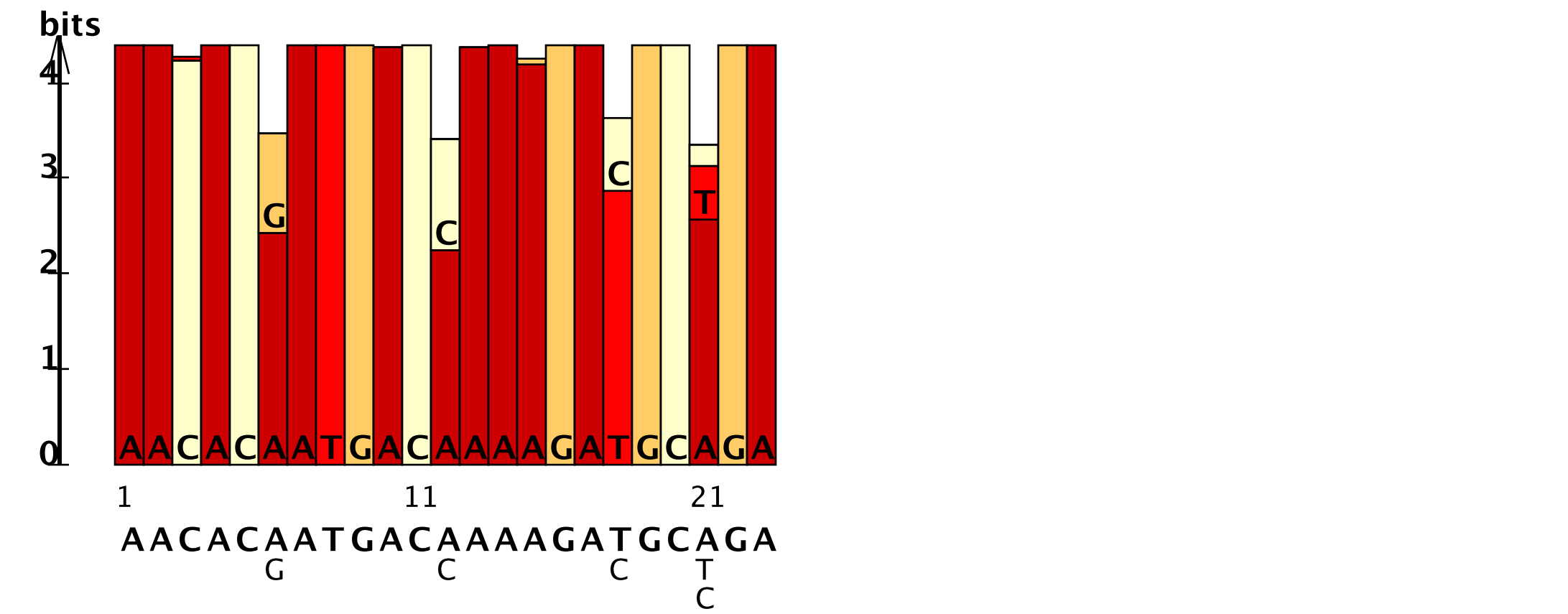

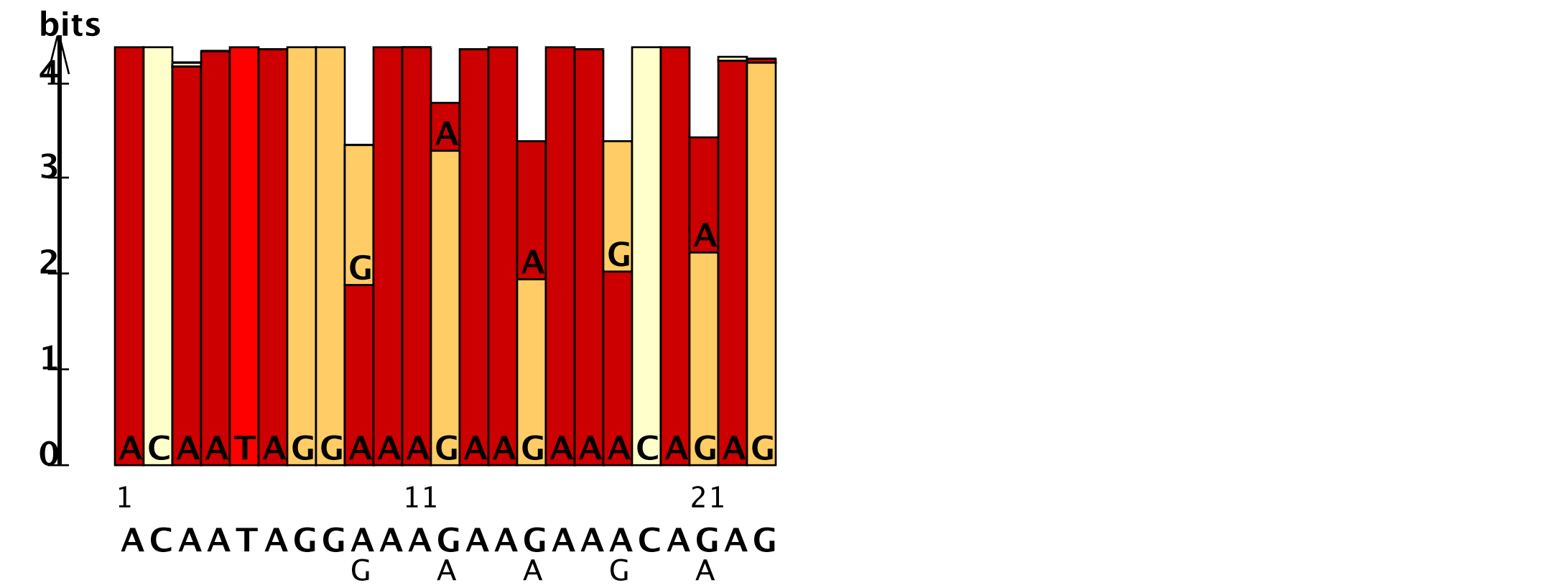

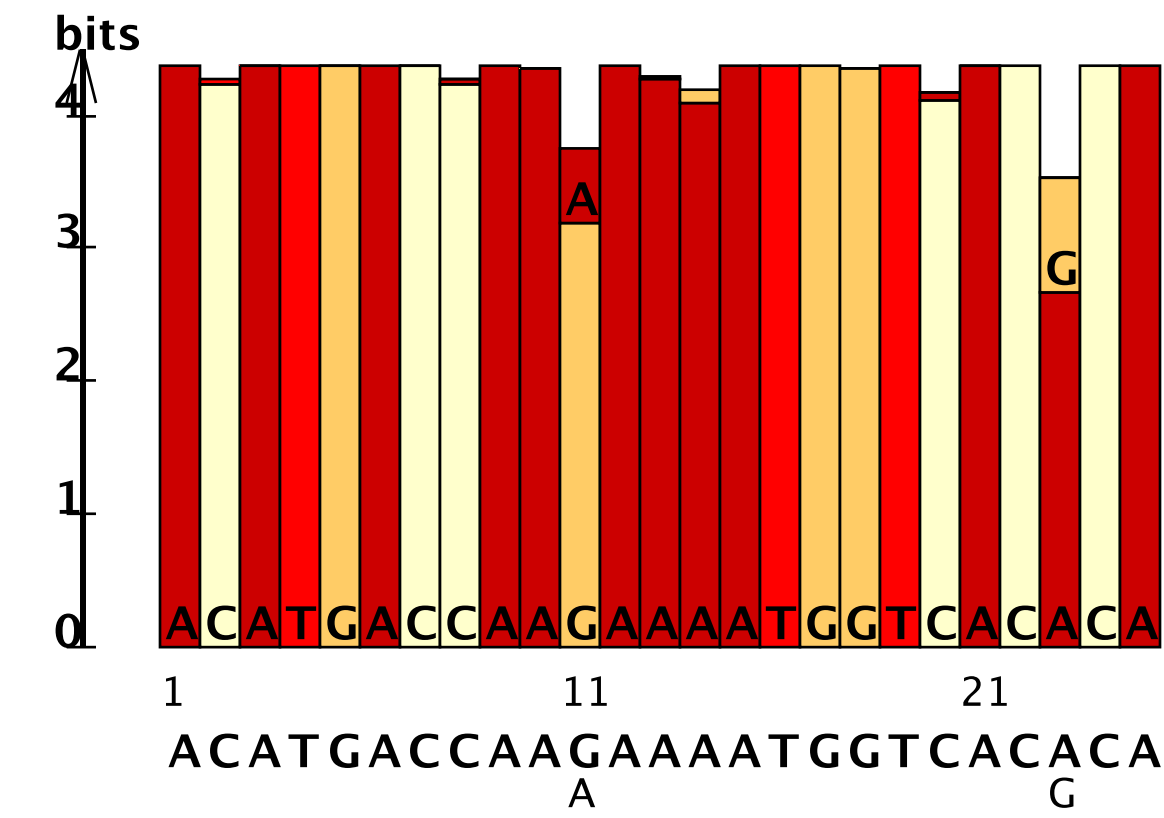

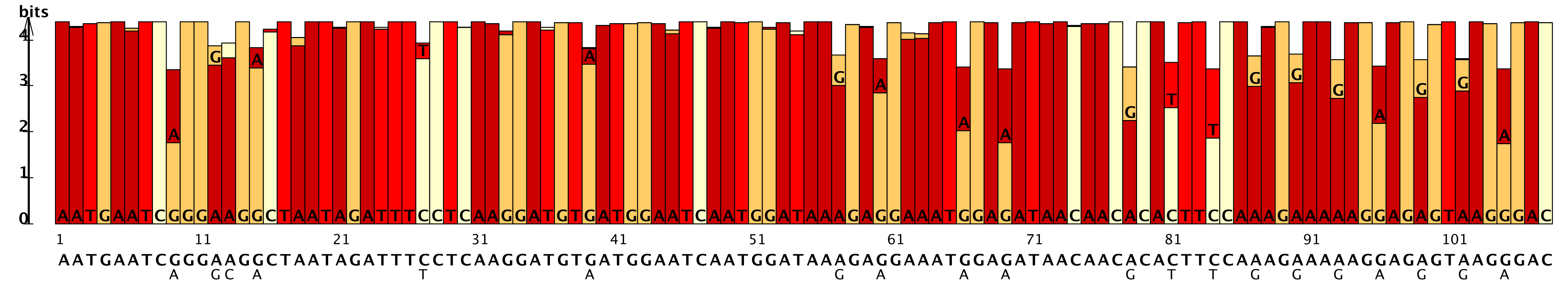

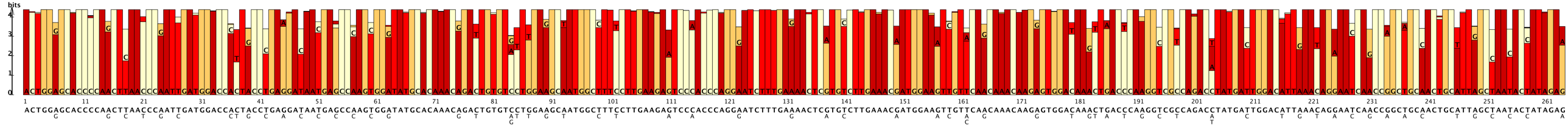

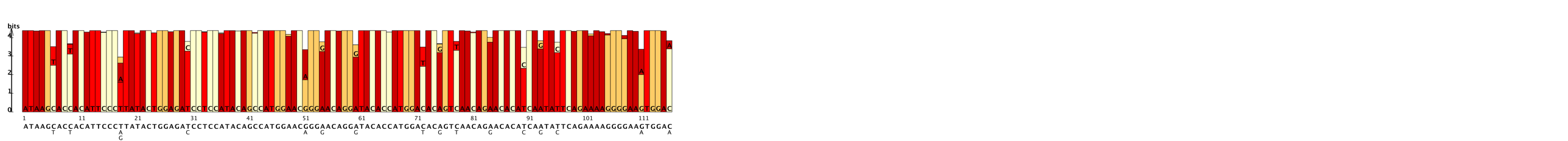

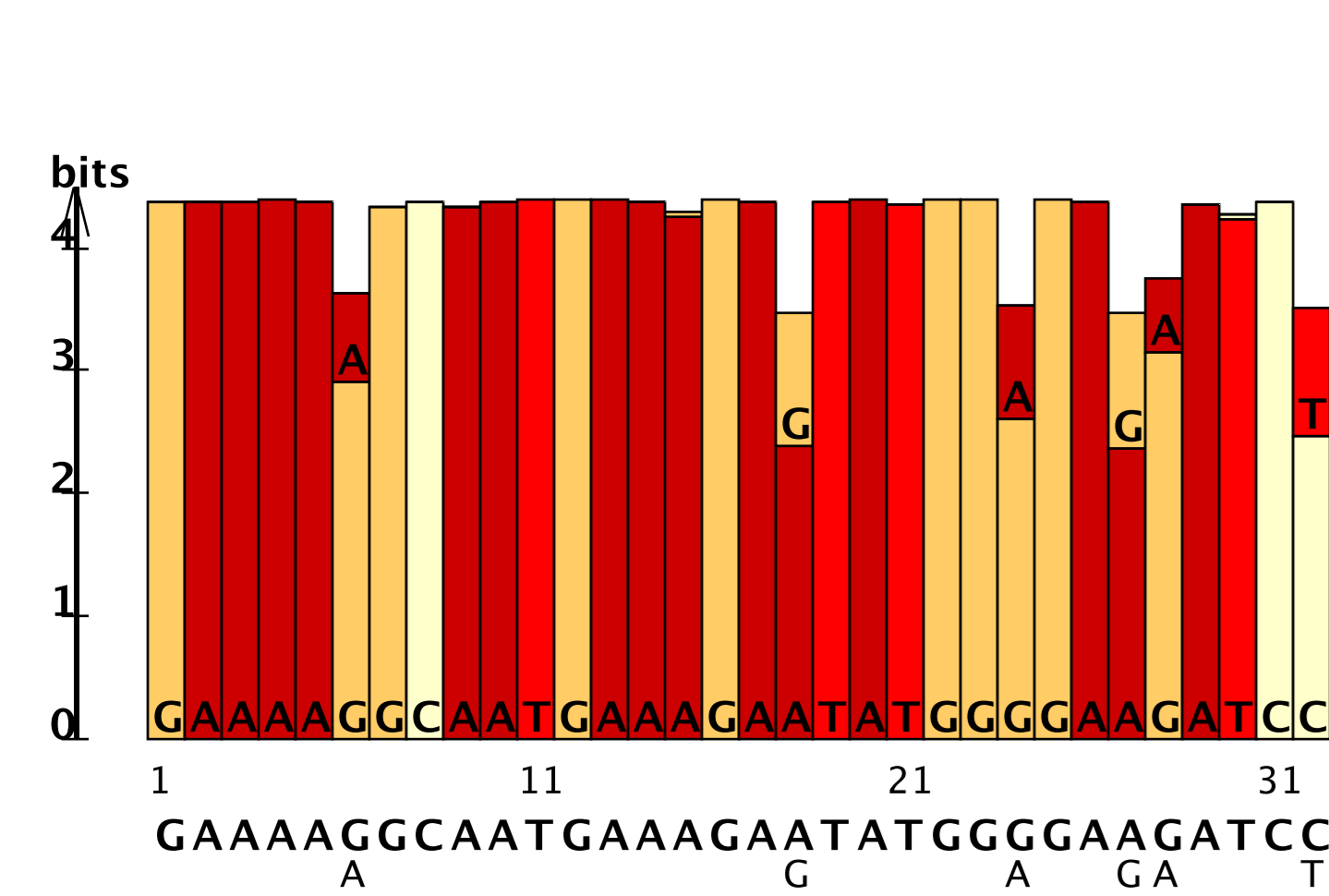

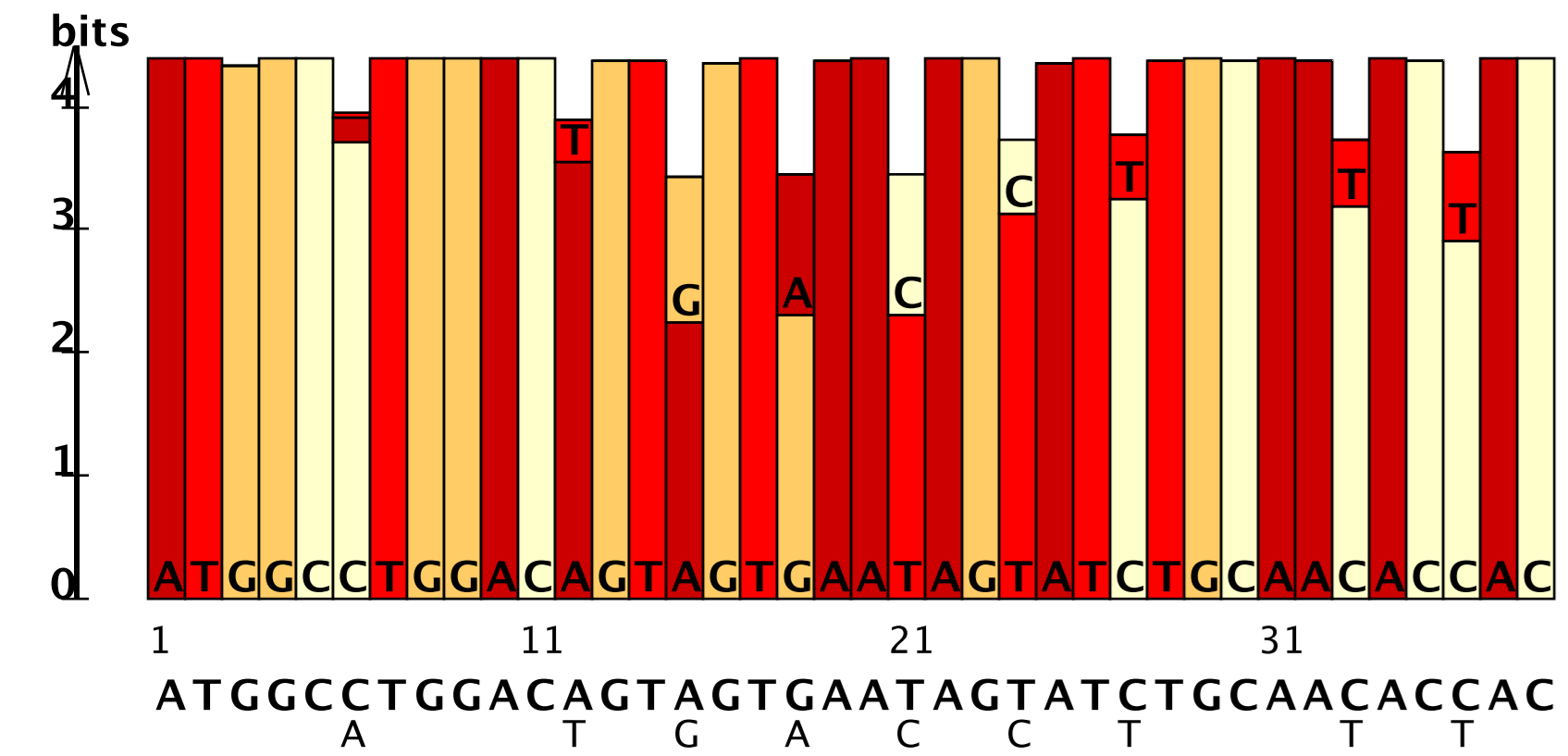

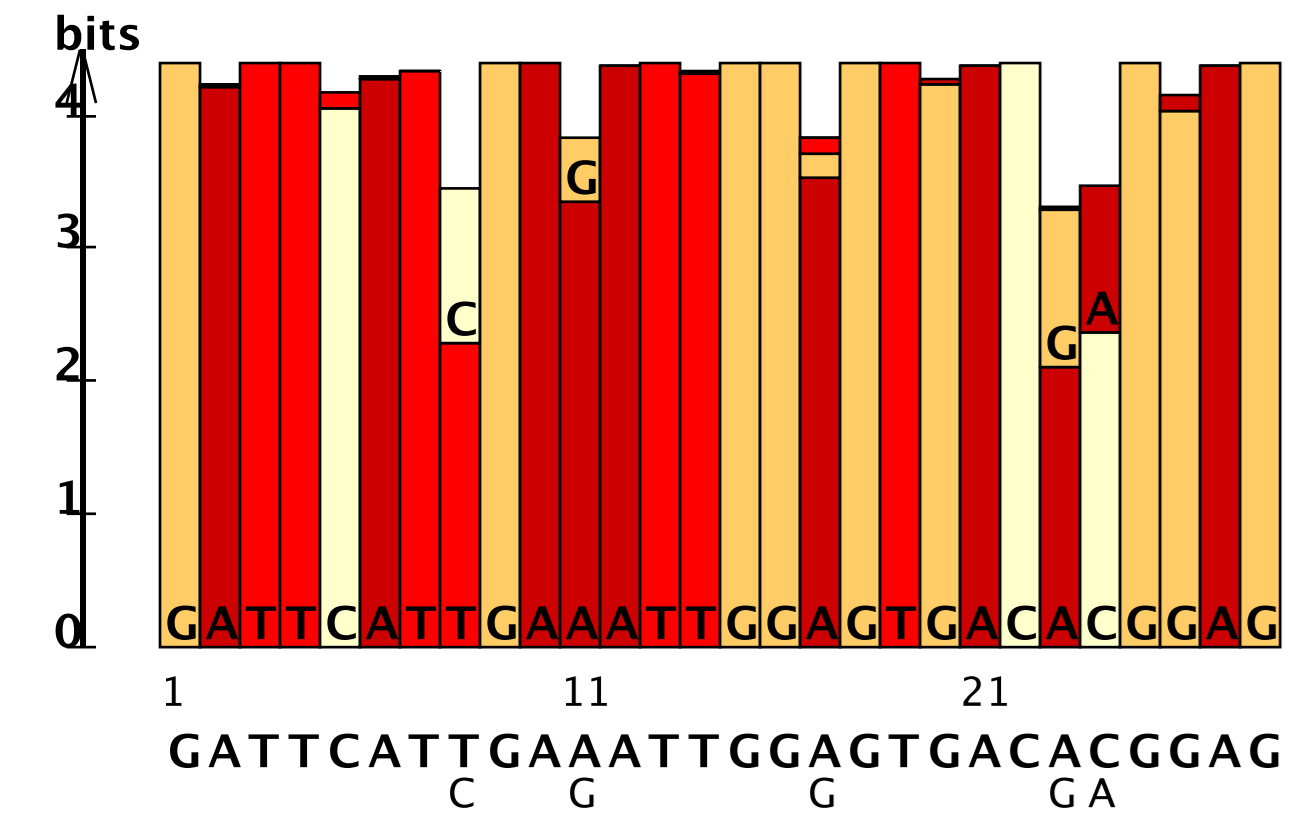

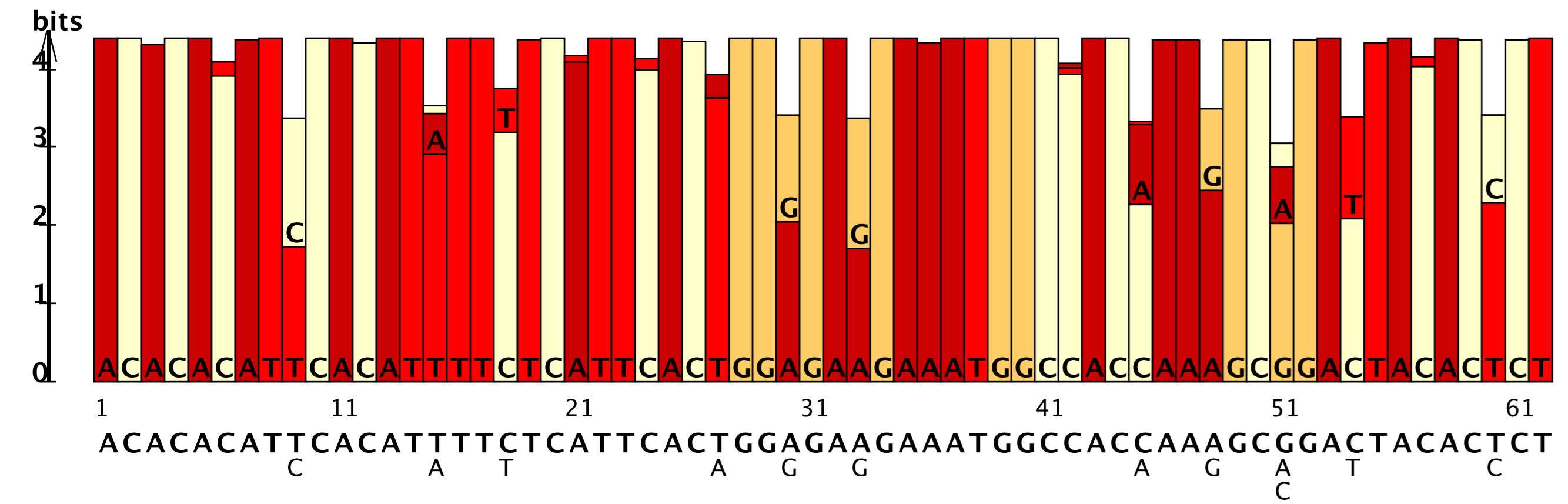

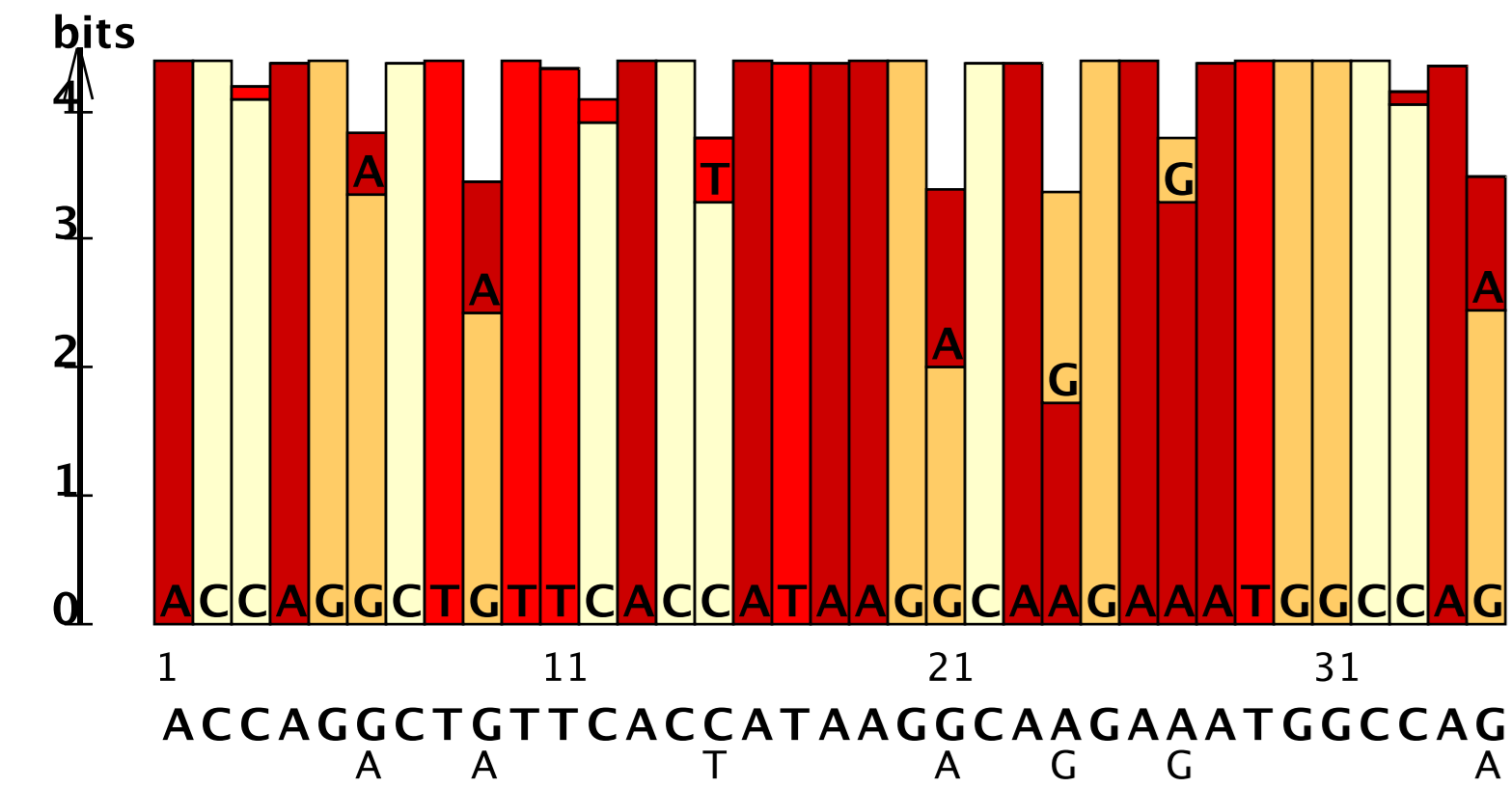

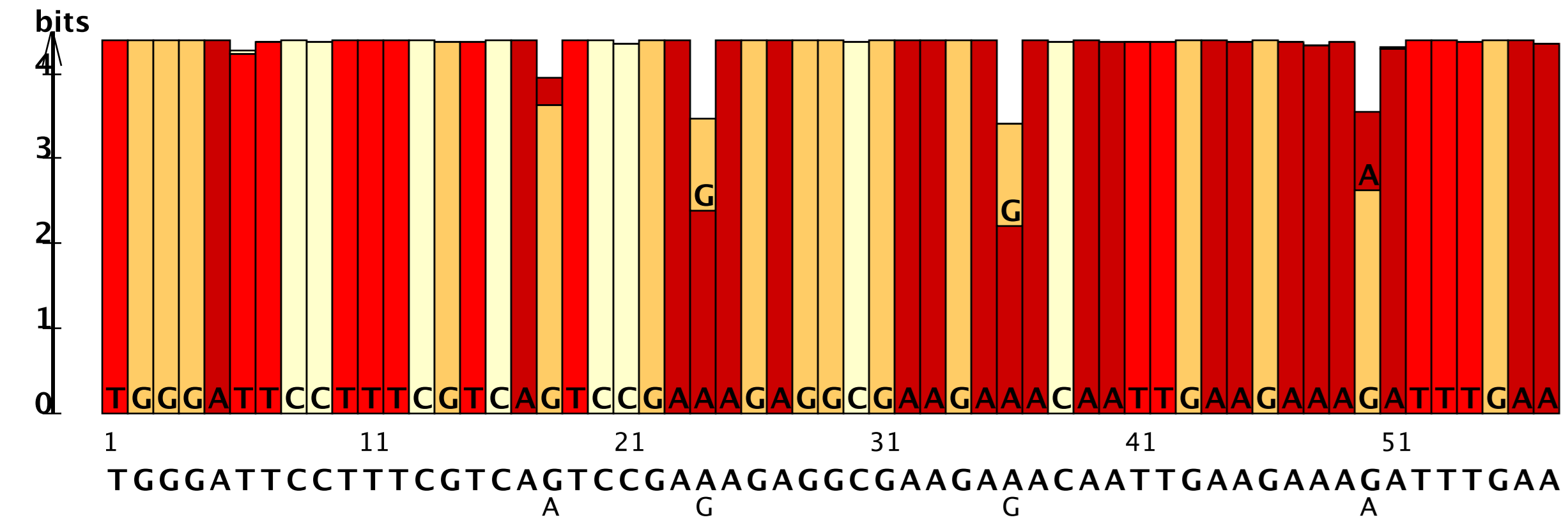

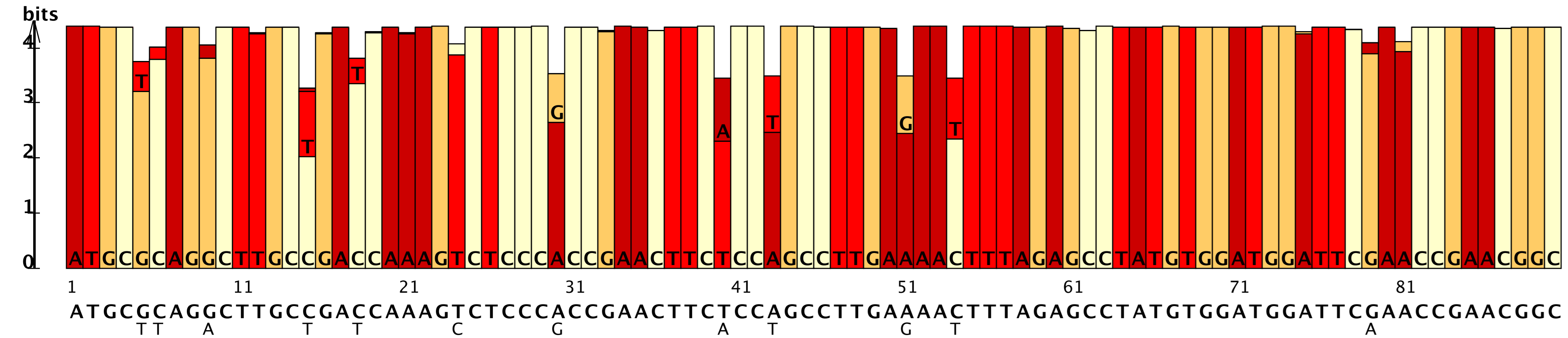

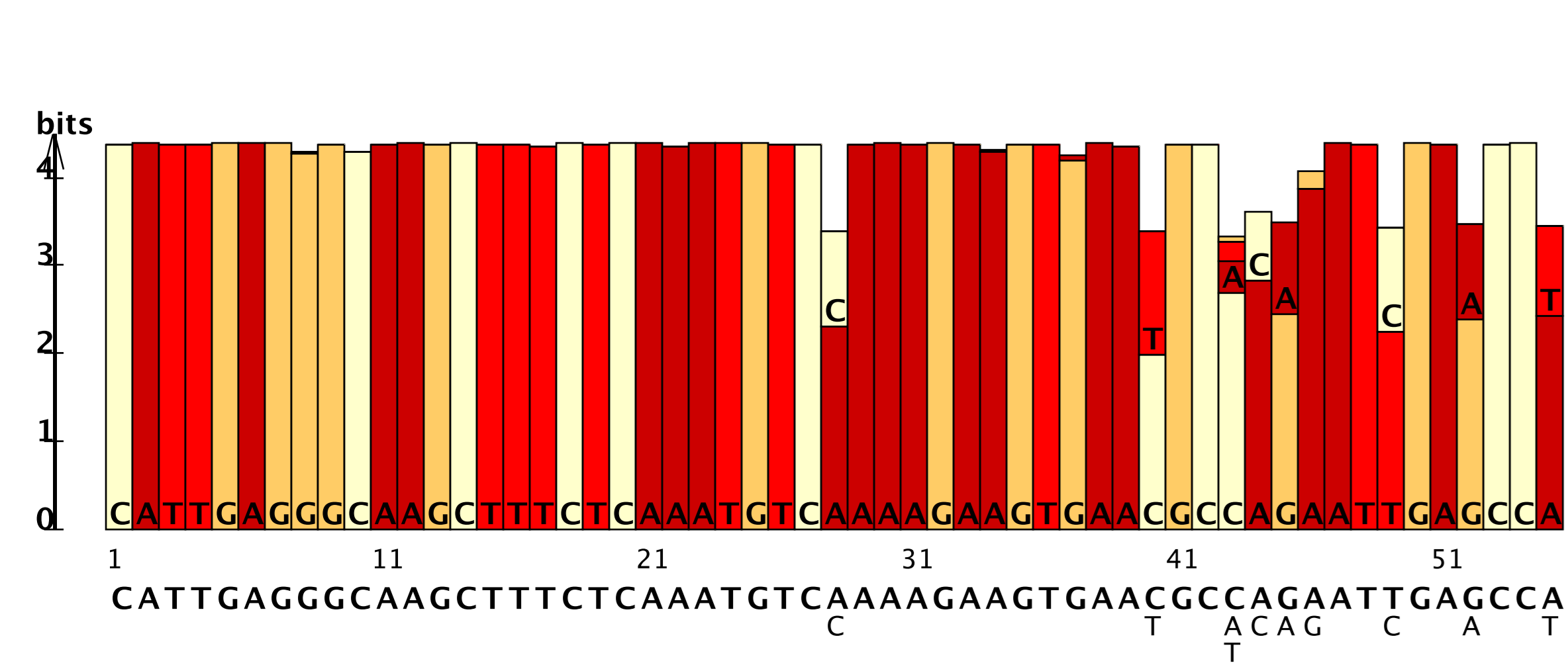

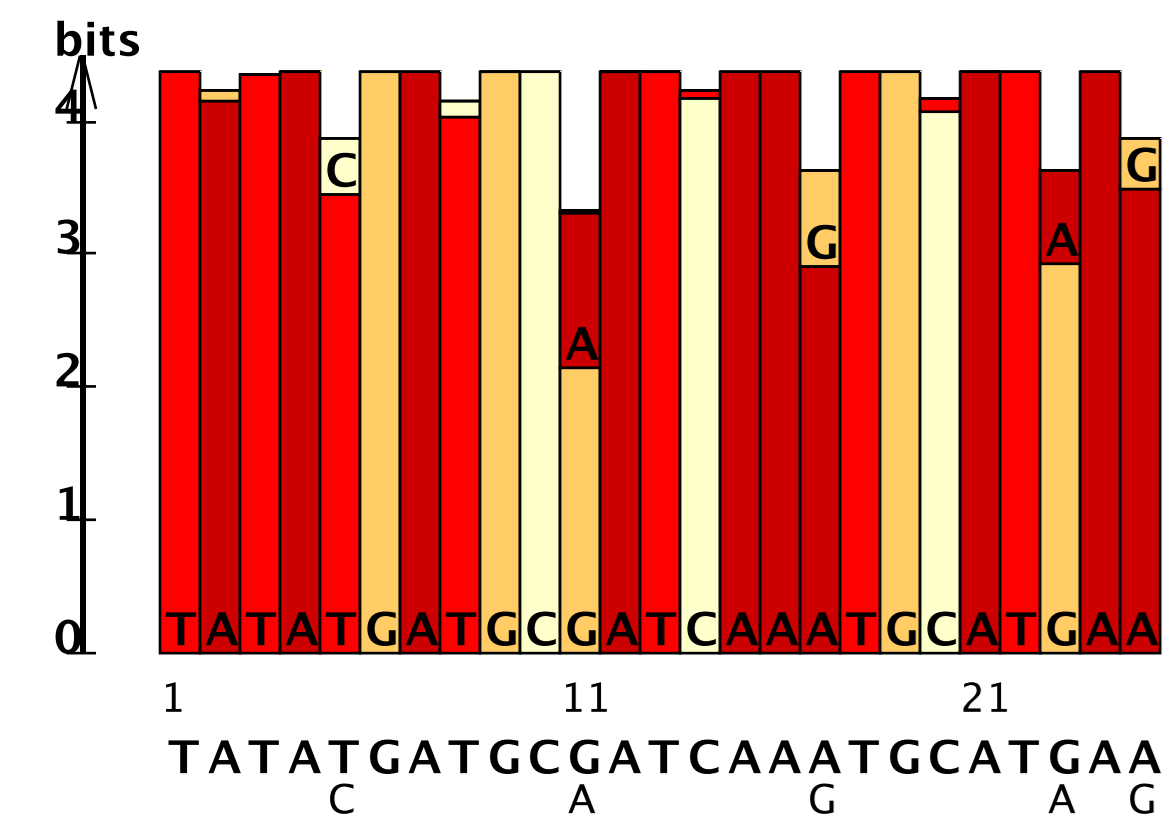

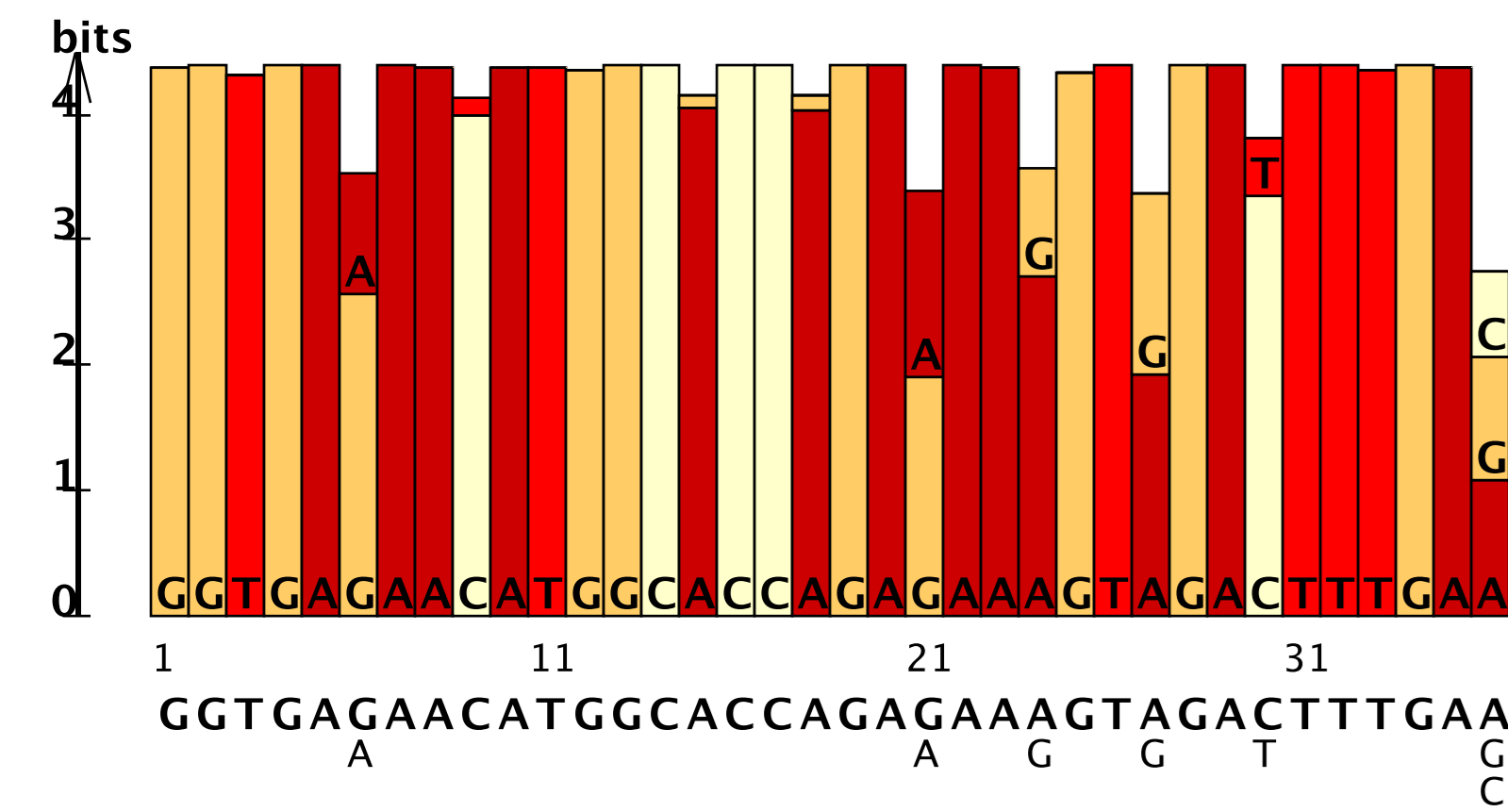

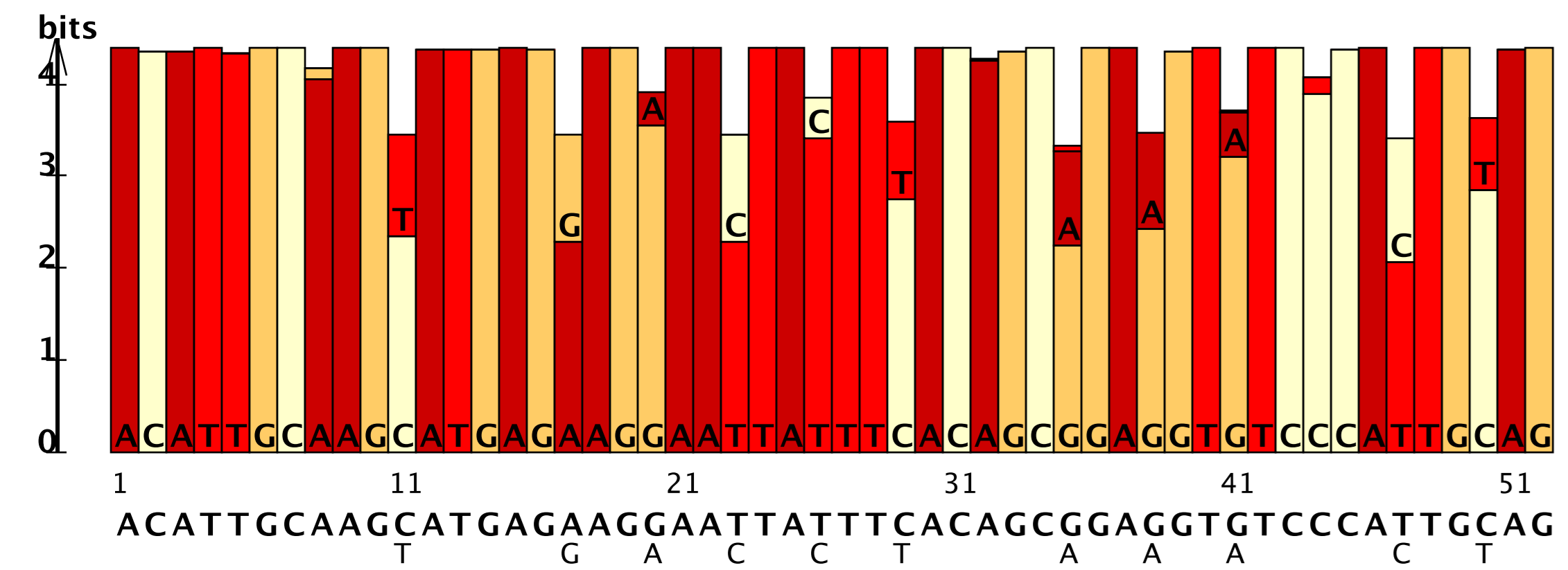

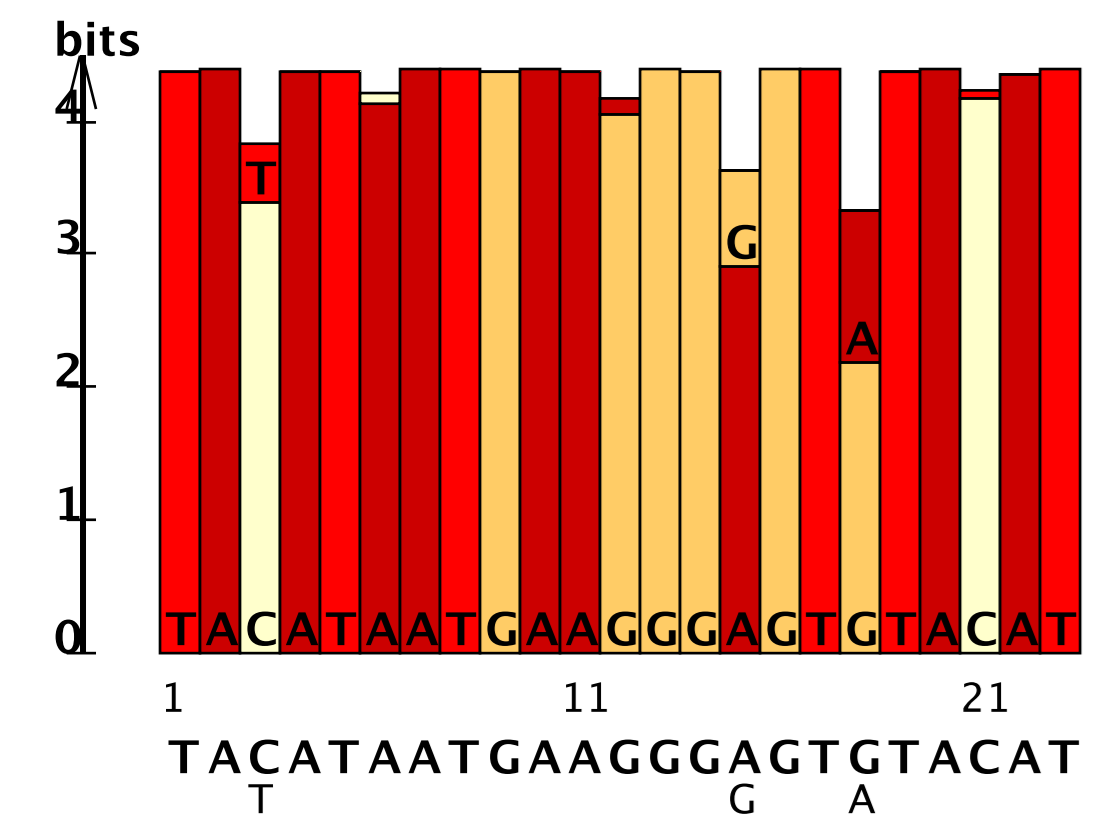

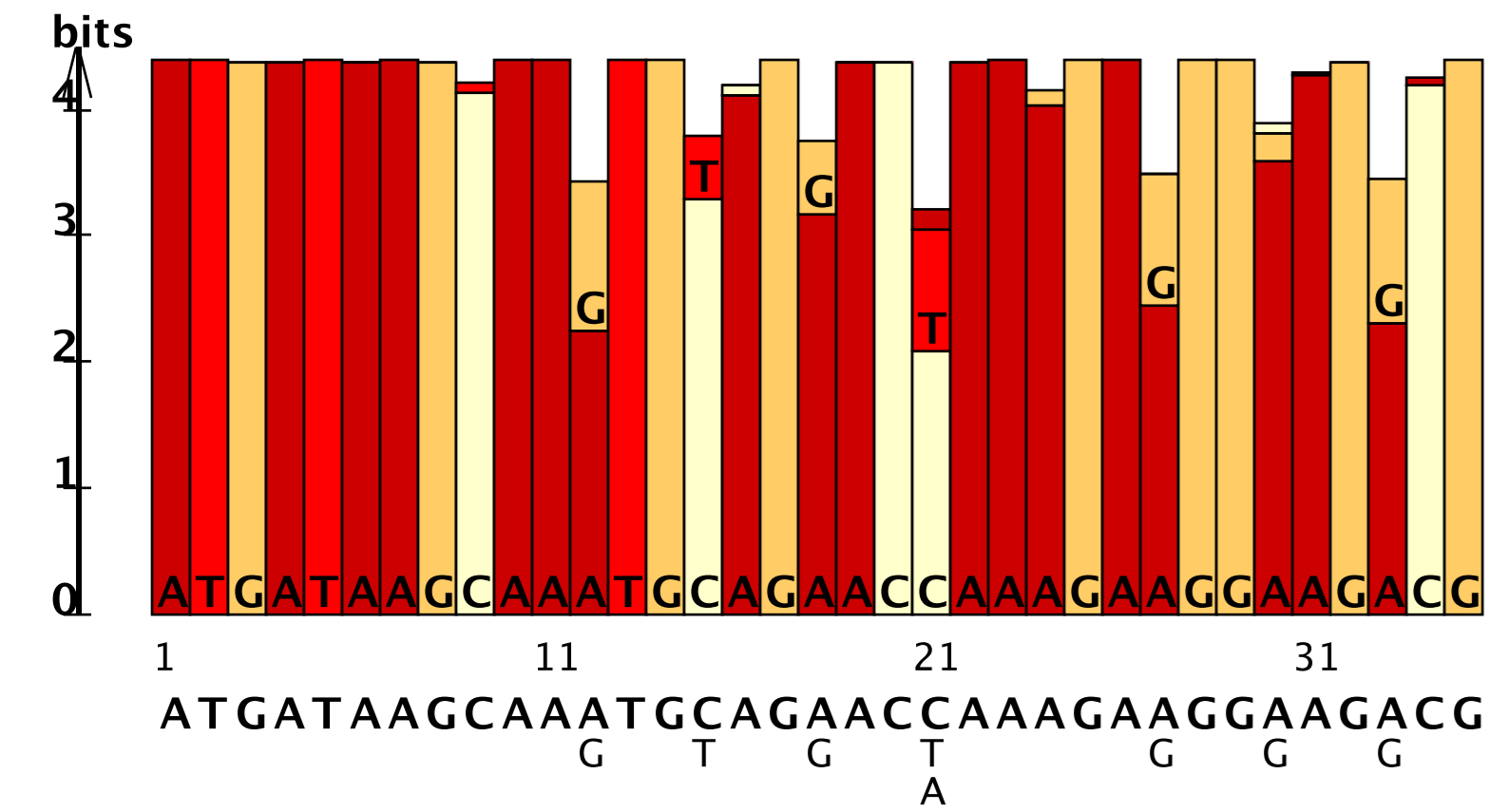

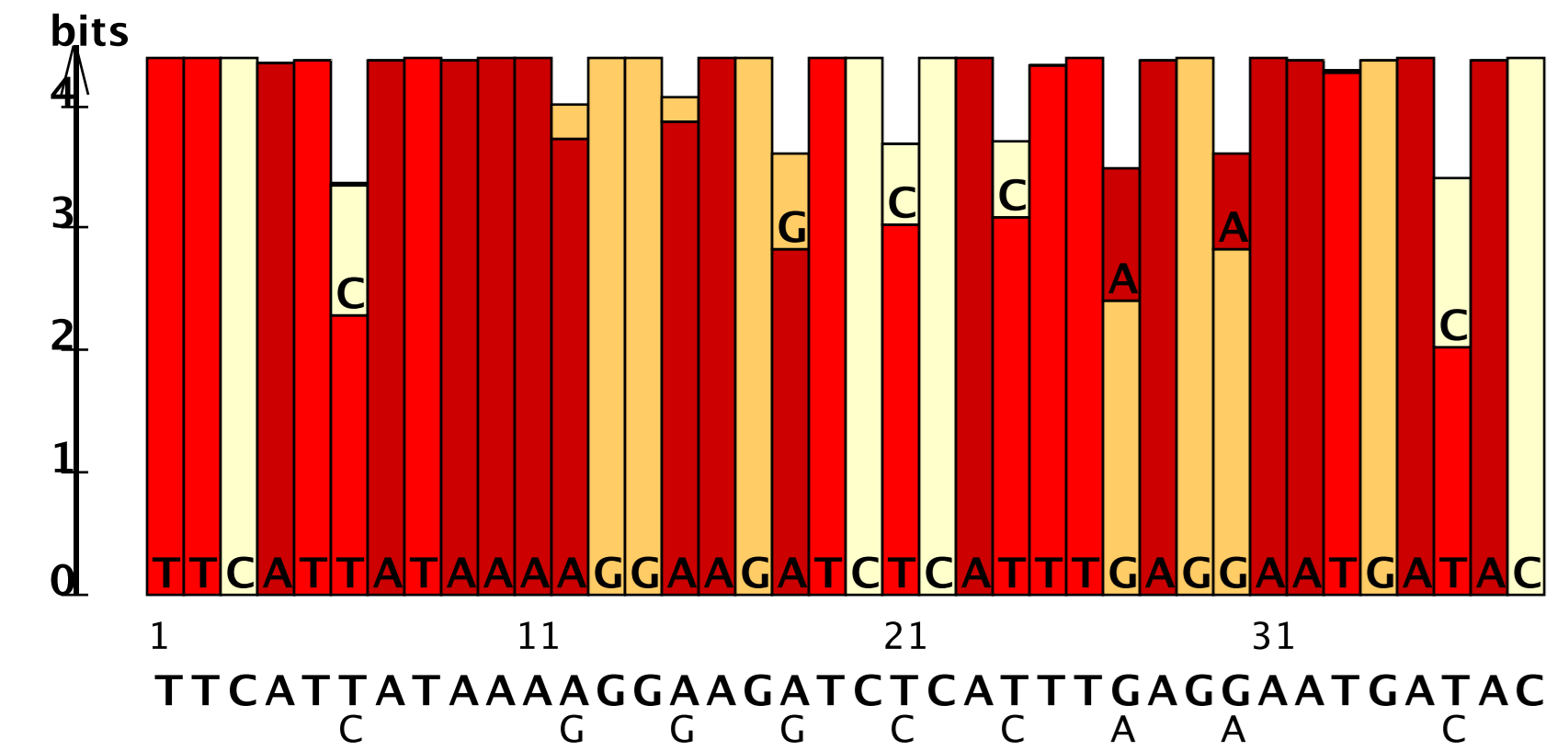

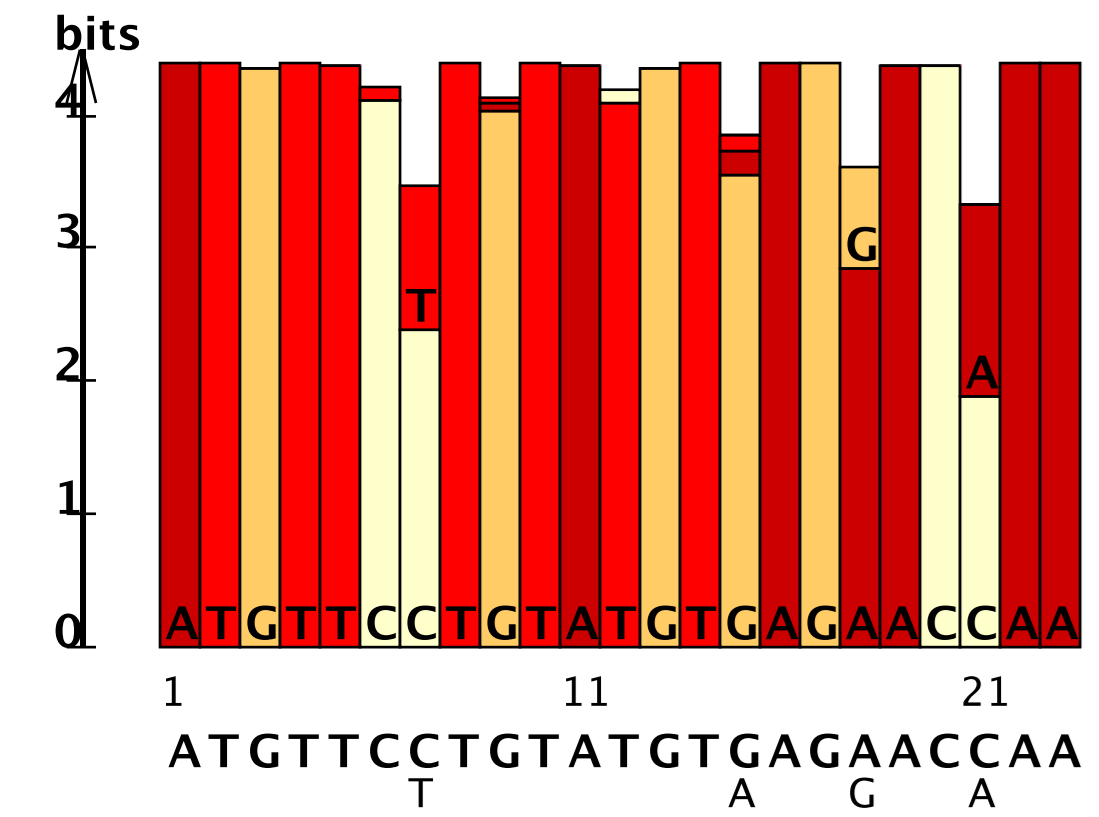

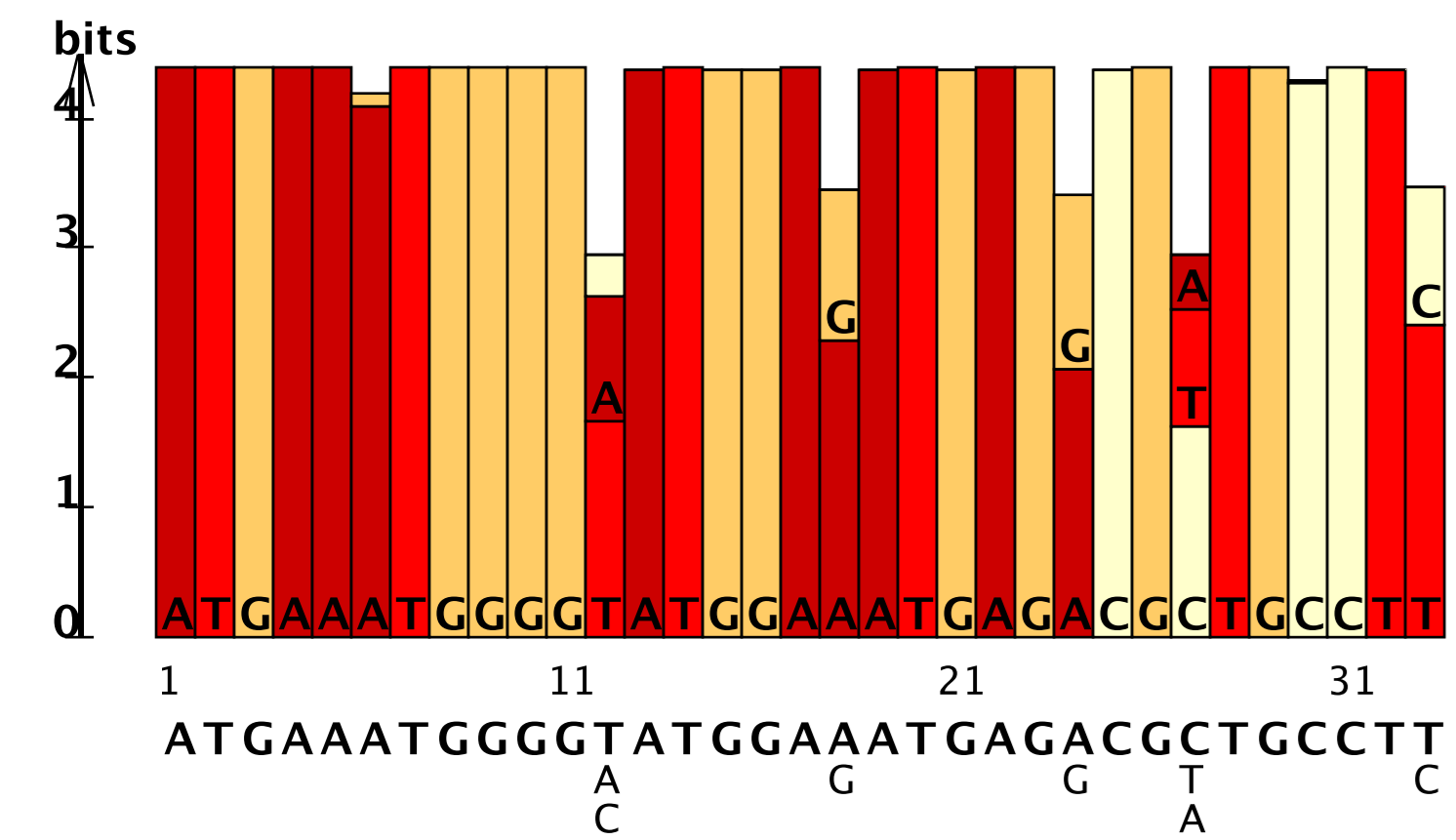

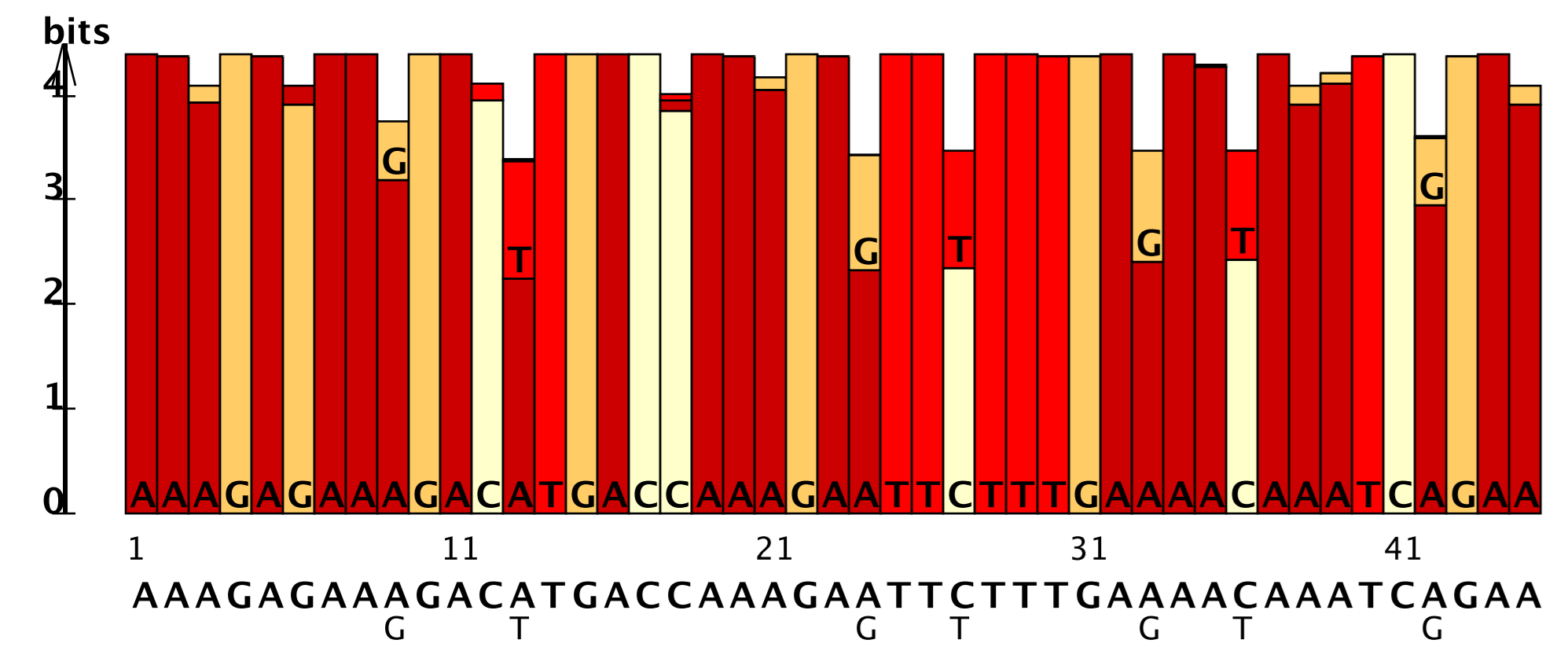

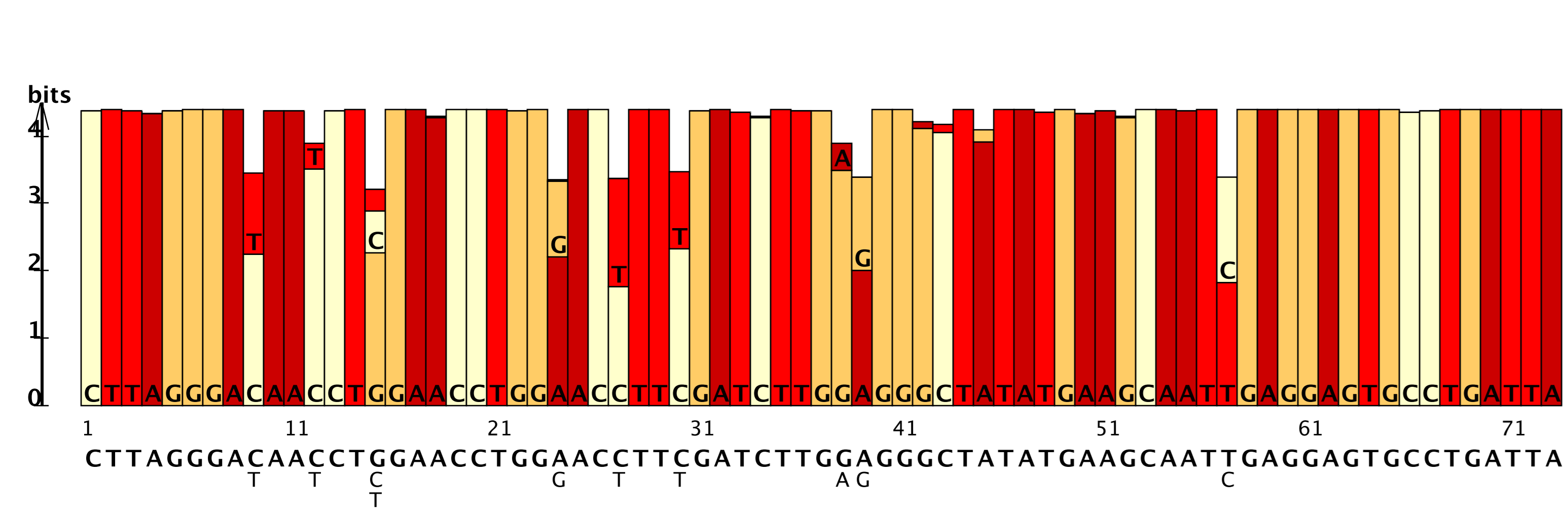

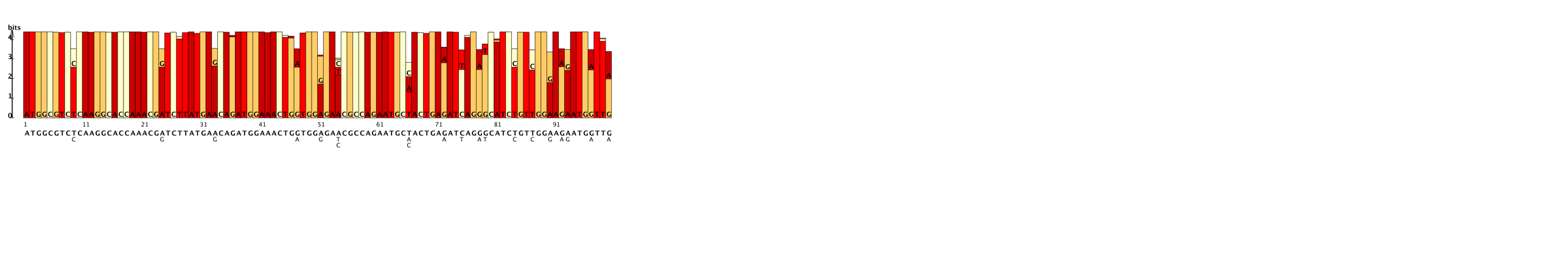

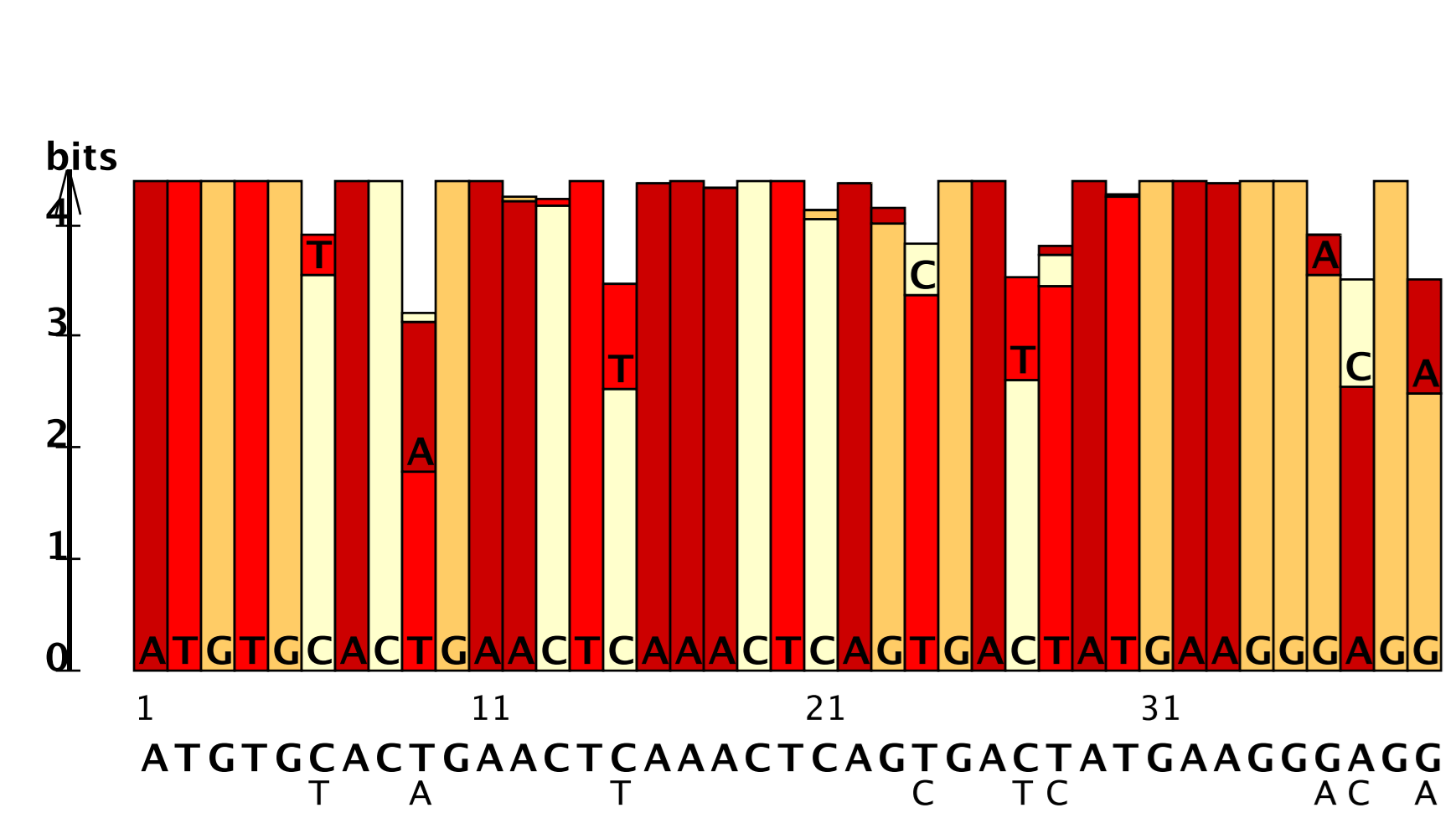

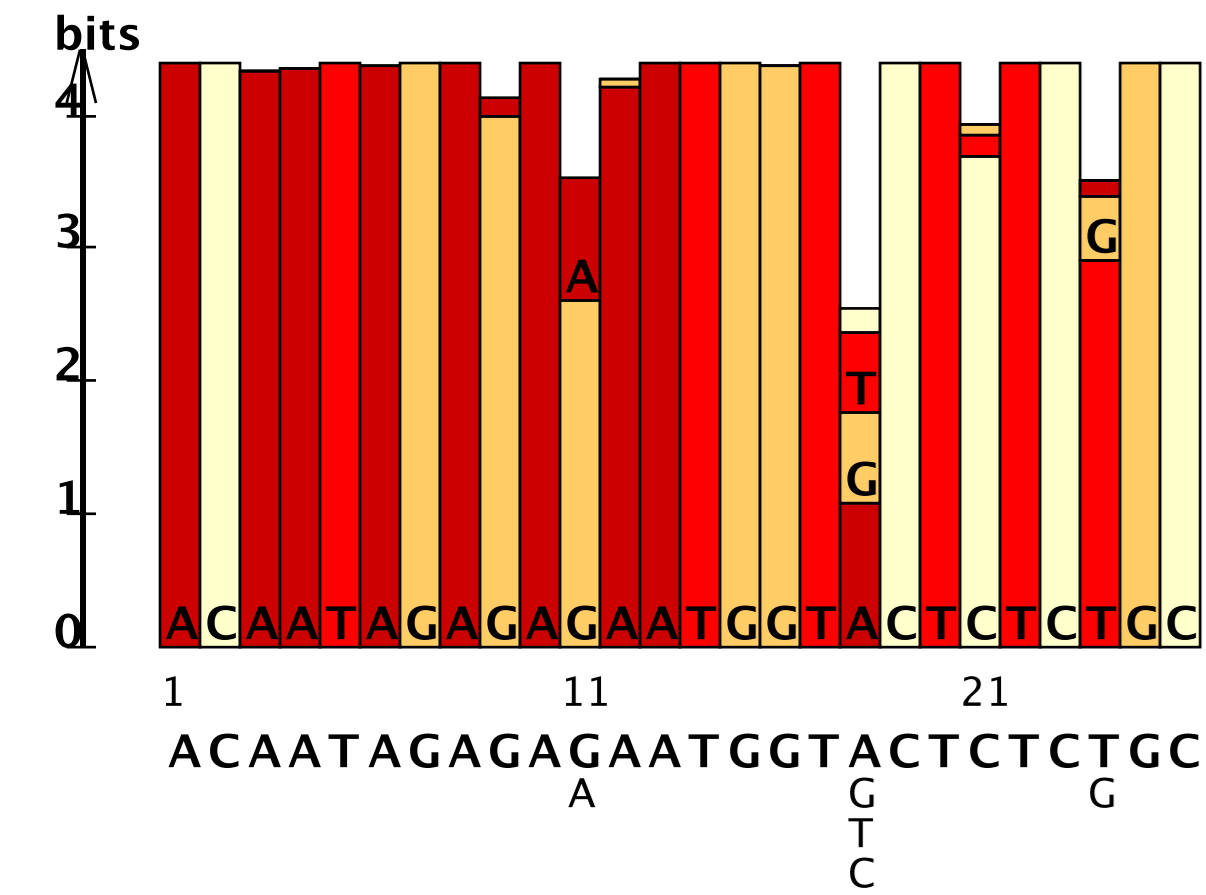

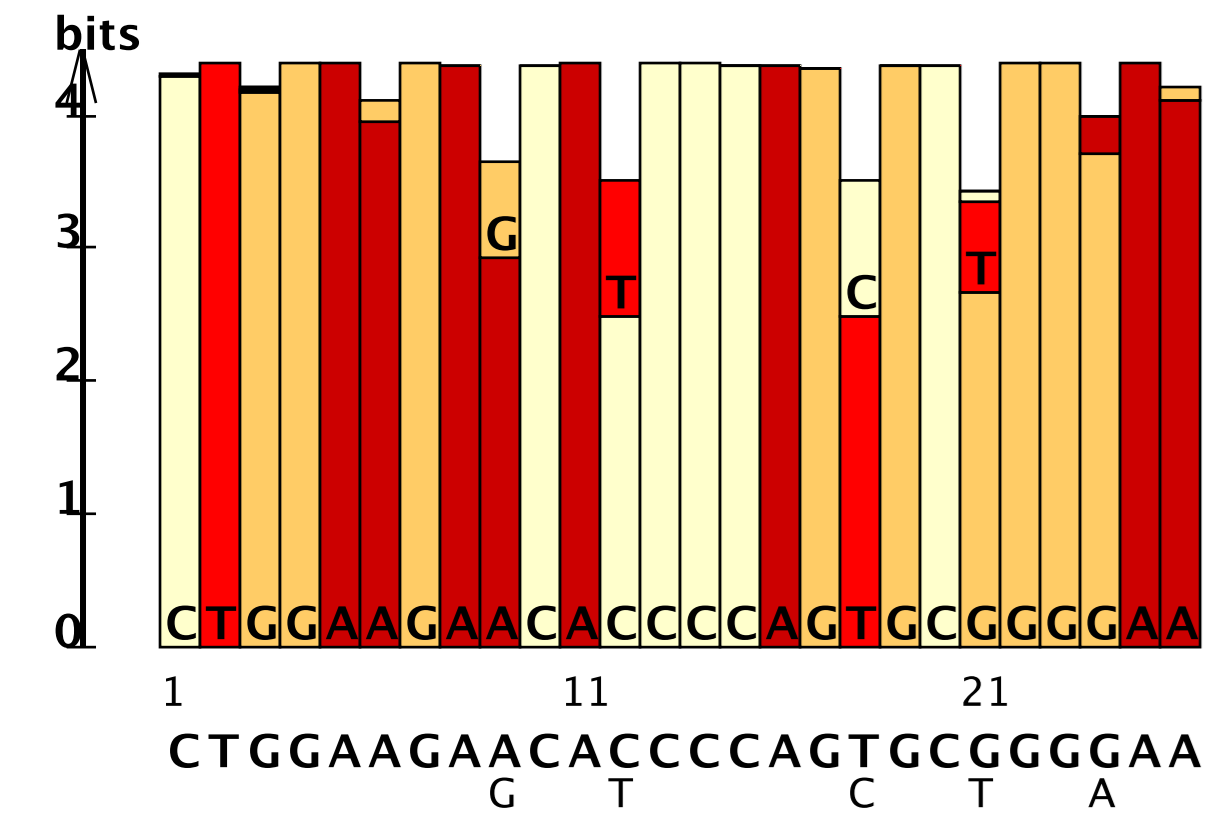

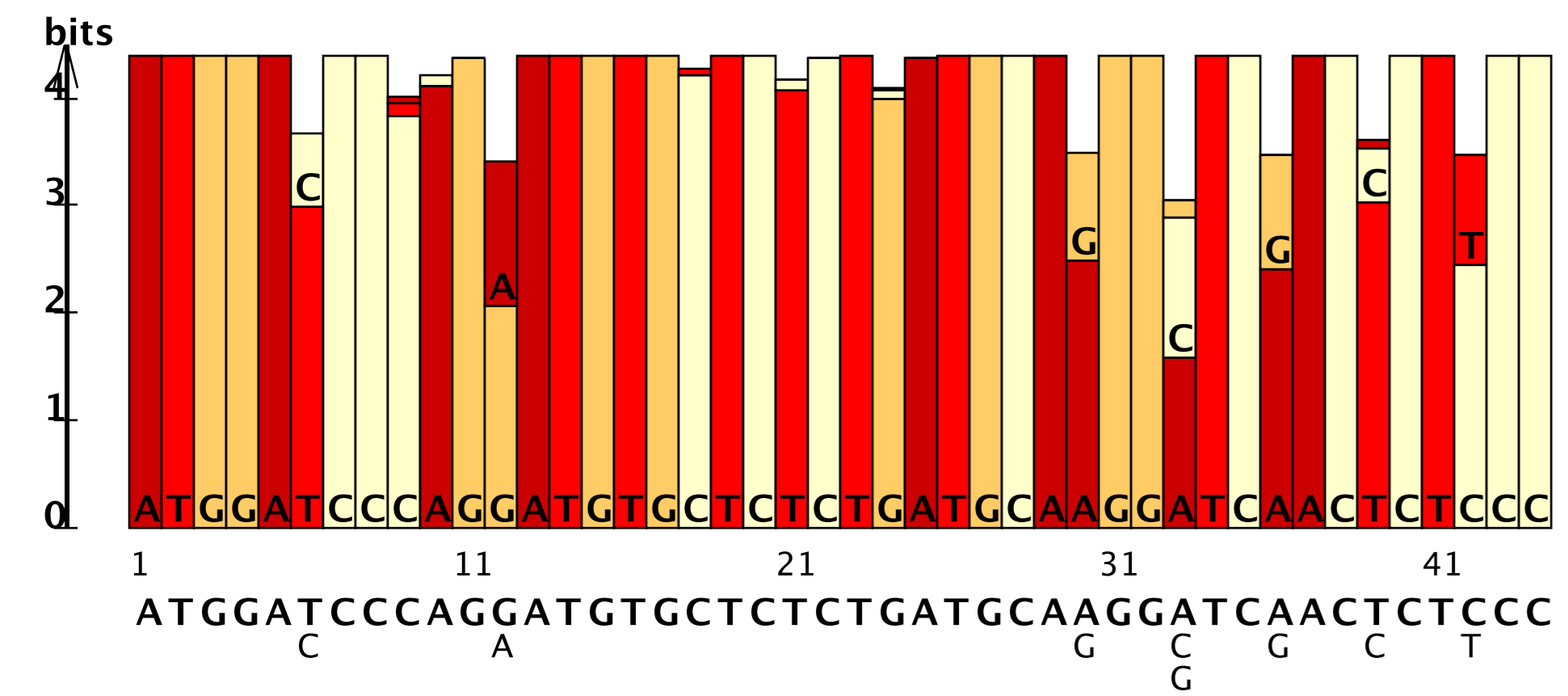

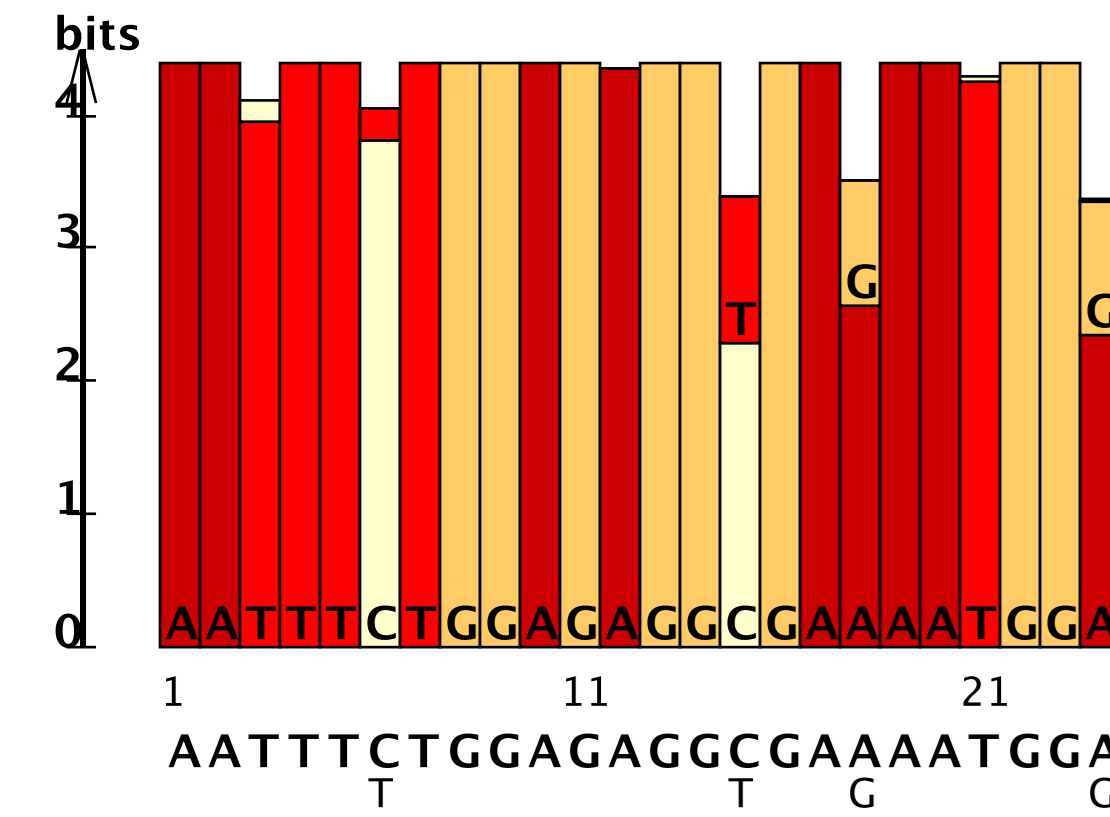

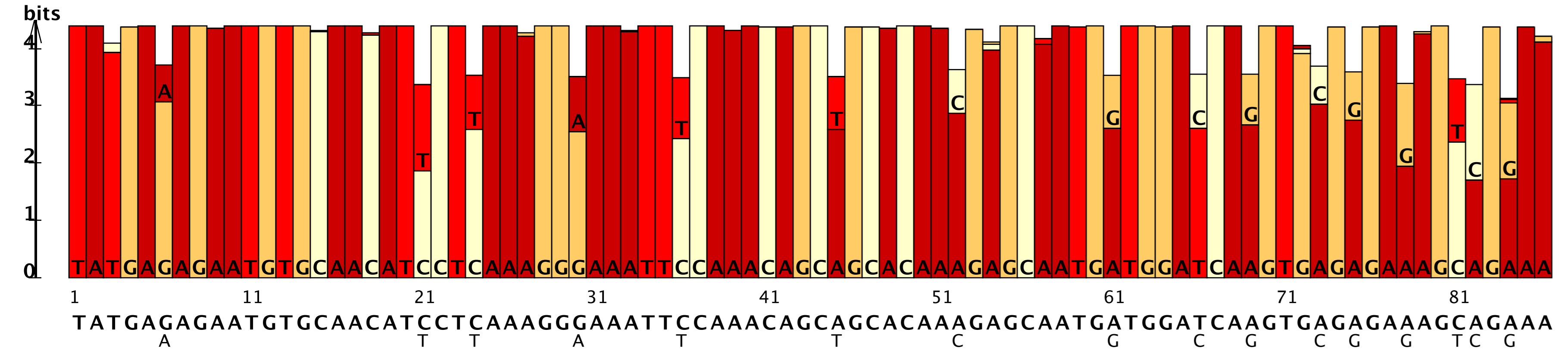

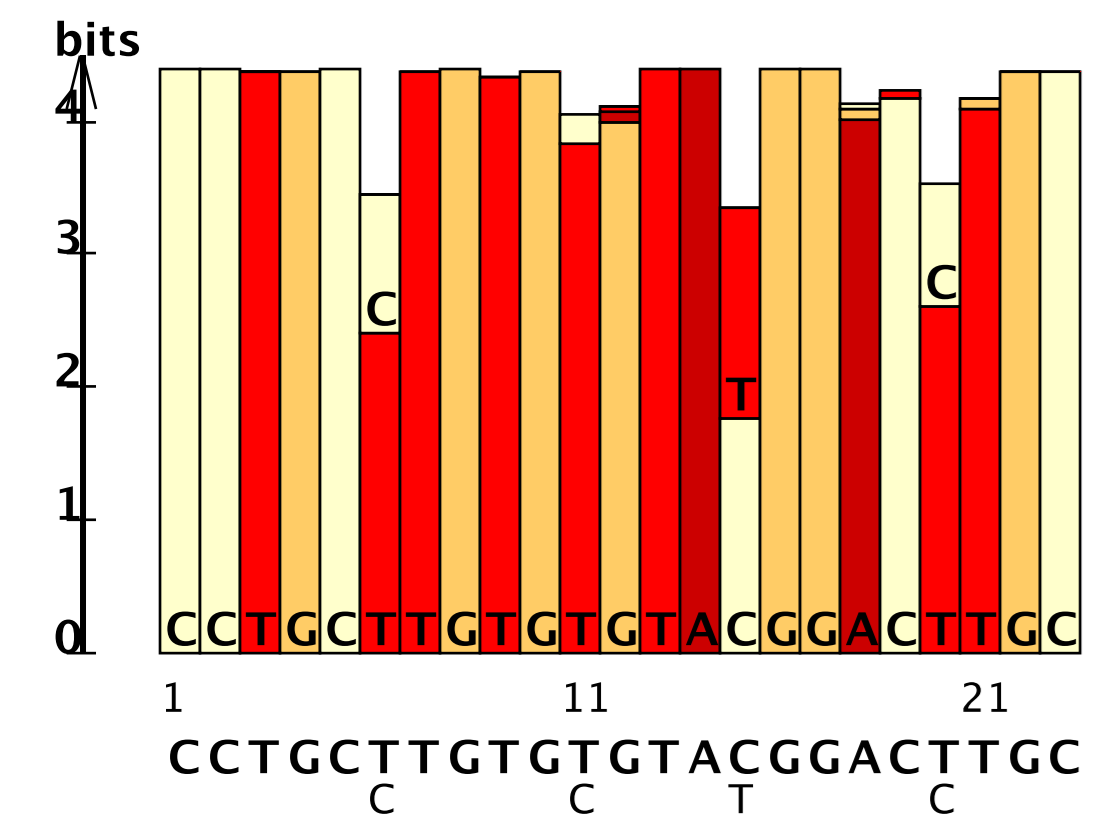

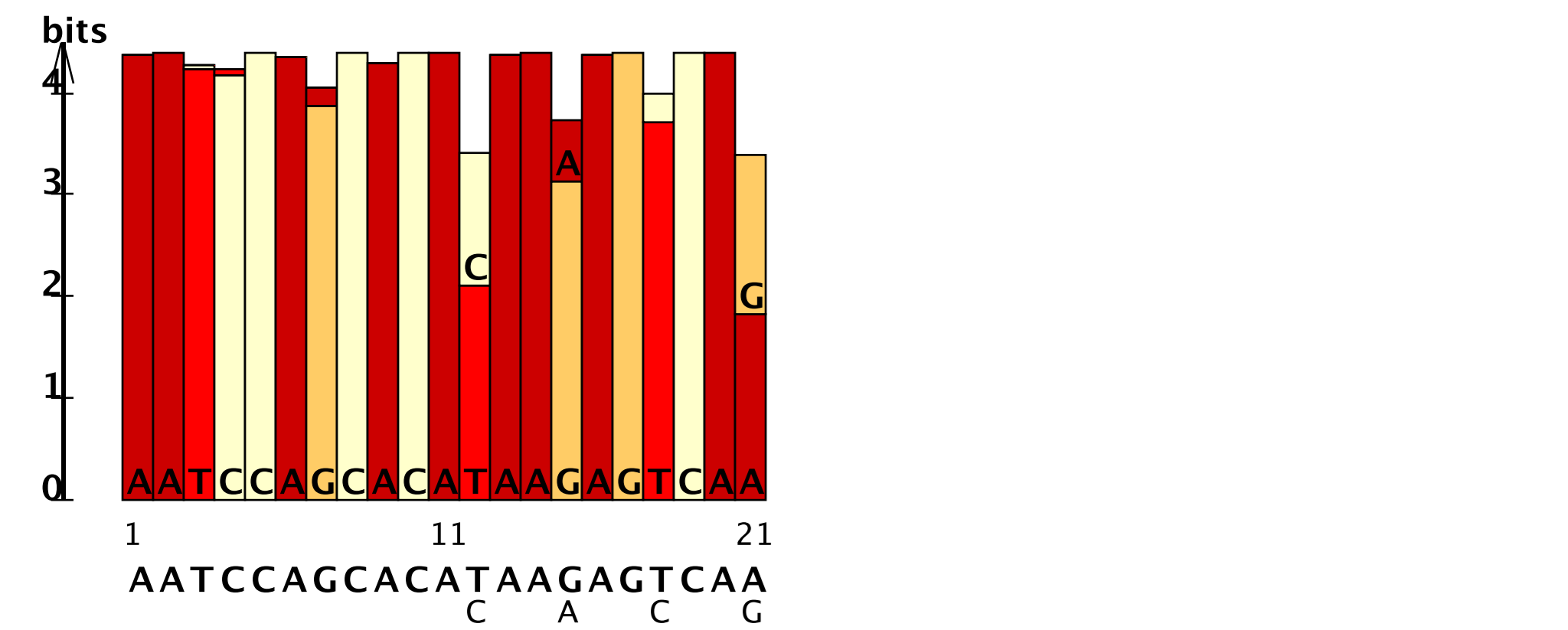

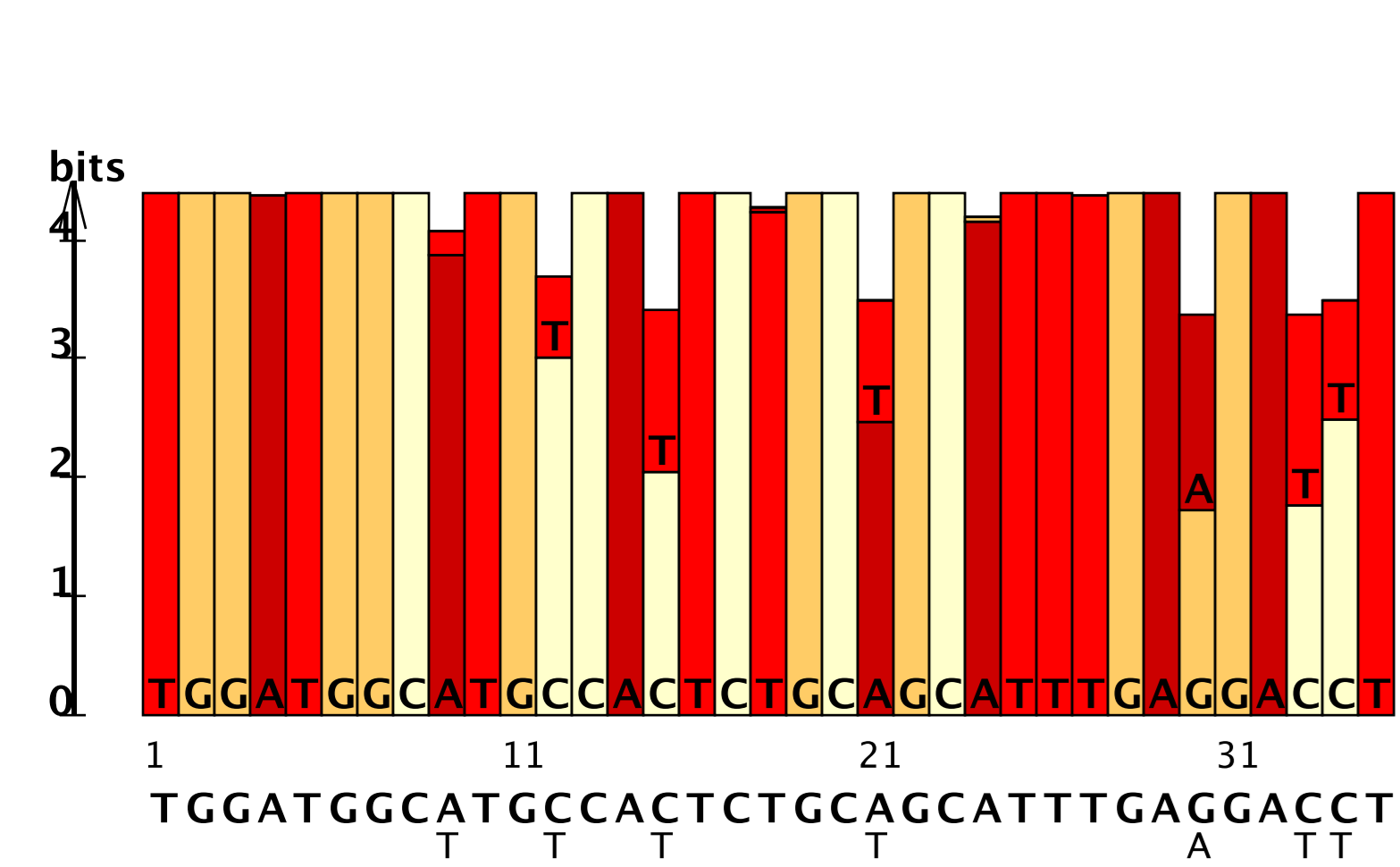

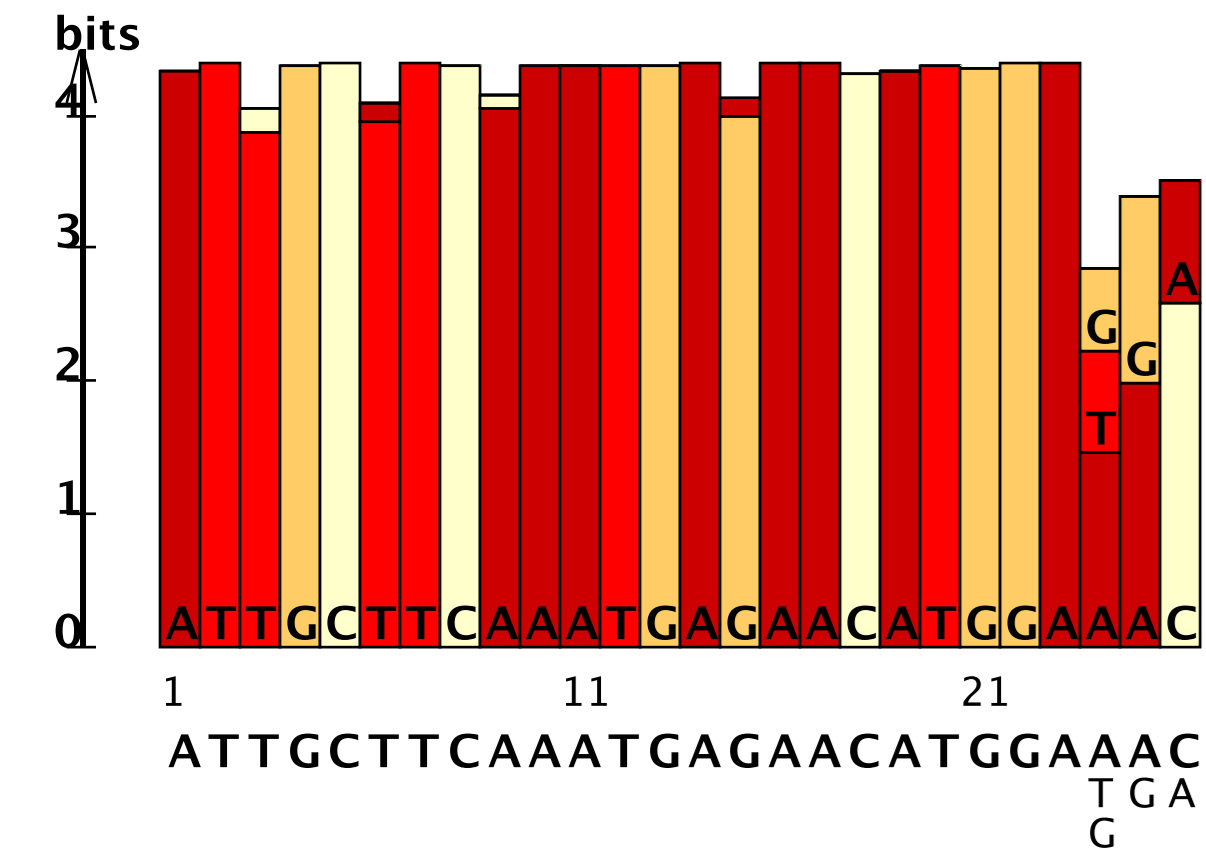

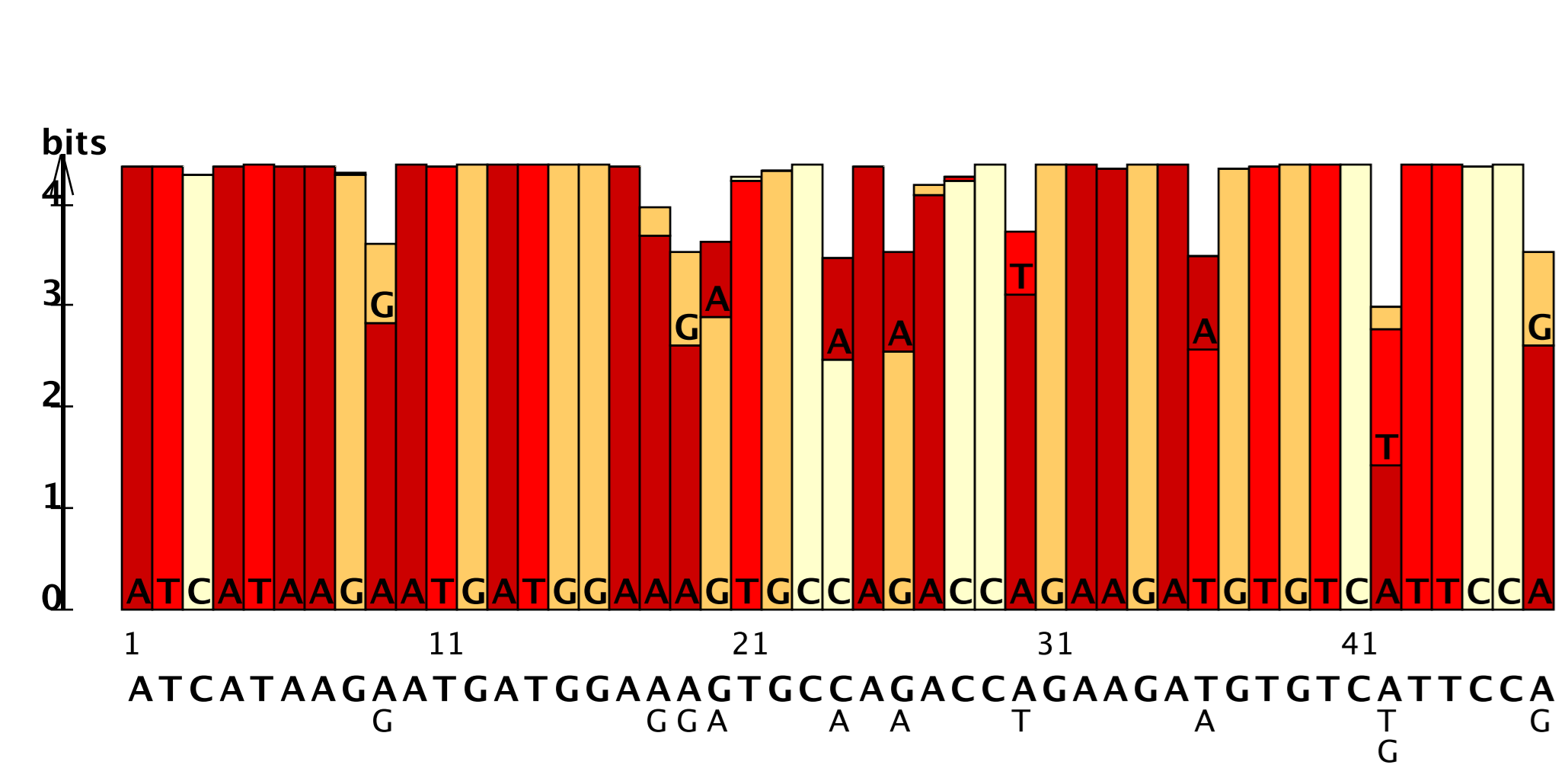

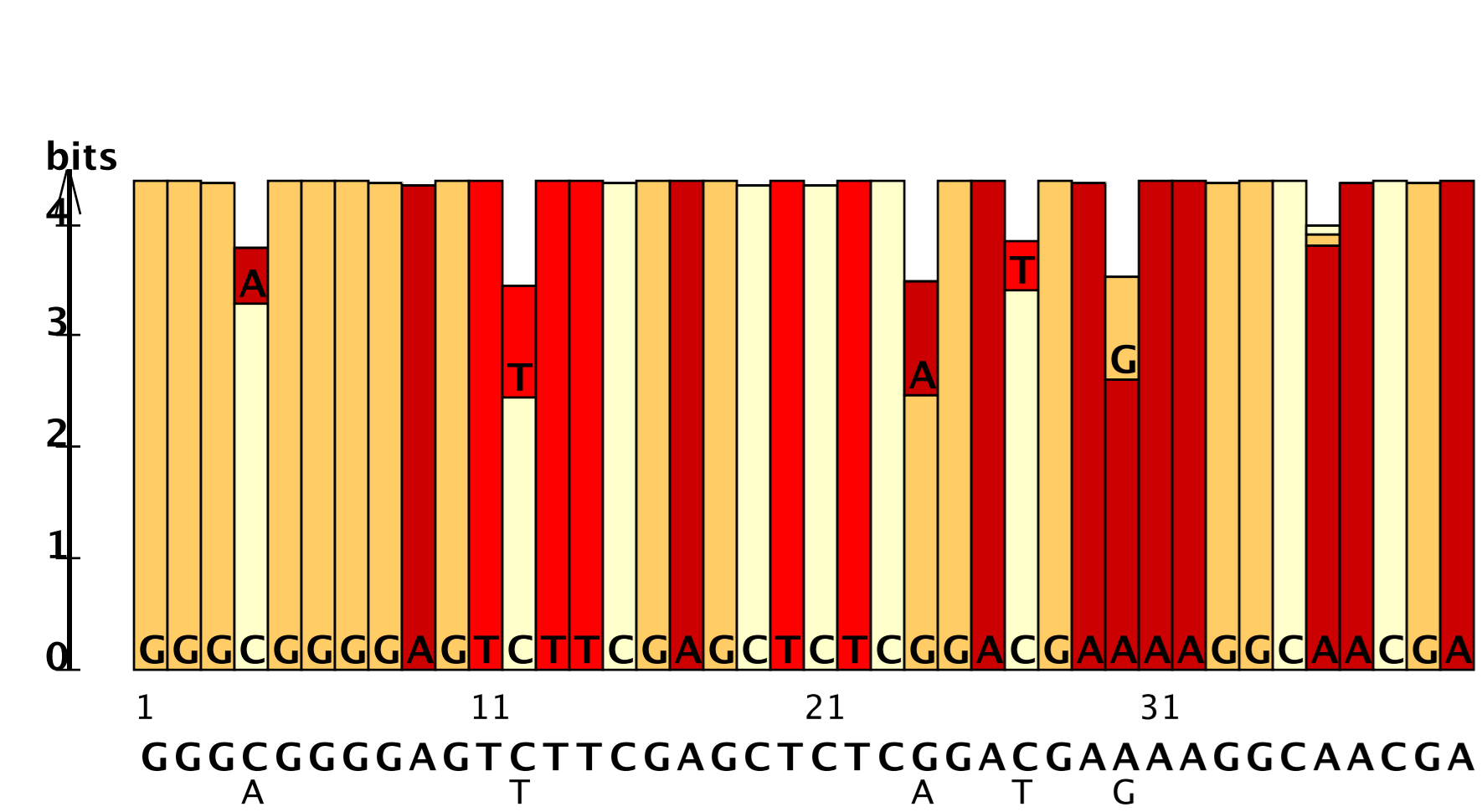

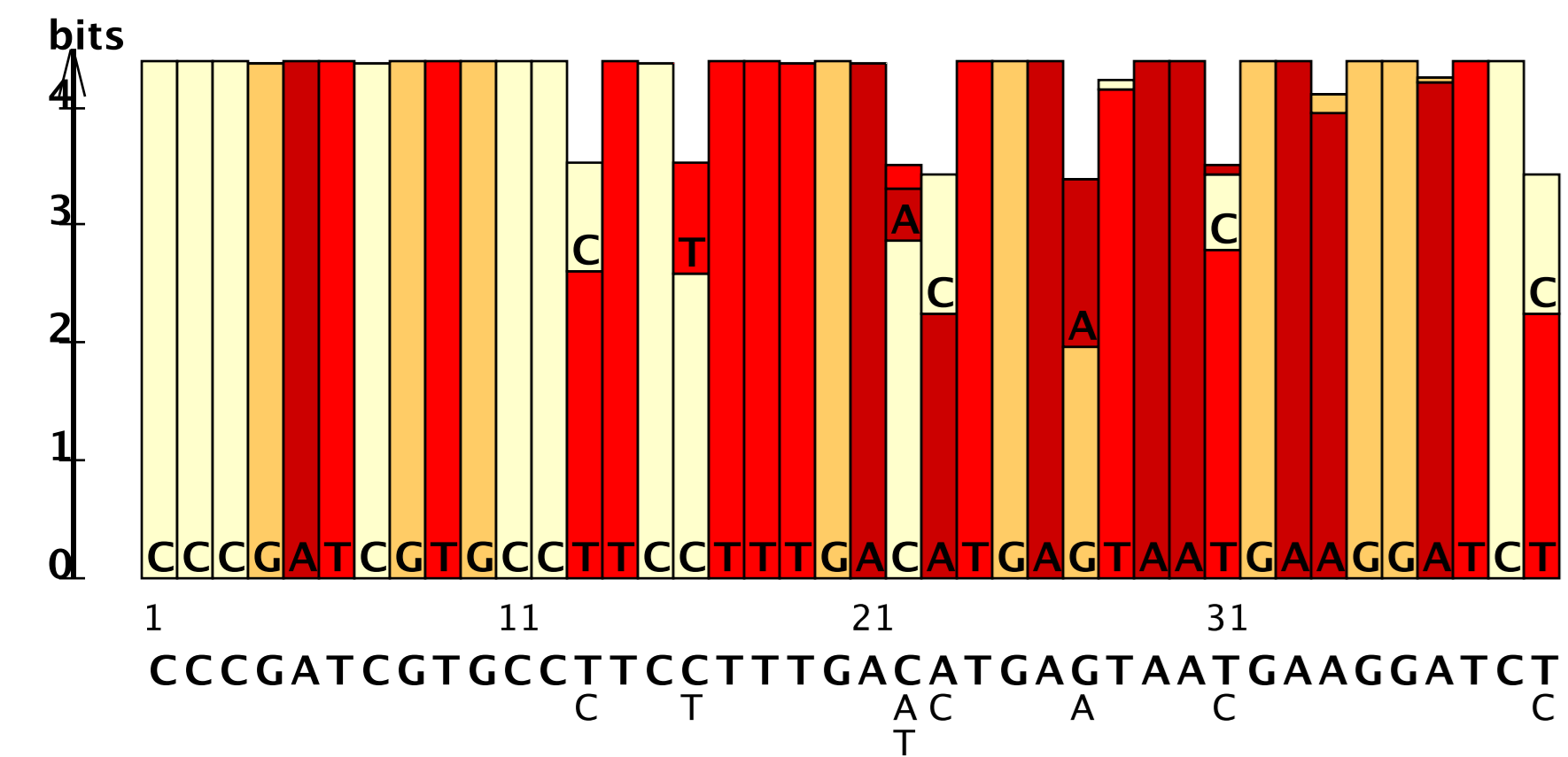

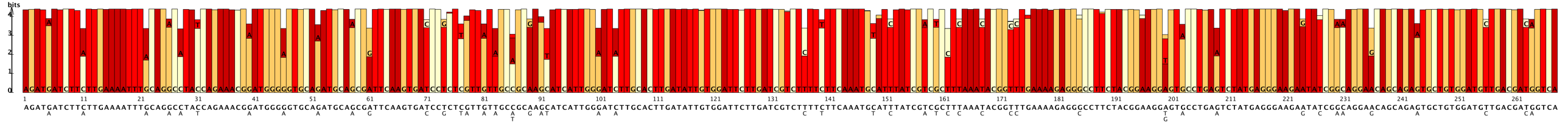

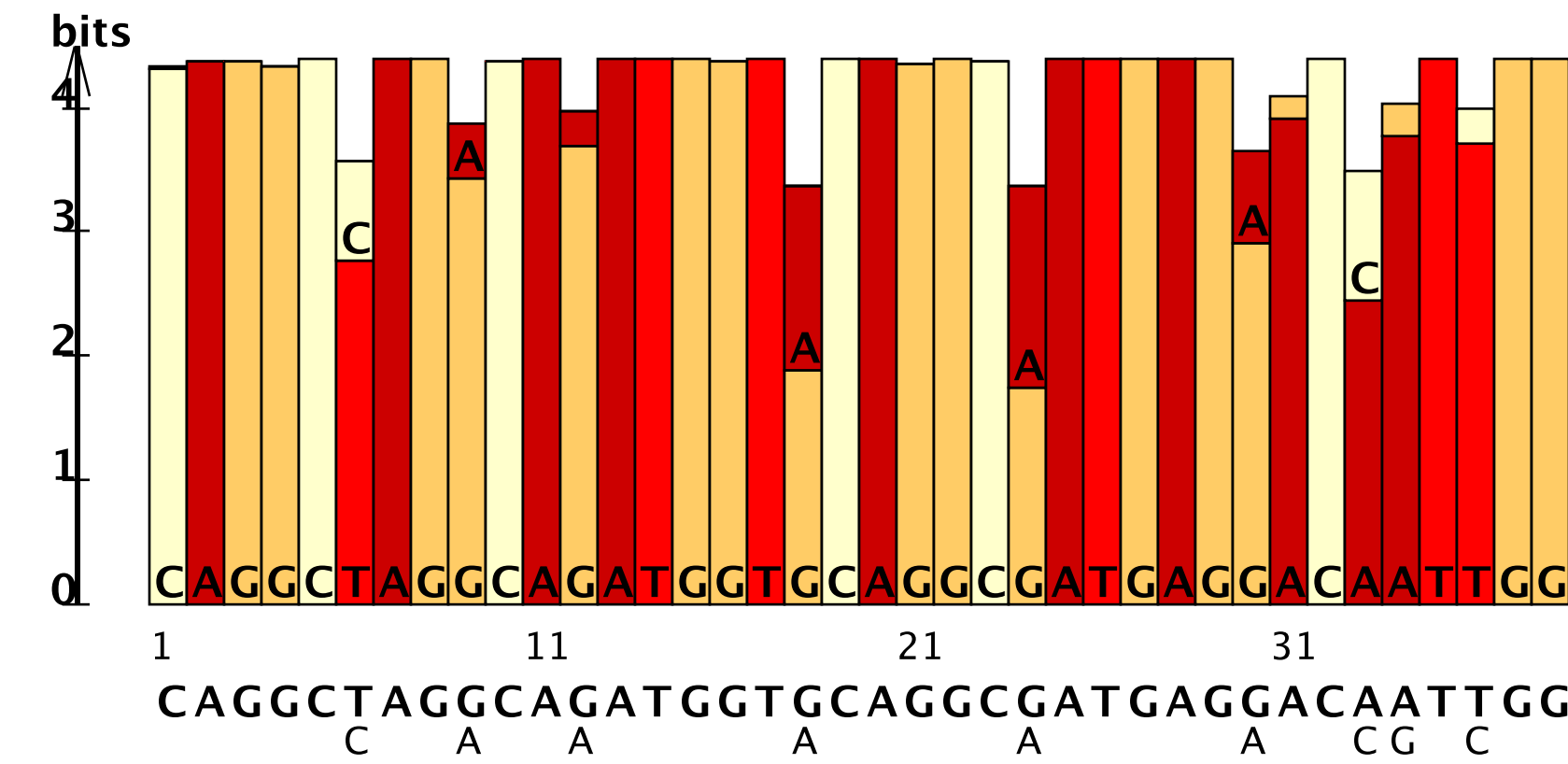

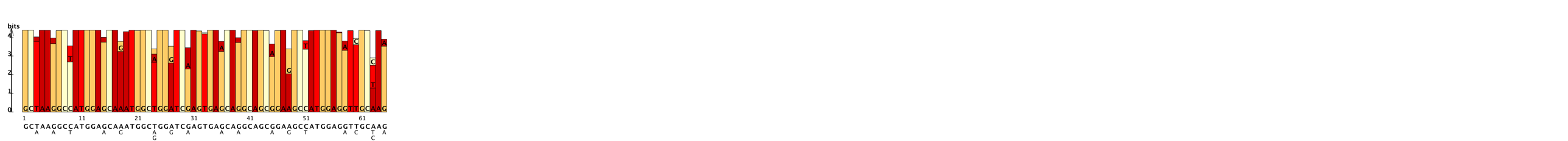

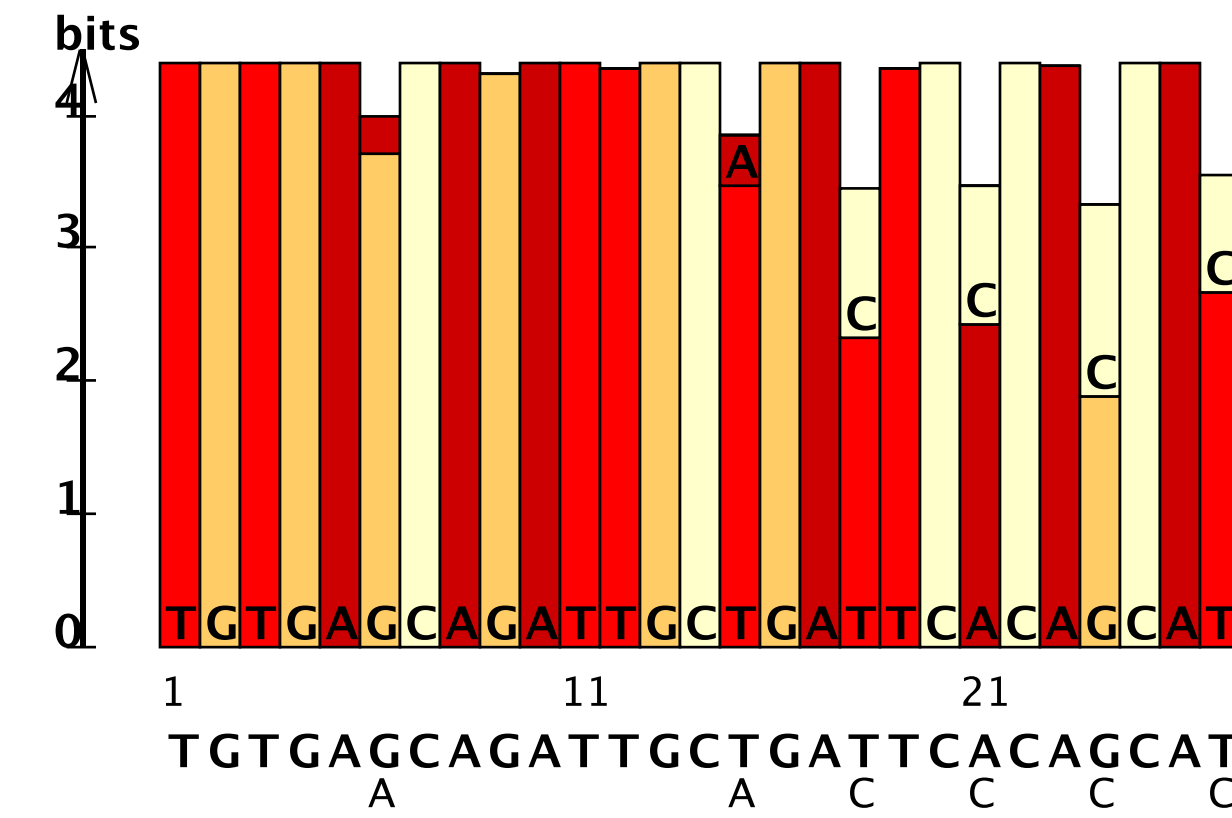

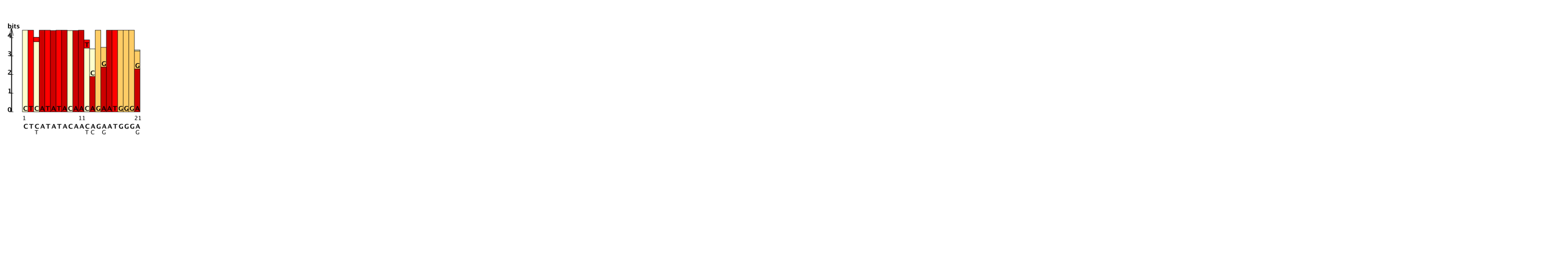

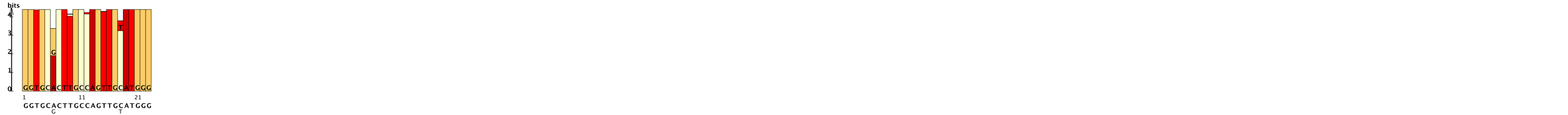

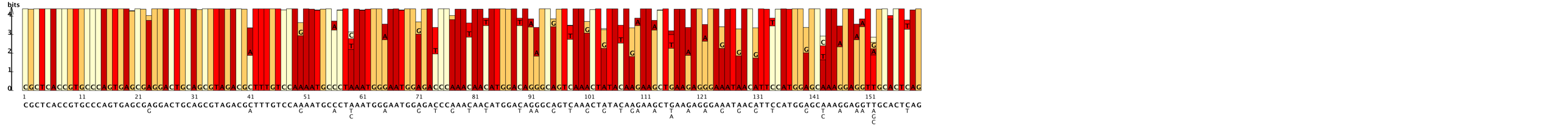

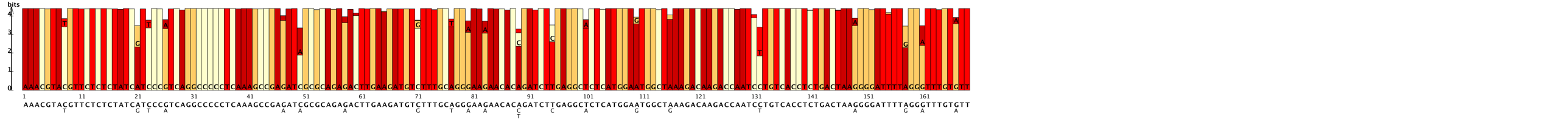

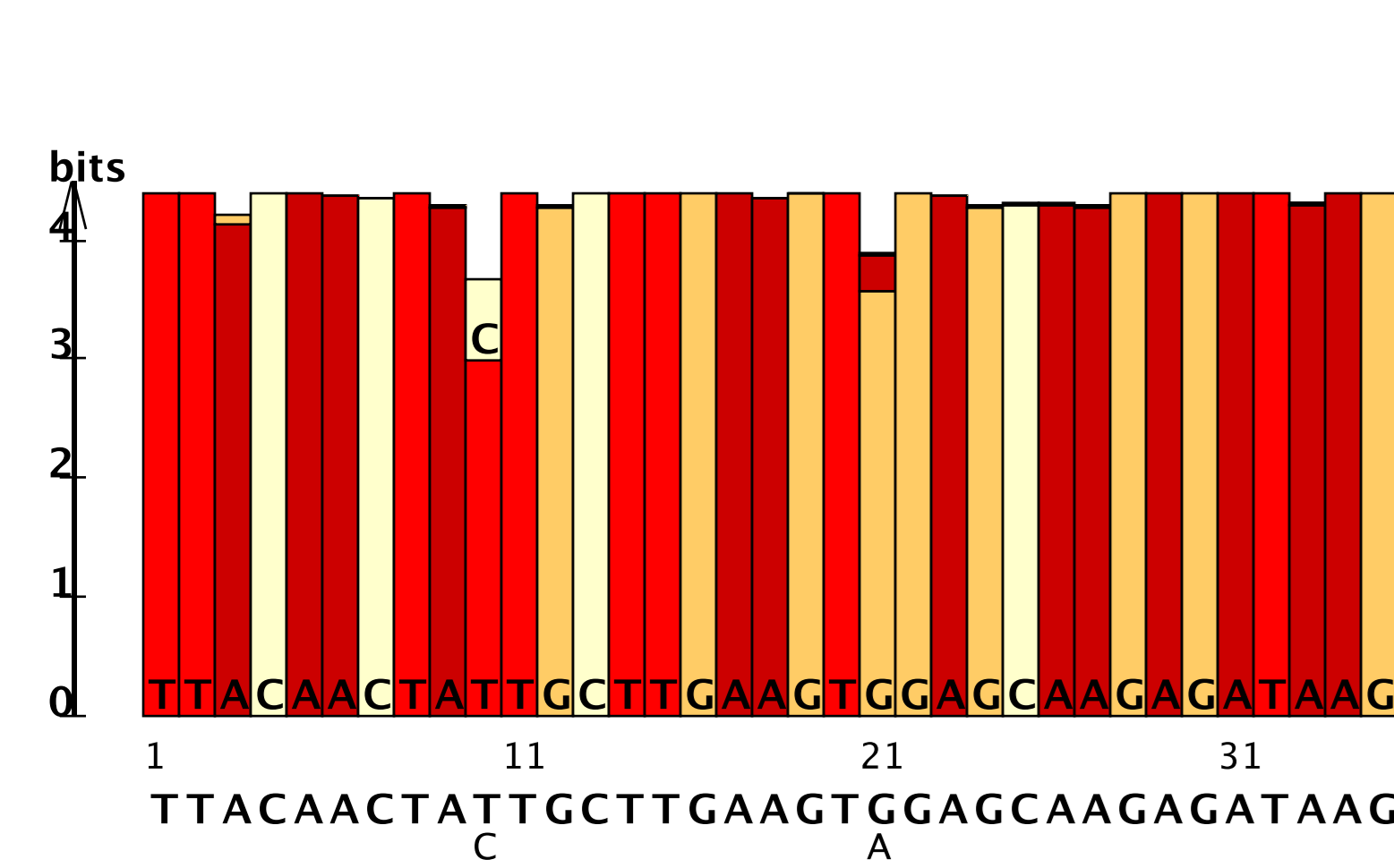

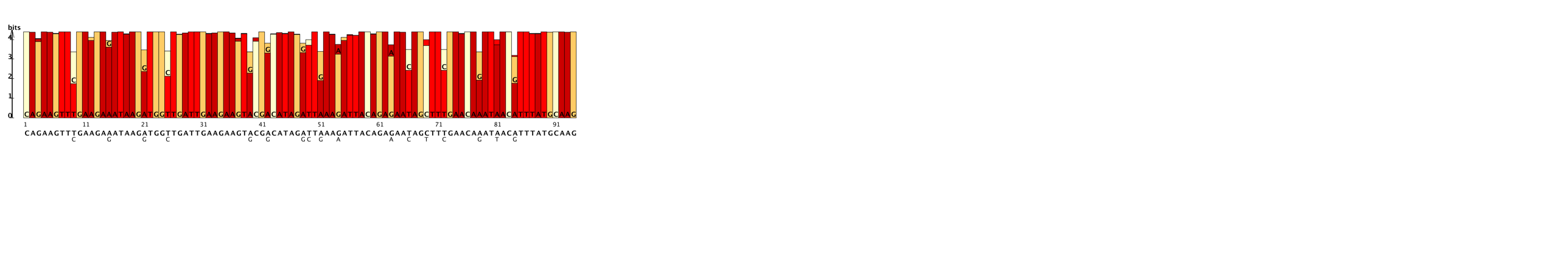

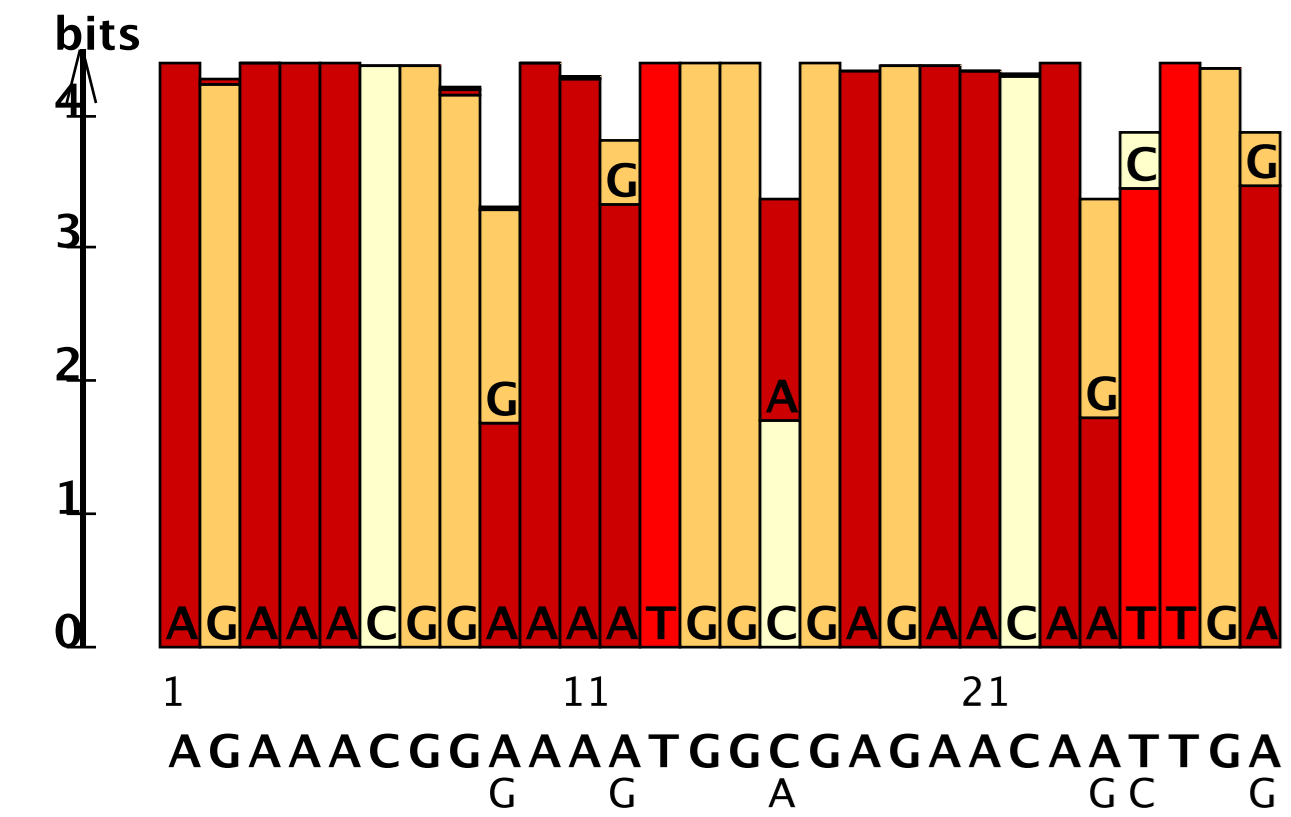

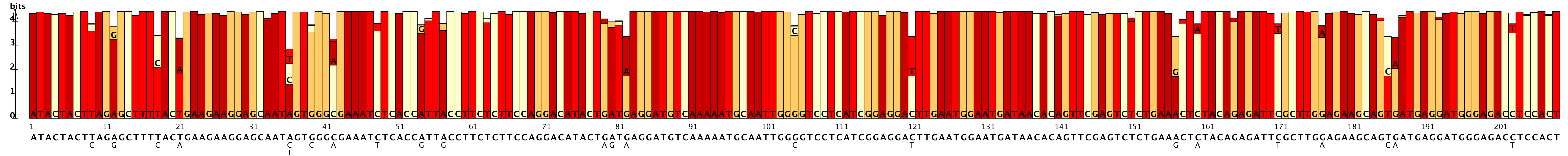

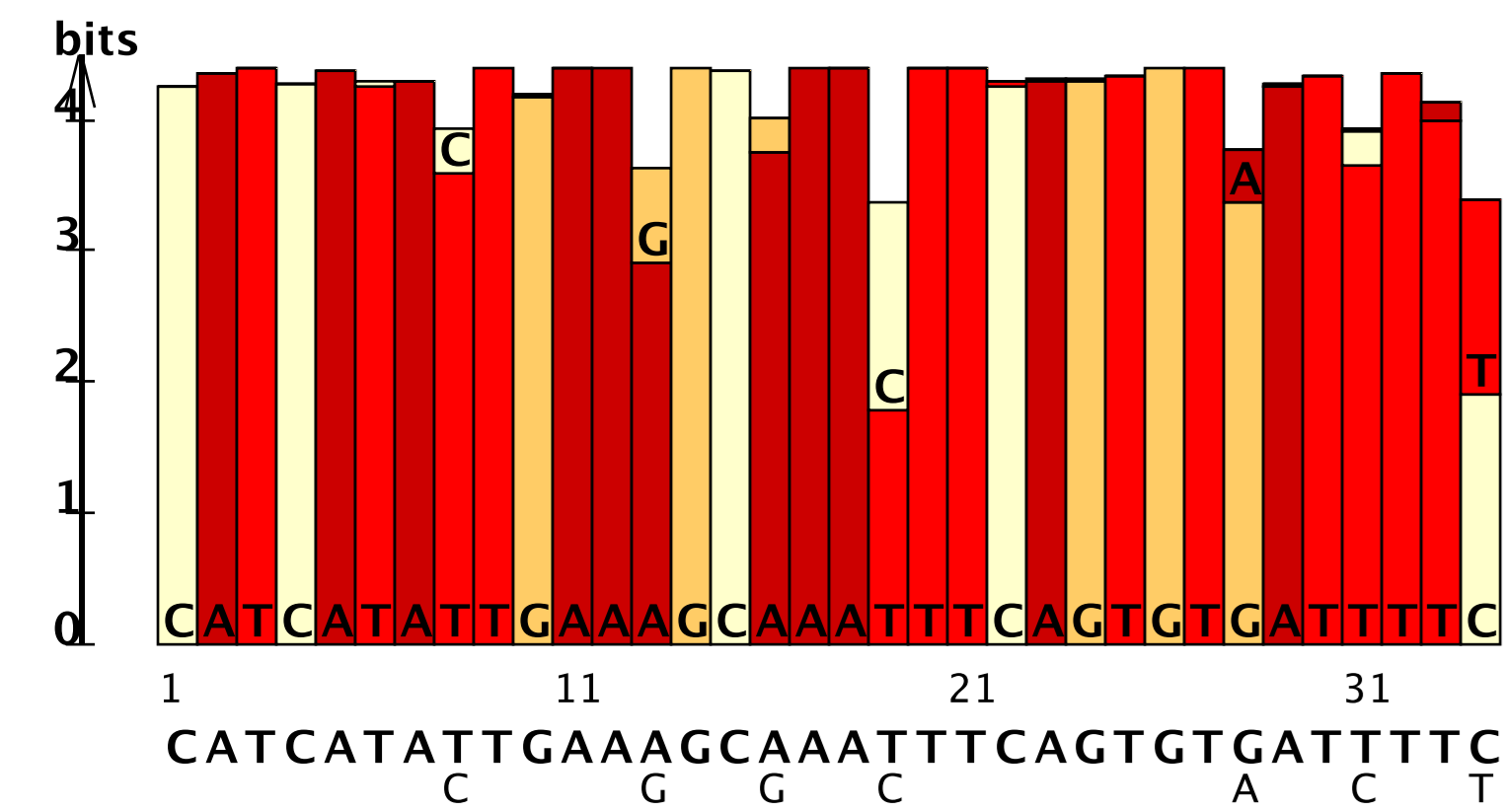

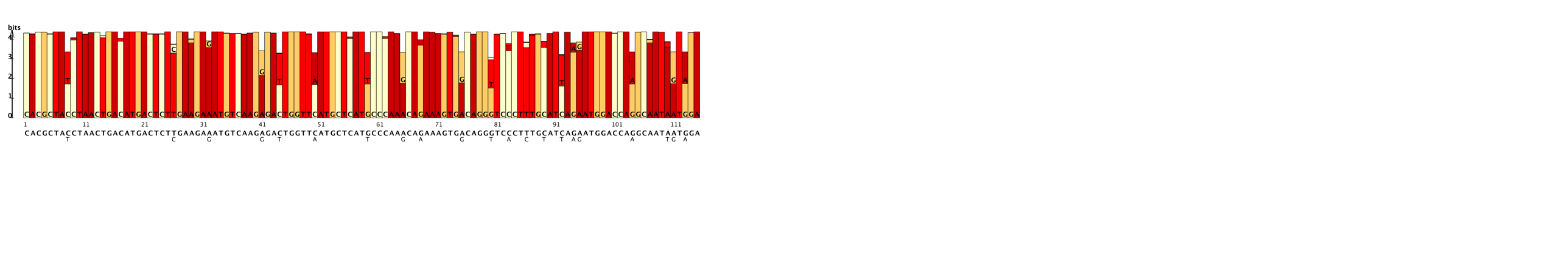

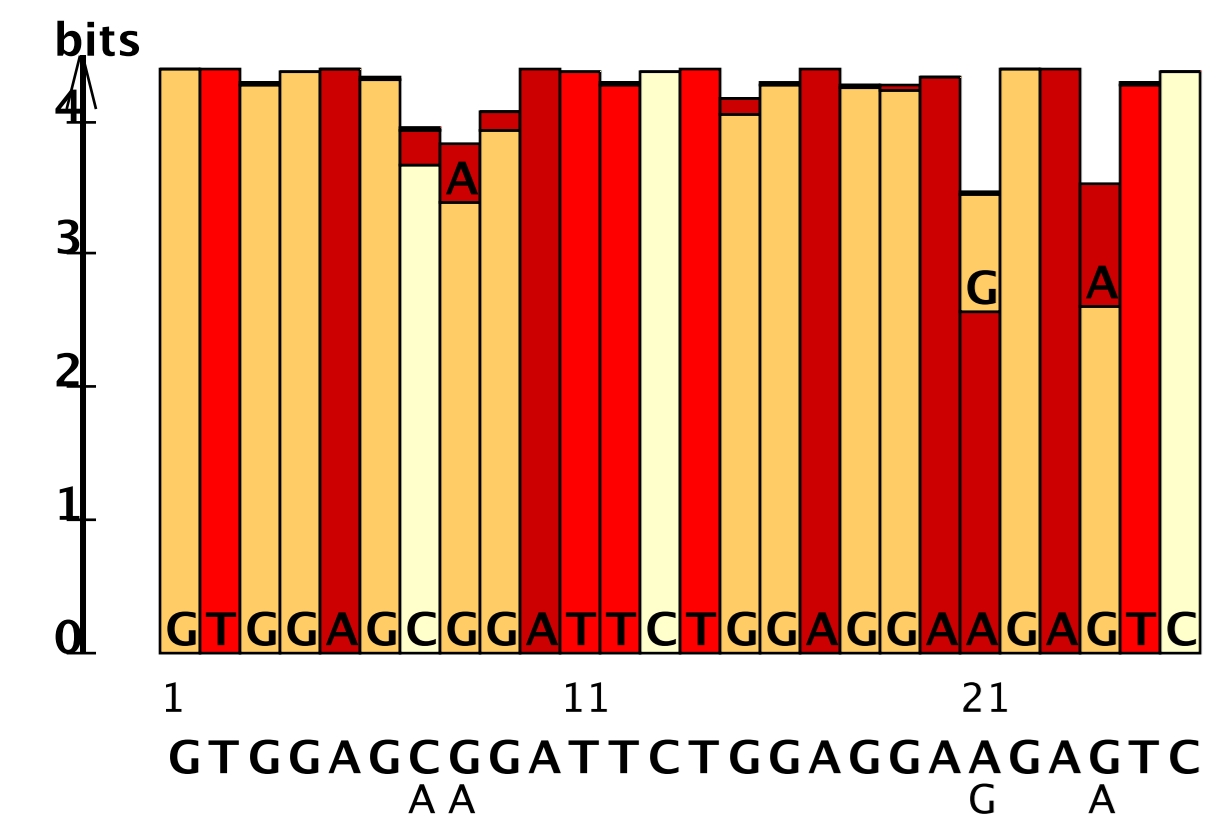

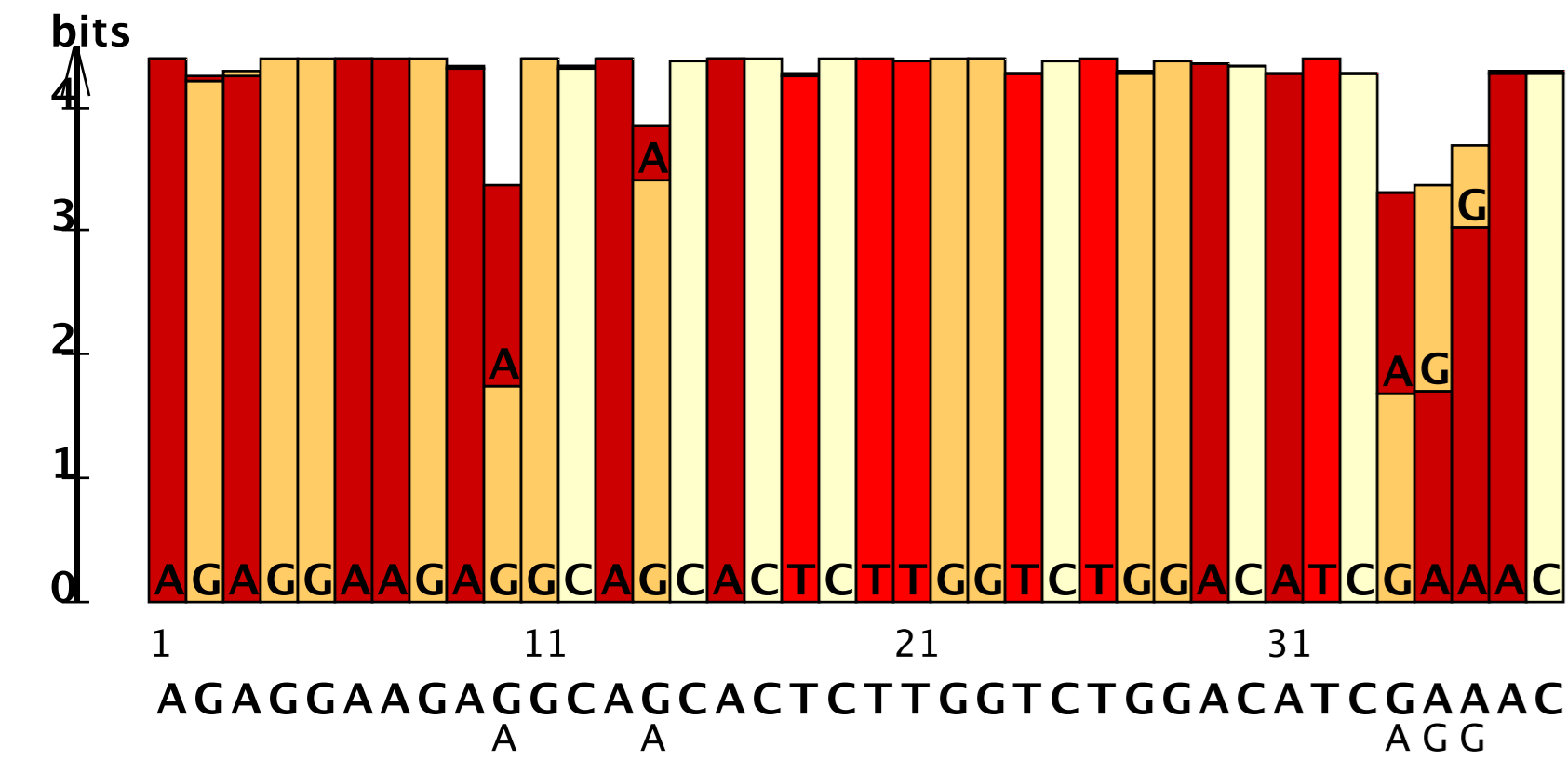

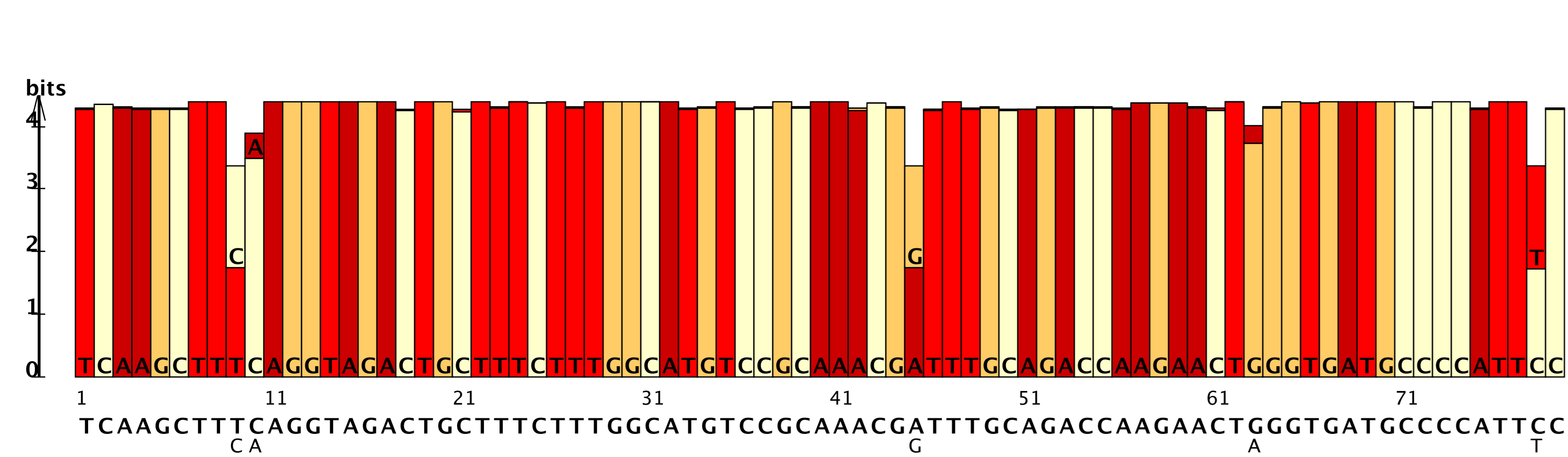

Supplement: Additional file 8 — Logobars of conserved regions. Eighty-seven logobars of conserved regions are shown sequentially. The columns with full information bits are the conserved ones, and those with partial information bits are variable. [file 1743-422X-8-44-S8.PDF]
